# Chiral Monoamidines as Effective Organocatalysts for the Stereoselective Synthesis of Oxindoles under Homogeneous and Heterogeneous Conditions

**DOI:** 10.1021/acs.joc.5c01894

**Published:** 2025-11-24

**Authors:** Sofia Toldo, Lorenzo Poletti, Graziano Di Carmine, Carmela De Risi, Alessandro Massi, Daniele Ragno

**Affiliations:** † Department of Environmental and Prevention Sciences, 9299 University of Ferrara, Via L. Borsari, 46-44121 Ferrara, Italy; ‡ Department of Chemical, Pharmaceutical and Agricultural Sciences, 9299 University of Ferrara, Via L. Borsari, 46-44121 Ferrara, Italy

## Abstract

The asymmetric aza-Henry reaction of aryl nitromethanes with isatin-derived ketimines promoted by chiral monoamidine catalysts is herein reported. This class of chiral Bro̷nsted bases, developed by Johnston, was first employed in the stereoselective synthesis of biologically relevant quaternary carbon-centered oxindoles. The process was first investigated under homogeneous conditions, affording a yield up to 95%, an *ee* up to 96%, and a dr up to 99:1. Next, the protocol was transposed to heterogeneous conditions using our recently developed polystyrene-supported 3-pyrrolidinol-linked catalyst **PS-(** ***S*** **)-Pyr-MAM**. The macroporous heterogeneous catalyst was tested in the synthesis of a short library of chiral oxindoles, showing activity almost comparable to that of the homogeneous counterpart, apart from an observed slight decrease in enantioselectivity (ca. 3–8% *ee*). The robustness of the synthetic protocol was confirmed on the gram-scale, and the recyclability of the catalyst was evaluated using straightforward filtration, achieving a satisfactory 84% *ee* after six cycles, with a modest decline in the conversion efficiency of approximately 2% per cycle, leading to a total turnover number of 53.1.

## Graphical abstract

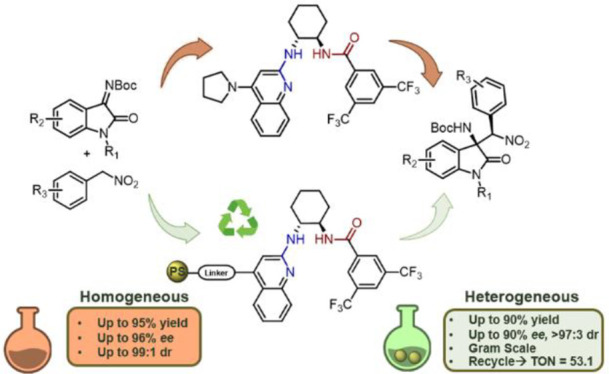

## Introduction

Chiral oxindoles are relevant structural motifs widely diffused in natural products and bioactive compounds.^1–3^ In particular, chiral 3-substituted 3-amino-2-oxindoles, with a quaternary stereogenic center at the 3-position, are acknowledged as preferred substructures in novel drug development, and the advancement of effective techniques for synthesizing this class of compounds is highly demanded.^4–9^ Indeed, significant pharmaceutically active molecules that contain this pivotal scaffold are consistently being identified. As representative examples (Figure 1), AG-041R is an antagonist of the gastrin/cholecystokinin-B receptor, effective in the healing of certain cartilage defects,^10,11^ SSR-149415 is an orally active nonpeptide antagonist of the vasopressin V1b receptor^12,13^ and NITD609 is an efficient antimalarial agent.^14–16^

**Figure 1:**
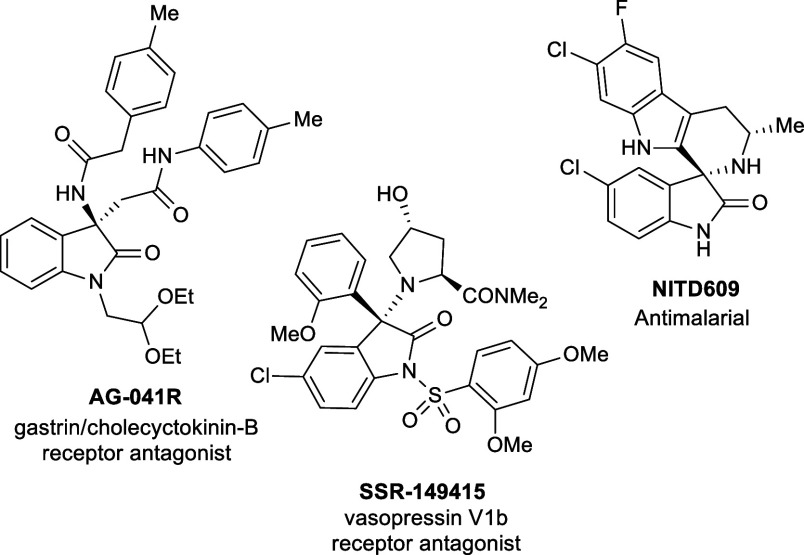
Example of biologically relevant 3-substituted 3-amino-2-oxindoles.

In light of the synthetic relevance of this class of compounds, several stereoselective synthetic protocols have been developed so far,^3,17–21^ relying mainly on nucleophilic addition to isatin-derived ketimines,^9,22–24^ substitution of 3-substituted oxindoles bearing a leaving group on C-3,^25–28^ direct amination of 3-substituted oxindoles,^29–31^ functionalization of 3-aminooxindoles,^32–36^ and intramolecular arylation.^37,38^ Extensive scientific production has also been reported for stereoselective tandem and domino processes.^39^ Among the aforementioned synthetic strategies, the asymmetric nucleophilic addition to isatin-derived ketimine represents one of the most efficient and straightforward routes, widely investigated by metal- and organocatalysis.^9,22,40^ In this regard, the stereoselective aza-Henry reaction,^41^ also known as the nitro-Mannich reaction, of isatin-derived ketimines represents a highly effective and selective method for synthesizing chiral 3-substituted 3-amino-2-oxindoles bearing a quaternary stereocenter.^9,18,22,42^ Despite the intrinsic lower reactivity and difficult enantiocontrol of isatin-derived ketimines compared to aldimines, different classes of asymmetric catalysts have recently been identified for the aza-Henry reaction involving isatin-derived ketimines: quinine-derived bifunctional organocatalysts,^43,44^ bis­(imidazolidine)­pyridine-NiCl_2_ complexes,^45^ Cu (II)-BOX complexes,^46,47^ macrocyclic Cu­(II)-salen complexes,^48^ bifunctional guanidine-amide,^49^ and trifunctional sulphonamide^50^ organocatalysts. Surprisingly, the application of the aforementioned catalytic species is restricted to fully aliphatic nitroalkanes as coupling partners. On the other hand, the stereoselective addition of aryl nitromethanes, which could allow the introduction of aromatic motifs frequently found in bioactive and natural products, has been scarcely reported and is limited to the scientific works of the groups of Meggers^51^ and Duan.^52,53^ The former study, conducted in 2015, represents the first example of an asymmetric aza-Henry reaction between isatin-derived *N*-Boc ketimines and aryl nitromethanes, promoted by a chiral Bro̷nsted base octahedral biscyclometalated iridium­(III) complex (Scheme 1a, **Λ**–**IrBB**). The methodology encompasses the straightforward formation of the kinetic product after 24 h working under reduced temperature with very low catalyst loading (0.5 mol %), followed by basic treatment leading to epimerization to the thermodynamically favored diastereomer. Despite the low catalytic loading and the applicability to a wide variety of coupling partners with high levels of enantioselectivity (90–98% *ee*) and diastereoselectivity (89:11–96:4), the use of an expensive and unrecyclable chiral iridium-based complex represents a main drawback of the process in terms of cost and sustainability. To overcome this issue, in 2018 and 2019, Duan developed two different organocatalytic processes, by using bifunctional hydrogen-bonding phase-transfer catalysts derived from *Cinchona* alkaloid^52^ (Scheme 1b, **PTC-1**) and *L*-*tert*-leucine (Scheme 1c, **PTC-2**).^53^ The protocols required the use of a 10 mol % catalyst loading working at low temperatures (≤−30 °C), displaying good values in terms of yields (>85%) and stereoselectivities (86:14–95:5 dr, 79–95% *ee* for **PTC-1**; and 79:21–96:4 dr, 83–94% *ee* for **PTC-2**). Unlike Meggers’s catalyst, which presented a Bro̷nsted basic site, Duan’s protocols needed an overstoichiometric amount of base (5 eq. LiOH H_2_O) to generate the corresponding nitronates from aryl nitromethanes by deprotonation; while this did not seem to impact the efficiency based on the data provided, it is important to acknowledge that it could represent a significant issue when dealing with base-sensitive substrates.

**Scheme 1:**
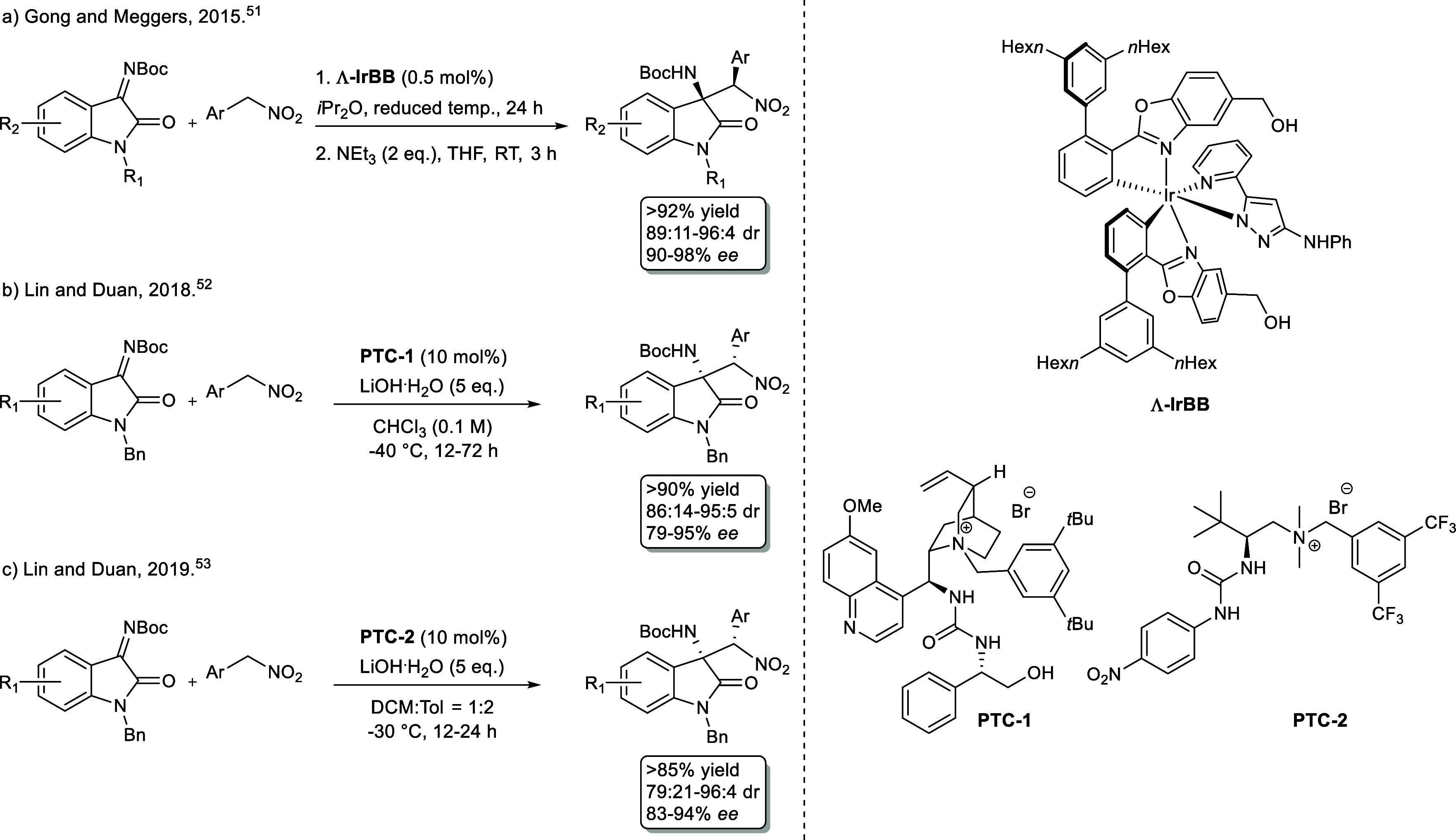
Aza-Henry Reaction of Isatin-Derived Ketimines with Aryl Nitromethanes for the Stereoselective Synthesis of 3-Substituted 3-Amino-2-oxindoles: State-of-the-Art

In light of the reported state-of-the-art technology and the synthetic relevance of the target chiral oxindoles, the development of an alternative organocatalyzed protocol for the stereoselective aza-Henry reaction of aryl nitromethanes with isatin-derived ketimines is highly desirable. Moreover, all of the reported approaches take place in the homogeneous phase, preventing facile catalyst recovery and reuse. Taking into account that the synthesis of chiral catalytic systems is costly and time-consuming, and their use is generally limited to a single reaction run, catalyst immobilization on inert supports represents a viable approach for allowing easy catalyst recovery by simple filtration and reuse for several cycles, improving process efficiency and sustainability.^54^

Recently, driven by our research interest in the field of organocatalysis under a heterogeneous phase,^55–61^ we reported on the immobilization of a chiral mono­(amidine) catalyst (**MAM**) developed by Johnston and co-workers^62^ for the stereoselective synthesis of nonsymmetric *cis*-stilbene Nutlins precursors through an asymmetric aza-Henry reaction between aryl nitromethanes and *N*-Boc imines (Figure 2, top).^63^ This class of chiral catalyst acts as a Bro̷nsted base, through a bifunctional hydrogen-bonding activation mechanism, by virtue of the synergistic action of a single amidinic moiety (Figure 2, blue) coupled to an amide functionality (Figure 2, red).^62^ Surprisingly, according to the literature, the use of **MAM** has been limited only to the stereoselective synthesis of nonsymmetric *cis*-stilbene Nutlins precursors^62^ and to the enantioselective reduction of nitroalkenes.^64^ Considering the catalyst structure and activation mechanism, together with the excellent performance of the supported version **PS-(** ***S*** **)-Pyr-MAM** (Figure 2) in the asymmetric aza-Henry with *N*-Boc imines, we wondered whether chiral monoamidine could be efficiently employed in the stereoselective aza-Henry reaction between aryl nitromethanes and isatin-derived *N*-Boc ketimines.

**Figure 2:**
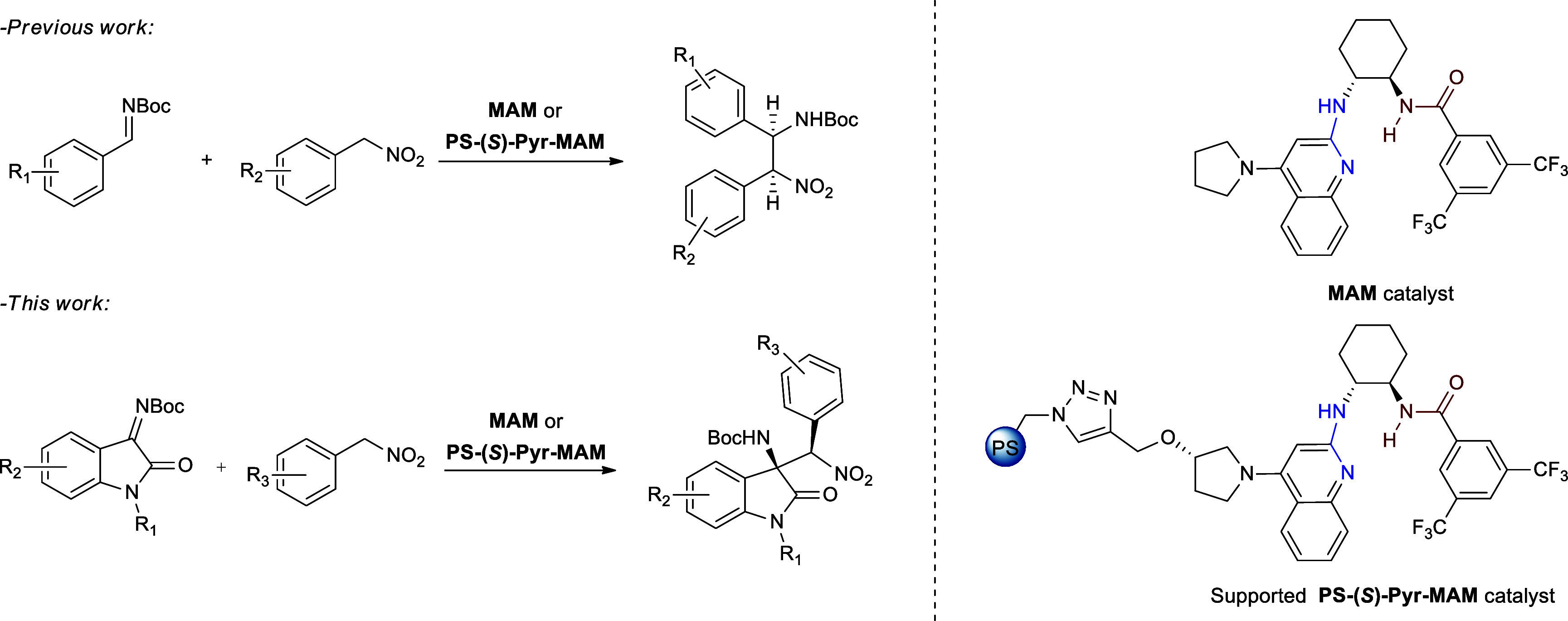
Previous work and present study on asymmetric aza-Henry reactions promoted by homogeneous MAM and heterogeneous PS-(*S*)-Pyr-MAM chiral monoamidine catalysts.

For these reasons, we herein report the application of chiral monoamidines as effective catalysts for the stereoselective synthesis of 3-substituted 3-amino-2-oxindoles (Figure 2, bottom). The activity of the homogeneous **MAM** catalyst was first assessed during process optimization in the asymmetric aza-Henry reaction between *N*-Boc isatin-derived ketimines and aryl nitromethanes. Once the optimized conditions were established, the reaction scope was investigated, achieving yields up to 95%, an *ee* up to 96%, and a dr up to 99:1. The catalytic performances of the supported counterpart **PS-(** ***S*** **)-Pyr-MAM** were then evaluated in the same reaction, synthesizing a short library of chiral 3-substituted 3-amino-2-oxindoles with yields up to 90%, an *ee* up to 90%, and a dr up to 97:3. Finally, the robustness of the methodology was confirmed on the gram-scale, and the recyclability of the supported catalyst was investigated, yielding a satisfactory 84% *ee* after six cycles, together with a slight yield decrease (81% vs 92% of the first run).

## Results and Discussion

Our study started from the investigation of the aza-Henry reaction between the isatin-derived ketimine **1a** and aryl nitromethane **2a**, affording the target oxindole **3aa** (Table 1). Drawing on our experience in stereoselective aza-Henry processes^63^ and on the reported literature procedures,^51–53^ the reaction was conducted at −30 °C for 48 h using **MAM** (10 mol %) as the catalyst, evaluating the reaction yield and stereoselectivity. Under these conditions, the first solvent study was performed, screening dichloromethane (DCM), tetrahydrofuran (THF), biobased cyclopentyl methyl ether (CPME), and toluene (entries 1–4) as potential solvents. The reaction proceeded smoothly with almost full yield in all of the tested solvents, exhibiting the best stereoselectivity in toluene, with excellent 92% *ee* and 92:8 dr (entry 4). Next, the temperature effect was evaluated (entries 5–6), confirming the absolute necessity of working at low temperatures (−30 °C) to achieve higher levels of stereocontrol; however, further temperature decrease led to a longer reaction time without enantiocontrol enhancement (entry 7). At this stage of the study, the reaction time was reduced to 24 h, exhibiting a clear yield decrease (71% yield, entry 8), also when a slightly higher amount of the **MAM** catalyst (15 mol %) was employed (81% yield, entry 9). Finally, to exclude the presence of a potential uncatalyzed background process, the reaction was performed in the absence of the catalyst (entry 10), without detecting any traces of desired oxindole **3aa.**

**Table 1:**
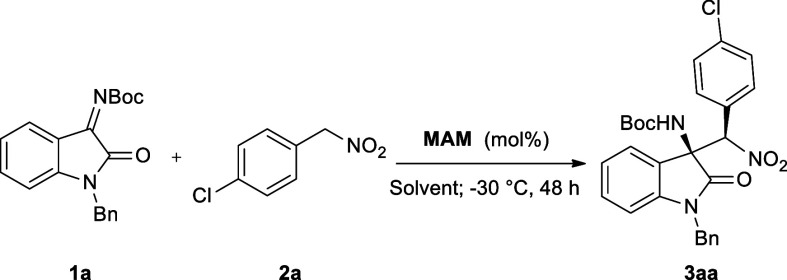
Reaction Optimization under Homogeneous Conditions^a^

entry	catalyst (mol %)	solvent	yield (%)^b^	*ee* (%)^c^	dr^c^
1^d^	**MAM** (10)	DCM	86	78	89:11
2	**MAM** (10)	THF	>95	81	92:8
3	**MAM** (10)	CPME	>95	83	93:7
**4**	**MAM** **(10)**	**toluene**	**>95**	**92**	**92:8**
5^d^	**MAM** (10)	toluene	>95	80	92:8
6^e^	**MAM** (10)	toluene	>95	41	90:10
7^f^	**MAM** (10)	toluene	87	86	91:9
8^g^	**MAM** (10)	toluene	71	92	92:8
9^g^	**MAM** (15)	toluene	81	92	92:8
10	none	toluene			

a **1a** (0.20 mmol), **2a** (0.22 mmol), anhydrous solvent (2.0 mL).

b Detected by ^1^H NMR spectroscopy of the crude reaction mixture with acetonitrile as the external standard.

c Diastereomeric ratio (dr) and enantiomeric excess (*ee*) were determined by chiral HPLC after the workup.

d *T* = −20 °C.

e *T* = 0 °C, 16 h.

f *T* = −50 °C, 72 h reaction time.

g 24 h reaction time.

From the structural point of view, the product configuration is in agreement with the data reported in the literature by Meggers and co-workers,^51^ accounting for a *Si*-face/*Si*-face attack of the nitronate nucleophile to the ketimine electrophile (Figure 3). According to the activation mechanism proposed by Johnston and co-workers for aryl nitromethane and aryl *N*-Boc imine by the bifunctional monoamidine (**MAM**),^62^ and in line with the computational studies conducted by Dudding and co-workers on the bis-amidine (**BAM**)-catalyzed aza-Henry reaction,^65,66^ a tentative mechanism can be envisaged for the asymmetric aza-Henry reaction of aryl nitromethanes with *N*-Boc isatin-derived ketimines (Figure 3). The amidinium ion generated from the direct deprotonation of aryl nitromethane coordinates the isatin-derived ketimine by a homonuclear positive-charge-assisted hydrogen bond (homonuclear (+)­CAHB), while the resulting nitronate would pair with the amide proton through polar covalent hydrogen bonding. According to Dudding and co-workers,^65^ an aryl-directing effect consisting in a π–π stacking of the aryl moieties could be responsible for the observed diastereoselection. In support of the proposed mechanism, two control experiments were conducted under optimized conditions (Table 1, entry 4), using methanol as a representative hydrogen-bond-donating solvent and dimethylformamide as a representative hydrogen-bond-accepting solvent. To our delight, the use of potential disruptive hydrogen-bonding solvents led to a marked enantioselectivity decrease in both cases (MeOH: 23% *ee,* 97:3 dr; DMF, 25% *ee,* 97:3 dr), thus confirming the key role of catalyst/substrate hydrogen-bonding interactions in the stereodetermining transition state (Figure 3).

**Figure 3:**
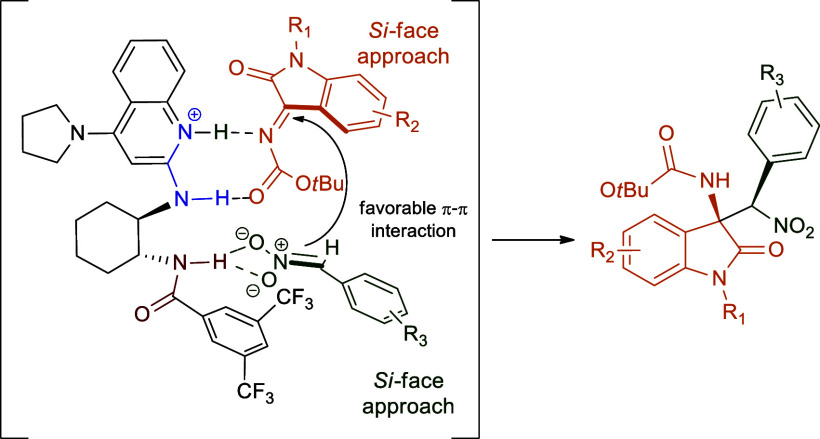
Proposed catalyst activation mechanism.

With the optimal conditions in hand (1.1 equiv of aryl nitromethane, **MAM** 10% mol, anhydrous toluene, −30 °C, 48 h), the substrate scope was then investigated (Table 2). Different aryl nitromethanes **2** displaying electron-withdrawing and electron-donating groups at the *para-* and *meta*-positions were first tested with *N*-Boc isatin-derived ketimine **1a** as the coupling partner. *Para-*substituted aryl nitromethanes (entries 1–3) furnished the target oxindole **3** with satisfactory yields (79–91%) and high to excellent stereoselectivity (85–96% *ee,* 92:8–99:1 dr), providing the best results in the case of *p*-methyl-substituted aryl nitromethane **2c** (entry 3). Next, *meta*-substituted aryl nitromethanes were reacted, exhibiting almost full conversion for all of the substrates, with higher levels of stereoselectivity (92% *ee,* 93:7 dr) for *m-*chloro-substituted aryl nitromethane **2d** (entry 4) and poorer enantioselectivity (71–76% *ee*) for *m*-bromo- and *m*- methyl-substituted substrates (entries 5–6). Next, unsubstituted phenyl nitromethane **2g** was tested, leading to an almost quantitative yield with good diastereoselectivity (93:7) and modest enantioselectivity (73% *ee*, entry 7). At this stage of the study, the effect of the heterocyclic nitrogen substitution was evaluated by employing aryl nitromethane **2a** as a coupling partner. In this regard, unsubstituted ketimine **1b** led to the almost quantitative formation of oxindole **3ba** (84% yield, entry 8) with satisfactory enantioselectivity (80% *ee*) and decreased diastereoselectivity (72:28 dr). *N*-Methyl substitution was also tolerated, affording the corresponding product with enhanced stereoselectivity (84% *ee*, 92:8 dr; entry 9). Next, substitution on the aromatic isatin ring was considered, introducing electron-withdrawing and electron-donating groups (Cl and OMe) at the C5 and C6 positions (entries 10–13). The corresponding products were obtained with high yields (85–95%), diastereo- (87:13–97:3 dr), and enantioselectivity (85–90% *ee*). Next, we investigated the synthesis of oxindole **3bg**, from unsubstituted ketimine **1b** and phenyl nitromethane **2g**. Interestingly, the chiral phase-transfer protocol of Duan and co-workers^52^ totally failed in the synthesis of this derivative, while the **MAM** catalytic system efficiently promoted the transformation, leading to an almost quantitative yield (91%) and satisfactory stereoselectivity (82% *ee*, 95:5 dr). Finally, the reactivity of alkyl nitromethanes was investigated using nitromethane as a representative substrate in the coupling with ketimine **1a**. After a reaction optimization study (Supporting Information, Table S1), the best compromise between product yield and *ee* was found working in toluene at 0 °C, with an excess of nitromethane (9 equiv). Unfortunately, the reaction proceeded in full yield but with unsatisfactory enantioselectivity (55% *ee*, entry 3).

**Table 2:**
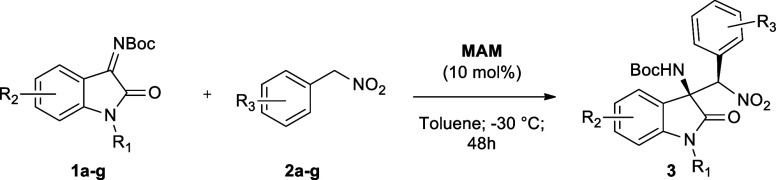

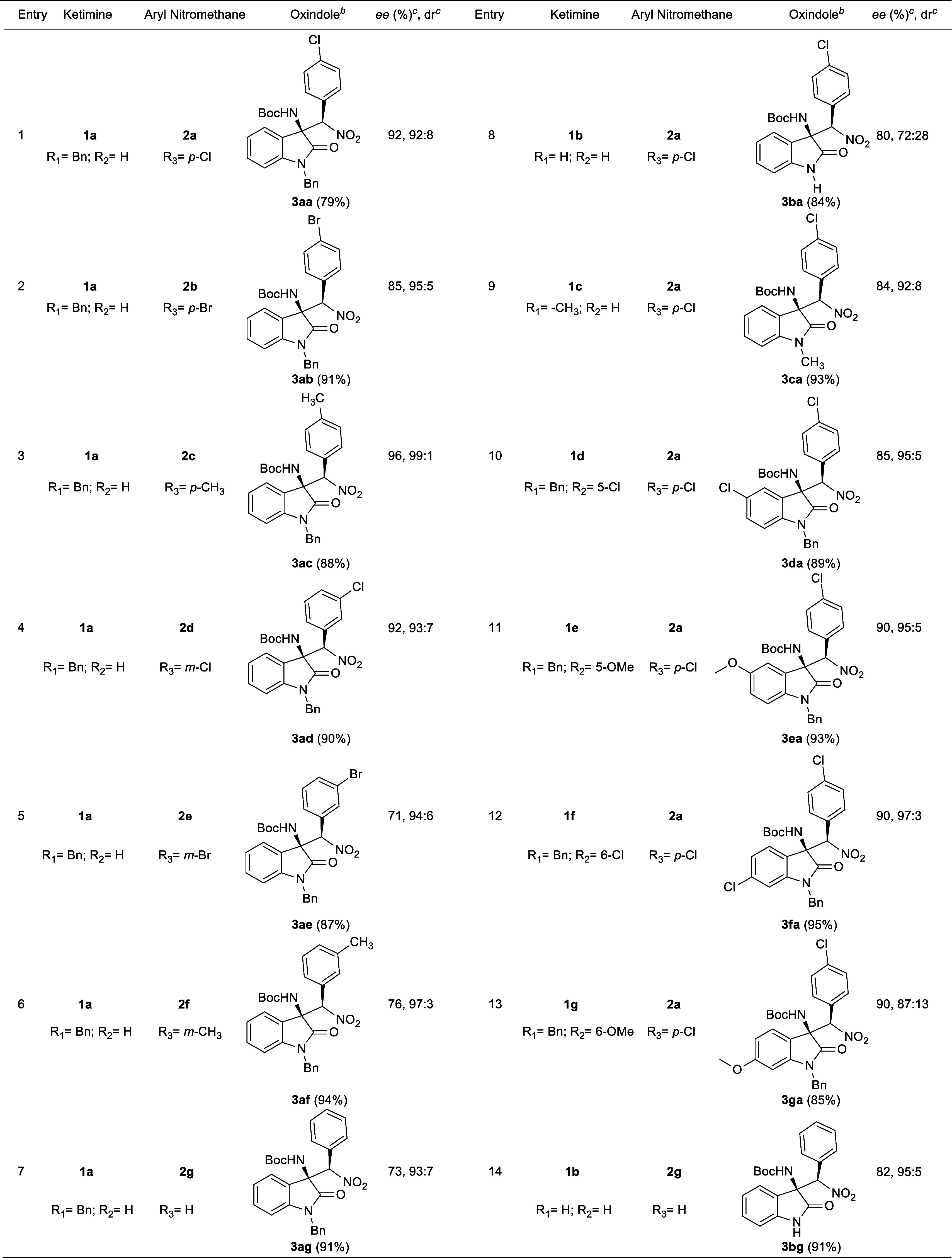
Reaction Scope under Homogeneous Conditions^a^

a **1** (0.20 mmol), **2** (0.22 mmol), anhydrous toluene (2.0 mL).

b Isolated yield.

c Diastereomeric ratio (dr) and enantiomeric excess (*ee*) were determined by chiral HPLC.

Once **MAM** catalyst efficiency was established over a broad substrate scope, driven by our experience in heterogeneous-phase asymmetric organocatalysis,^55–61,63^ we wondered whether our recently discovered polystyrene-supported chiral monoamidine catalyst **PS-(** ***S*** **)-Pyr-MAM** (Figure 2) could be efficiently exploited in the synthesis of chiral oxindoles **3** under a heterogeneous phase. **PS-(** ***S*** **)-Pyr-MAM** represents the immobilized version of the **MAM** catalyst, solidly covalently bonded to the macroporous polystyrene support through an (*S*)-3-pyrrolidinol-linker. Among different supported catalysts tested, it displayed the best performances in terms of selectivity, robustness, and recyclability in the synthesis of a library of β-amino nitroalkane Nutlins precursors.^63^ For the aforementioned reasons, it was next tested in the benchmark aza-Henry reaction between isatin-derived ketimine **1a** and aryl nitromethane **2a** under the heterogeneous phase (Table 3), with the aim to enhance the process efficiency and sustainability by taking advantage of catalyst simple recovery and recyclability.^54,67^

**Table 3:**
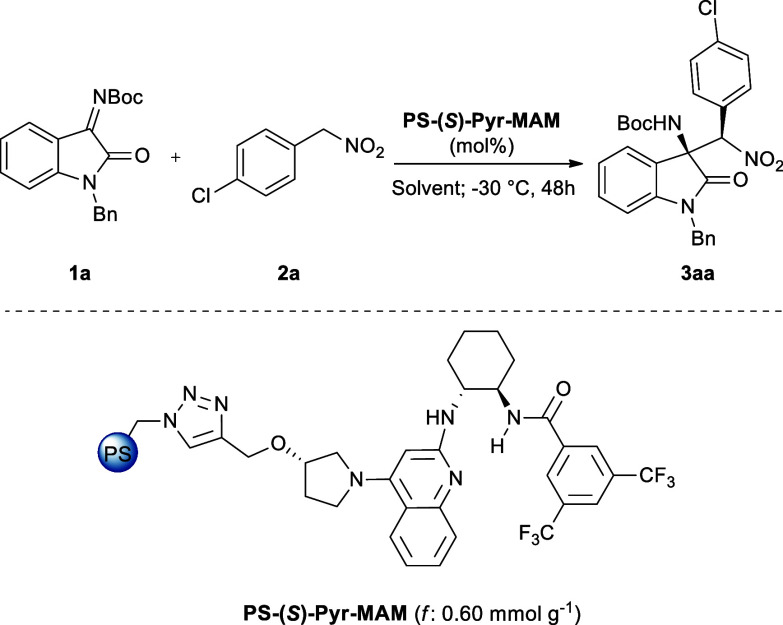
Reaction Optimization under Heterogeneous Conditions^a^

entry	catalyst (mol %)	solvent	yield (%)^b^	*ee* (%)^c^	dr^c^
1	(10)	DCM	79	74	88:12
2	(10)	THF	>95	77	91:9
3	(10)	CPME	>95	79	91:9
**4**	**(10)**	**toluene**	**>95**	**89**	**95:5**
5^d^	(10)	toluene	>95	84	93:7
6	(5)	toluene	75	75	92:8
7	(15)	toluene	>95	87	94:6

a **1a** (0.20 mmol), **2a** (0.22 mmol), anhydrous solvent (2.0 mL).

b Detected by ^1^H NMR spectroscopy of the crude reaction mixture with acetonitrile as the external standard.

c Diastereomeric ratio (dr) and enantiomeric excess (*ee*) were determined by chiral HPLC after the workup.

d **PS-(** ***R*** **)-Pyr-MAM** (*f*: 0.55 mmol g^–1^) was used as the heterogeneous catalyst.

Hence, the catalytic activity of **PS-(** ***S*** **)-Pyr-MAM** (*f*: 0.60 mmol g^–1^) was assessed, employing 10 mol % catalyst loading at −30 °C for 48 h. A solvent screening including DCM, THF, CPME, and toluene (entries 1–4) showed quantitative yields for the last three solvents and incomplete conversion for DCM (79%, entry 1). Regarding the stereoselectivity, the best results were achieved in toluene (entry 4, 89% *ee*, 95:5 dr), observing a behavior comparable to that of the homogeneous counterpart. At this stage of the study, we evaluated the influence of the chiral linker configuration, testing the (*R*)-3-pyrrolidinol- linked catalyst **PS-(** ***R*** **)-Pyr-MAM** (*f*: 0.55 mmol g^–1^) and observing a modest mismatch effect (entry 5). Finally, both increasing and decreasing the catalyst had a detrimental impact on the reaction outcome (entries 6–7), resulting in lower levels of enantio- and diastereoselectivity.

At this stage of the study, the scope of the aza-Henry reaction was investigated under the optimized heterogeneous conditions (**PS-(** ***S*** **)-Pyr-MAM** 10.0 mol %, toluene, −30 °C, 48 h). According to the homogeneous reaction scope, a short library of the most promising oxindoles **3** was prepared through the disclosed methodology under heterogeneous conditions (Table 4).

**Table 4:**
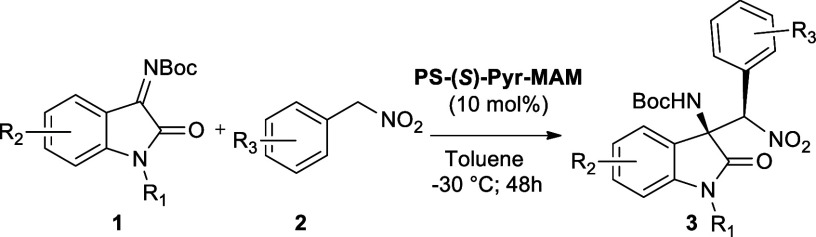

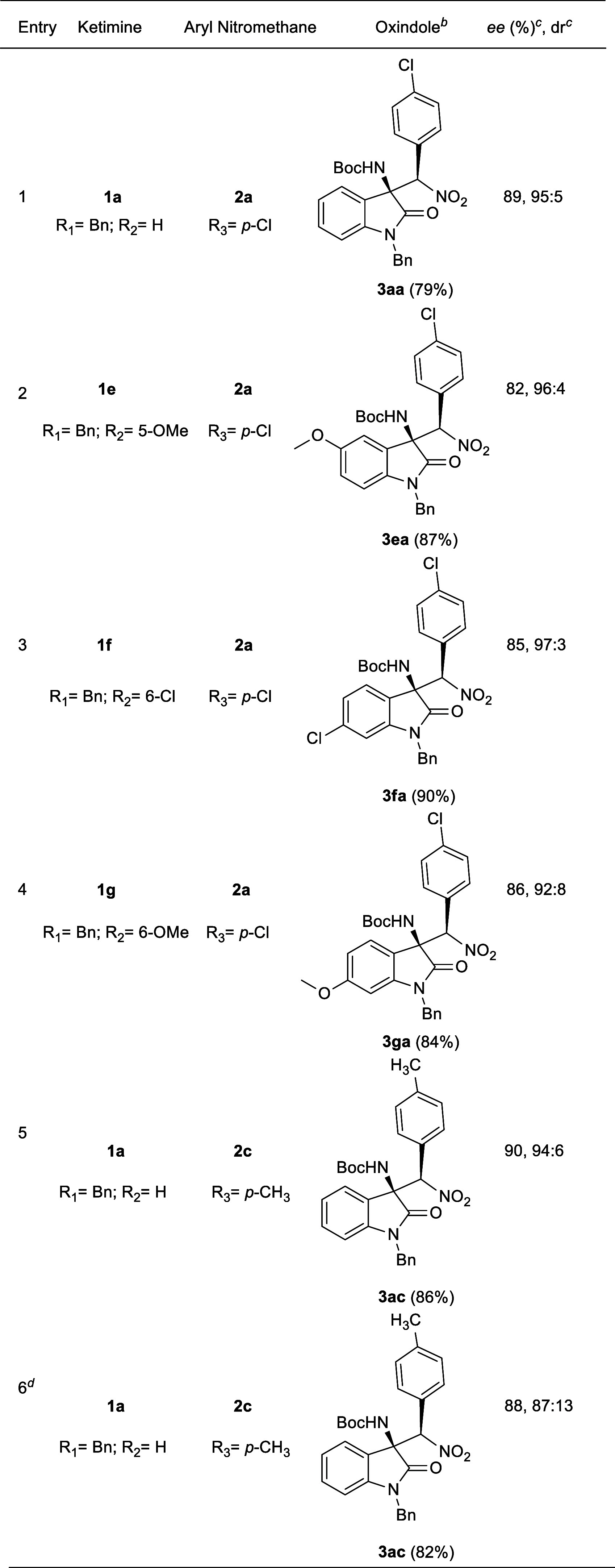
Reaction Scope under Heterogenous Conditions^a^

a **1** (0.20 mmol), **2** (0.22 mmol), anhydrous toluene (2.0 mL).

b Isolated yield.

c Diastereomeric ratio (dr) and enantiomeric excess (*ee*) were determined by chiral HPLC after the workup.

d **1a** (3.00 mmol), **2c** (3.30 mmol), anhydrous toluene (30.0 mL).

For all of the products, a behavior comparable to that of the homogeneous process was observed in terms of yield, thus proving the absence of mass transfer limitations. Regarding stereoselectivity, it is important to point out that for an immobilized chiral catalyst, the support and the nature of the ligand can somehow influence the catalyst stereocontrol capability.^68^ Gratifyingly, in our case, the stereoselectivities were only slightly decreased (ca. 3–8% *ee*) compared to the homogeneous process, while the diastereoselectivities remained almost unchanged or even increased, as in the case of **3ga** (homogeneous 87:13 dr vs heterogeneous 92:8 dr). Finally, the robustness of the heterogeneous-phase synthetic protocol was confirmed in the synthesis of **3ac** on the gram-scale (entry 6), obtaining a satisfactory 82% yield of the isolated product with 88% *ee* and 87:13 dr.

As previously mentioned, the most intriguing aspect of the process transition from the homogeneous to the heterogeneous phase is catalyst recyclability. Accordingly, **PS-(** ***S*** **)-Pyr-MAM** recyclability was evaluated in the synthesis of oxindole **3ac** from isatin-derived ketimine **1a** and *p*-Me-substituted aryl nitromethane **2c**. Simple centrifugation, filtration, and washing of the reaction mixture with toluene afforded the recovered catalyst, which was finally dried under reduced pressure and reused in the following reaction. **PS-(** ***S*** **)-Pyr-MAM** was used over six consecutive cycles (Figure 4) displaying a negligible 2% average yield decrease for the first five cycles with reproducible enantiocontrol (90–87% *ee*). The last cycle showed partial catalyst deactivation with a slightly diminished yield (81% vs 92% of the first run) and enantiocontrol (84% *ee* vs 90% *ee* of the first run), accounting for an accumulated turnover number (TON) of 53.1.

**Figure 4:**
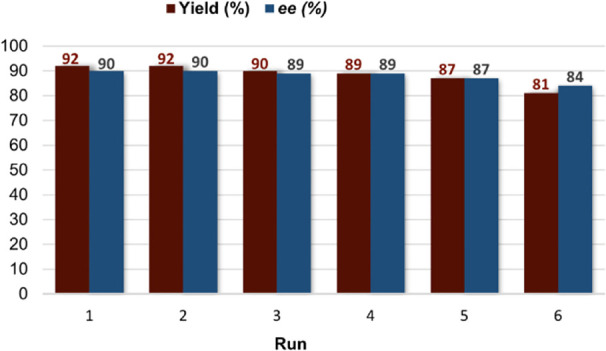
Recycling experiments (1*a*/2c coupling with 10.0 mol % of PS-(*S*)-Pyr-MAM).

## Conclusions

In summary, we report the application of chiral monoamidine catalysts in the diastereo- and enantioselective synthesis of biologically relevant 3-substituted 3-amino-2-oxindoles. This pivotal chiral scaffold was synthesized through the asymmetric aza-Henry addition of the less explored aryl nitromethanes with isatin-derived ketimines. The process was first investigated using the **MAM** catalyst under homogeneous conditions, exploiting a synergistic bifunctional hydrogen-bonding activation mechanism. After thorough screening of reaction conditions, the scope of the reaction was studied, achieving satisfactory values of yield, diastereo-, and enantioselectivity. With the aim to increase the process efficiency and sustainability, the corresponding heterogeneous-phase process was developed using our recently discovered polystyrene-supported 3-pyrrolidinol-linked catalyst **PS-(** ***S*** **)-Pyr-MAM**. The macroporous supported catalyst proved to be effective in the synthesis of a small library of chiral oxindoles and showed nearly identical activity to the homogeneous counterpart except for a modest drop in enantioselectivity (ca. 3–8% *ee*). Notably, the process proved to be easily scalable on the gram-scale and showed highly efficient catalyst recyclability, with retention of satisfactory levels of enantioselectivity with only a slight loss of conversion efficiency. To the best of our knowledge, this work represents the first study that encompasses the use of chiral monoamidine catalysts in the asymmetric synthesis of oxindoles. Indeed, the sole reported use of this class of catalysts is limited to the stereoselective synthesis of nonsymmetric *cis*-stilbene Nutlins precursors^62^ and to the enantioselective reduction of nitroalkenes.^64^ We postulate that the work reported herein might widen the application of this intriguing class of chiral organocatalysts to a broad collection of challenging asymmetric organic transformations. Moreover, it might highlight the strong potential of chiral supported organocatalysts in reaction intensification, increasing the process efficiency and sustainability and paving the way to possible future studies on the transition from conventional batch to continuous flow processes.

## Supplementary Material



## Data Availability

The data underlying this study are available in the published article and its Supporting Information.

## Notes

General experimental conditions, procedures for the synthesis and characterization of isatins **S 1d–g**, *N*-Boc ketimines **1**, and aza-Henry products **3**; NMR spectra of isatins **S 1d–g**, *N*-Boc ketimines **1**, and aza-Henry products **3**; HPLC chromatograms of aza-Henry products **3** (PDF)
