# Supplementary material for: Chiral Monoamidines as Effective Organocatalysts for the Stereoselective Synthesis of Oxindoles under Homogeneous and Heterogeneous Conditions
Source: J Org Chem. 2025 Nov 24;90(48):16964–72. doi: 10.1021/acs.joc.5c01894 (PMC12690594; doi:10.1021/acs.joc.5c01894)
Supplement: Supplementary file 1 [file jo5c01894_si_001.pdf]

Supporting Information

Chiral Monoamidines as Effective Organocatalysts for the Stereoselective Synthesis of Oxindoles under Homogeneous and Heterogeneous Conditions

Sofia Toldo,[a] Lorenzo Poletti,[a] Graziano Di Carmine,[a] Carmela De Risi,[b] Alessandro Massi,[b] and Daniele Ragno[*b]*

[a]Department of Environmental and Prevention Sciences, University of Ferrara, Via L. Borsari, 46 – 44121 Ferrara (Italy)
[b]Department of Chemical, Pharmaceutical and Agricultural Sciences, University of Ferrara, Via L. Borsari, 46 – 44121 Ferrara (Italy)

daniele.ragno@unife.it

Table of Content

General experimental conditions *S3*

Synthesis of Starting Compounds *S4*

General Procedure for the Synthesis of Isatins S1d-g S4

General Procedure for the Synthesis of *N*-Boc ketimines 1 ..... S6

Synthesis of Products *S8*

General Procedure for the Synthesis of Chiral Oxindoles 3 Under Homogeneous Conditions S8

General Procedure for the Synthesis of Racemic Oxindoles 3 ..... S8

Table S1. Reaction optimization using nitromethane S9

Procedure for the Synthesis of Chiral Oxindole using Nitrometane Under Homogeneous Conditions S9

Procedure for the Synthesis of Racemic Oxindole using Nitrometane S9

General Procedure for the Synthesis of Chiral Oxindoles 3 Under Heterogeneous Conditions and Catalyst Recycle S10

Procedure for the Synthesis of Chiral Oxindole 3ac on gram-scale S10

References *S19*

NMRs of Starting Compounds *S21*

NMRs of Isatins S1d-g S21

NMRs of *N*-Boc ketimines 1a-g S25

NMRs of Oxindoles 3 ..... *S32*

HPLC Chromatograms of Oxindoles 3 ..... *S47*

General experimental conditions

All moisture-sensitive reactions were performed using oven-dried glassware under an argon atmosphere. Anhydrous solvents were freshly distilled and dried over a standard drying agent prior to use. Aryl nitromethanes **2**,[1] homogeneous catalyst **MAM**,[2] heterogeneous catalysts **PS-(*S*)-Pyr-MAM**,[1] **PS-(*R*)-Pyr-MAM**[1] were prepared according to literature procedures Isatins **S1a-c** were commercially available. Reactions were monitored by TLC on silica gel 60 F254 with detection with phosphomolybdic acid. Flash chromatography was performed on silica gel 60 (230-400 mesh). ^{1}H, ^{13}C NMR spectra were recorded on Varian Mercury Plus 400 and Brucker Magnet System Ascend 500 MHz in $CDCl_3$ at room temperature and in DMSO-d_6 at 120 °C for compound **3ah**. ^{13}C{^{1}H} NMR spectra were recorded in ^{1}H broad-band decoupled mode and chemical shifts (δ) are reported in parts per million (ppm) relative to the residual solvent peak. For high resolution mass spectrometry (HRMS) the compounds were analyzed in positive ion mode using an Agilent 6520 HPLC-Chip Q/TOF-MS (nanospray) with a quadrupole, a hexapole, and a time of flight unit to produce the spectra. The capillary source voltage was set at 1700 V; the gas temperature and drying gas were kept at 350 °C and 5 L min^{-1}, respectively. The MS analyzer was externally calibrated with ESI-L low concentration tuning mix from m/z 118 to 2700 to yield accuracy below 5 ppm. Accurate mass data were collected by directly infusing samples in 40/60 H_2O/ACN 0.1% TFA into the system at a flow rate of 0.4 μL min^{-1}. Elemental analyses were performed using a FLASH 2000 series CHNS/O analyzer (ThermoFisher Scientific). Enantiomeric excess (*ee*) and diastereomeric ratio (dr) were evaluated by HPLC using Chiralpak IA column. Optical rotations were measured at 25 ± 2 °C in the stated solvent; $[\alpha]^{D}$ values are given in 10^{-1} deg cm^{2} g^{-1} (concentration *c* given as g/100mL). Absolute stereochemistry for aza-Henry products **3** was determined by comparison of the measured optical rotation value and HPLC chromatogram with the corresponding literature data.[3]

Synthesis of Starting Compounds

General Procedure for the Synthesis of Isatins S1d-g (slightly modified conditions from lit.[4])

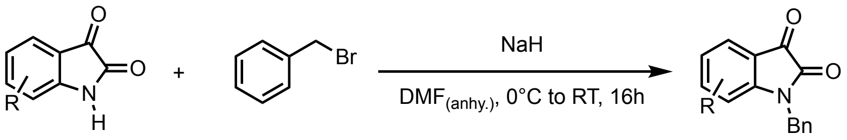

Inside a 50 mL two-neck round bottom flask under argon atmosphere, isatin (1 equiv., 5.50 mmol); sodium hydride (60% in mineral oil, 1.2 equiv., 6.60 mmol) previously washed with hexane, and 20 mL of anhydrous DMF were added. The resulting mixture was stirred at 0°C for 15 minutes and then benzylbromide (1.1 equiv., 6.10 mmol) was added over 30 min. The resulting reaction was brought to RT and stirred for 16 hours. The mixture was then quenched with 10 mL of NH_4Cl (satd. solution) and extracted with EtOAc (3 x 30 mL). The gathered organic phases were dried, and the solvent removed under reduced pressure using rotary evaporator. The crude was purified by flash chromatography on silica gel (cyclohexane/EtOAc) to get the desired Isatin.

1-benzyl-5-chloroindoline-2,3-dione **S1d**[5]

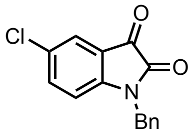

Prepared according to the general procedure reported above using 5-chloroindoline-2,3-dione (1 equiv., 5.50 mmol; 1 g); and Benzyl bromide (1.1 equiv., 6.10 mmol; 725 µL). The reaction mixture was purified by flash chromatography (cyclohexane/EtOAc 7:3) to afford S**1d** as an orange foam (46%, 2.53 mmol, 685 mg). ^{1}H NMR (400 MHz, $CDCl_3$) δ= 7.58 (d, *J* = 2.2 Hz, 1H), 7.43 (dd, *J* = 8.4, 2.2 Hz, 1H), 7.39 – 7.28 (m, 5H), 6.72 (d, *J* = 8.4 Hz, 1H), 4.93 (s, 2H). ^{13}C{^{1}H} NMR (101 MHz, $CDCl_3$) δ= 166.3, 157.7, 148.9, 137.6, 134.0, 129.8, 129.2 (2C), 128.3, 127.4 (2C), 125.3, 118.5, 112.3, 44.2. HRMS (ESI): calcd. for $C_{15}H_{11}ClNO_2$ $[M+H]^+$: 272.0473 found 272.0466.

1-benzyl-5-methoxyindoline-2,3-dione **S1e**[5]

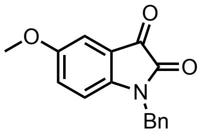

Prepared according to the general procedure using 5-methoxyindoline-2,3-dione (1 equiv., 5,50 mmol; 970 mg); and Benzyl bromide (1.1 equiv., 6.21 mmol; 725 µL). The reaction mixture was purified by flash chromatography (cyclohexane/EtOAc 7:3) to afford S**1e** as a purple foam (55%, 3.02 mmol, 806 mg). ^{1}H NMR (400 MHz, $CDCl_3$) δ= δ 7.39 – 7.28 (m, 5H), 7.15 (d, *J* = 2.7 Hz, 1H), 7.02 (dd, *J* = 8.6, 2.7 Hz, 1H), 6.67 (d, *J* = 8.6 Hz, 1H), 4.90 (s, 2H), 3.77 (s, 3H).^{13}C{^{1}H} NMR (101 MHz, $CDCl_3$) δ= 183.6, 158.3, 156.5, 144.6, 134.6, 129.0 (2C), 128.1, 127.4 (2C), 124.7, 118.2, 112.0, 109.5, 55.9, 44.1; HRMS (ESI): calcd.for $C_{16}H_{14}NO_3$ $[M+H]^+$: 268.0968 found 268.0966.

1-benzyl-6-chloroindoline-2,3-dione **S1f**[6]

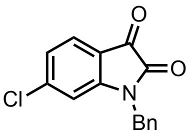

Prepared according to the general procedure using 6-chloroindoline-2,3-dione (1 equiv., 5.50 mmol; 998 mg); and Benzyl bromide (1.1 equiv., 6.21 mmol, 725 µL). The reaction mixture was purified by flash chromatography (cyclohexane/EtOAc 7:3) to afford S**1f** as an orange foam (48%, 2.64 mmol, 715 mg). ^{1}H NMR (400 MHz, $CDCl_3$) δ= 7.55 (d, J = 8.0 Hz, 1H), 7.44 – 7.28 (m, 5H), 7.07 (dd, J = 8.0, 1.7 Hz, 1H), 6.78 (d, J = 1.7 Hz, 1H), 4.91 (s, 2H); ^{13}C{^{1}H} NMR (101 MHz, $CDCl_3$) δ= 181.7, 158.2, 151.7, 144.7, 134.0, 129.2 (2C), 128.4, 127.4 (2C), 126.4, 124.1, 116.0, 111.7, 44.2; HRMS (ESI): calcd. for $C_{15}H_{11}ClNO_2$ $[M+H]^+$: 272.0473 found 272.0475.

1-benzyl-6-methoxyindoline-2,3-dione **S1g**[7]

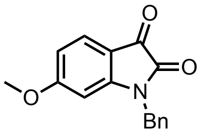

Prepared according to the general procedure using 6-methoxyindoline-2,3-dione (1 equiv., 5.50 mmol; 973 mg); and Benzyl bromide (1.1 equiv., 6.21 mmol; 725 µL). The reaction mixture was purified by flash chromatography (cyclohexane/EtOAc 7:3) to afford S**1g** as an orange foam (47%, 2.58 mmol, 689 mg). ^{1}H NMR (400 MHz, $CDCl_3$) δ= 7.59 (d, J = 8.4 Hz, 1H), 7.38 – 7.28 (m, 5H), 6.52 (dd, J = 8.4, 2.1 Hz, 1H), 6.25 (d, J = 2.1 Hz, 1H), 4.90 (s, 2H), 3.82 (s, 3H).^{13}C {^{1}H} NMR (101 MHz, $CDCl_3$) δ= 180.5, 168.1, 159.7, 153.2, 134.7, 129.0 (2C), 128.1, 128.0, 127.4 (2C), 111.4, 107.9, 98.3, 56.0, 44.0. ; HRMS (ESI): calcd. for $C_{16}H_{14}NO_3$ $[M+H]^+$: 268.0968 found 268.0975.

General Procedure for the Synthesis of *N*-Boc ketimines 1[8]

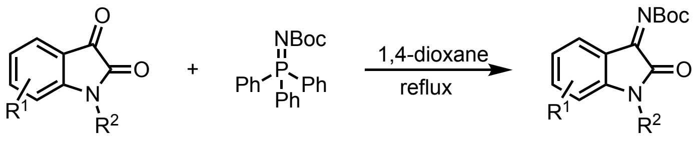

In an oven–dried Schlenk flask under argon atmosphere, Isatin (1 equiv., 2.01 mmol) and tert-butyl (triphenyl- λ^5-phosphaneylidene)carbamate (1.1 equiv., 2.32 mmol) were added sequentially. After an injection of anhydrous 1,4-dioxane (10 mL), the mixture was placed in an oil bath and heated under reflux until the complete disappearance of the starting materials. The reaction was then cooled to room temperature, the solvent removed using a rotary evaporator and the crude purified by flash chromatography (silica gel, cyclohexane/ethyl acetate) to afford the resulting ketimine.

<u>*tert*-butyl (*E*)-(1-benzyl-2-oxoindolin-3-ylidene)carbamate **1a**[8]</u>

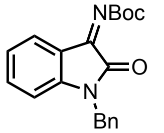

1a

By following the procedure reported above, using 1-benzylindoline-2,3-dione (1 equiv., 2.01 mmol, 550 mg), *N*-Boc ketimine **1a** (80%, 1.61 mmol, 540 mg) was obtained as an amorphous yellow/orange solid after purification by flash chromatography on silica gel using cyclohexane/EtOAc 7:3. ^{1}H NMR (400 MHz, $CDCl_3$): δ= 7.65 (d, *J* = 5.0 Hz, 1H), 7.40 – 7.28 (m, 6H), 7.05 (t, *J* = 7.1 Hz, 1H), 6.71 (d, *J* = 7.9 Hz, 1H), 4.90 (s, 2H), 1.64 (s, 9H). ^{13}C{^{1}H} NMR (101 MHz, $CDCl_3$) δ=160.5, 153.1, 147.3, 135.3, 134.6, 128.9, 128.0 (2C), 127.4 (3C), 124.4, 123.5 (2C), 110.3, 83.6, 44.0, 28.0 (3C); IR (KBr): 3406, 2979, 1736, 1613, 1470, 1358, 1255, 1151, 1099, 752 cm^{-1}; HRMS calcd. for $C_{20}H_{21}N_2O_3$ $[M+H]^+$: 337.1547, found 337.1559.

<u>*tert*-butyl (*E*)-(2-oxoindolin-3-ylidene)carbamate **1b**[8]</u>

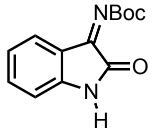

1b

By following the procedure reported above, using 5-methoxyindoline-2,3-dione (1 equiv., 2.01 mmol, 356 mg), *N*-Boc ketimine **1b** (50%, 1.00 mmol, 246 mg) was obtained as an amorphous yellow solid after purification by flash chromatography on silica gel using cyclohexane/EtOAc 7:3. ^{1}H NMR (400 MHz, $CDCl_3$) δ= 7.99 (s, 1H), 7.64 (d, *J* = 6.7 Hz, 1H), 7.44 (t, *J* = 7.8, 1H), 7.08 (t, *J* = 7.8 Hz, 1H), 6.86 (d, *J* = 7.8 Hz, 1H), 1.62 (s, 9H); ^{13}C{^{1}H} NMR (101 MHz, $CDCl_3$) δ= 160.4, 158.1, 145.5, 135.5 (2C), 124.8, 123.6, 119.9, 111.2, 83.5, 28.0 (3C); HRMS (ESI): calcd. for $C_{13}H_{15}N_2O_3$ $[M+H]^+$: 247.1077 found 247.1074.

<u>*tert*-butyl (*E*)-(1-methyl-2-oxoindolin-3-ylidene)carbamate **1c**[8]</u>

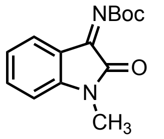

1c

By following the procedure reported above, using 1-methylindoline-2,3-dione (1 equiv., 2.01 mmol, 324 mg), *N*-Boc ketimine **1c** (81%, 1.10 mmol, 286 mg) was obtained as an amorphous red foam after purification by flash chromatography on silica gel using cyclohexane/EtOAc 7:3. ^{1}H NMR (400 MHz, $CDCl_3$) δ= 7.62 (d, *J* = 7.5 Hz, 1H), 7.48 (t, *J* = 7.8 Hz, 1H), 7.08 (t, *J* = 7.5 Hz, 1H), 6.83 (d, *J* = 7.8 Hz, 1H), 3.20 (s, 3H), 1.61 (s, 9H). ^{13}C{^{1}H} NMR (101 MHz, $CDCl_3$) 160.4, 157.3, 153.1, 148.1, 135.3, 124.3, 123.5, 119.3, 109.2, 83.4, 28.0 (3C), 26.1. HRMS (ESI): calcd. for $C_{14}H_{17}N_2O_3$ $[M+H]^+$: 261.1234 found 261.1238.

<u>*tert*-butyl (*E*)-(5-methoxy-1-methyl-2-oxoindolin-3-ylidene)carbamate **1d**</u>[9]

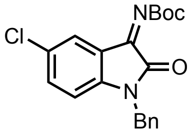

1d

By following the procedure reported above, using Isatin **S1d** (1 equiv., 2.01 mmol, 545 mg), *tert*-butyl (*E*)-(5-chloro-1-methyl-2-oxoindolin-3-ylidene)carbamate **1d** (46%, 0.92 mmol, 342 mg) was obtained as an amorphous orange foam after purification by flash chromatography on silica gel using cyclohexane/EtOAc 7:3. ^{1}H NMR (400 MHz, $CDCl_3$) δ 7.63 (s, 1H), 7.38 – 7.26 (m, 6H), 6.64 (d, *J* = 8.3 Hz, 1H), 4.89 (s, 2H), 1.64 (s, 9H).^{13}C {^{1}H} NMR (101 MHz, $CDCl_3$) δ= 159.9, 156.9, 145.6, 134.7, 134.2, 129.1 (2C), 128.2, 127.3 (2C), 124.4, 120.8, 111.5, 84.0, 44.1, 28.0 (3C); HRMS (ESI): calcd. for $C_{20}H_{20}ClN_2O_3$ $[M+H]^+$: 371.1157 found 371.1161.

<u>*tert*-butyl (*E*)-(1-benzyl-5-methoxy-2-oxoindolin-3-ylidene)carbamate **1e**</u>[9]

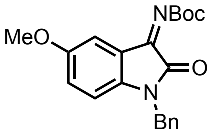

1e

By following the procedure reported above using Isatin **S1e** (1 equiv., 2.01 mmol, 537 mg), *N*-Boc ketimine **1e** (52%, 1.04 mmol, 387 mg) was obtained as an amorphous red foam after purification by flash chromatography on silica gel using cyclohexane/EtOAc 7:3. ^{1}H NMR (400 MHz, $CDCl_3$) δ= 7.37 – 7.27 (m, 5H), 7.22 (s, 1H), 6.91 (dd, *J* = 8.6, 2.7 Hz, 1H), 6.60 (d, *J* = 8.6 Hz, 1H), 4.87 (s, 2H), 3.75 (s, 3H), 1.65 (s, 9H).; ^{13}C{^{1}H} NMR (101 MHz, $CDCl_3$) δ= 160.4; 156.3; 153.5; 141.1; 134.7; 128.9; 128.0 (2C); 127.4 (2C); 121.6; 120.1; 111.2; 109.0; 83.6; 55.9; 44.0; 28.0 (3C); HRMS (ESI): calcd. for $C_{21}H_{23}N_2O_4$ $[M+H]^+$: 367.1652 found 367.1644.

<u>*tert*-butyl (*E*)-(1-benzyl-6-chloro-2-oxoindolin-3-ylidene)carbamate **1f**</u>[9]

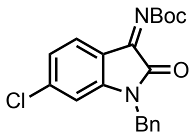

1f

By following the procedure reported above using Isatin **S1f** (1 equiv., 2.01 mmol, 545 mg), *N*-Boc ketimine **1f** (44%, 0.88 mmol, 326 mg) was obtained as an amorphous red foam after purification by flash chromatography on silica gel using cyclohexane/EtOAc 7:3. ^{1}H NMR (400 MHz, $CDCl_3$) δ= 7.58 (d, *J* = 8.0 Hz, 1H), 7.42 – 7.25 (m, 5H), 7.05 (d, *J* = 8.0 Hz, 1H), 6.72 (s, 1H), 4.87 (s, 2H), 1.64 (s, 9H). ^{13}C {^{1}H} NMR (400 MHz, $CDCl_3$) δ= 162.5, 160.2, 148.3, 141.4, 134.1, 129.1 (2C), 128.3, 127.3 (2C), 125.3, 123.8 (2C), 117.8, 111.0, 83.9, 44.1, 28.0 (3C); HRMS (ESI): calcd. for $C_{20}H_{20}ClN_2O_3$ $[M+H]^+$: 371.1157 found 371.1148.

<u>*tert*-butyl (*E*)-(1-benzyl-6-methoxy-2-oxoindolin-3-ylidene)carbamate **1g**</u>

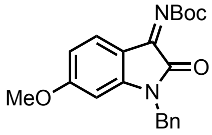

1g

By following the procedure reported above using Isatin **S1g** (1 equiv., 2.01 mmol, 537 mg), *N*-Boc ketimine **1g** (48%, 0.96 mmol, 351 mg) was obtained as an amorphous reddish foam after purification by flash chromatography on silica gel using cyclohexane/EtOAc 7:3. ^{1}H NMR (400 MHz, $CDCl_3$) δ= 7.59 (d, *J* = 7.8 Hz, 1H), 7.39 – 7.28 (m, 5H), 6.52 (d, *J* = 7.8 Hz, 1H), 6.23 (d, *J* = 2.0 Hz, 1H), 4.87 (s, 2H), 3.78 (s, 3H), 1.64 (s, 9H); ^{13}C{^{1}H} NMR (400 MHz, $CDCl_3$) δ= 166.0, 165.8, 165.6, 149.4, 134.8, 128.9 (2C), 128.8, 128.0, 127.4 (2C), 126.4, 112.1, 107.5, 98.0, 55.7, 44.0, 28.0 (3C); HRMS (ESI): calcd. for $C_{21}H_{23}N_2O_4$ $[M+H]^+$: 367.1652 found 367.1647.

Synthesis of Products

General Procedure for the Synthesis of Chiral Oxindoles 3 Under Homogeneous Conditions

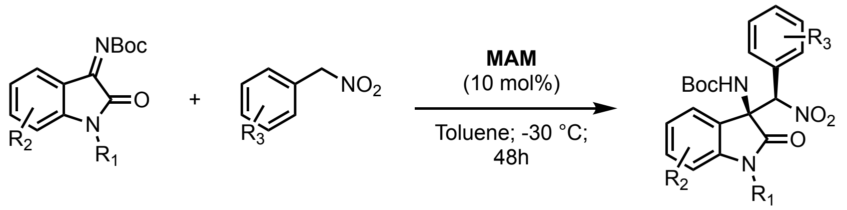

1a-g **2a-g** **3**

Ketimine **1a-g** (1 equiv., 0.20 mmol), Benz-MAM (10 mol %, 10 mg) and aryl nitromethane **2a-g** (1.1 equiv, 0.22 mmol) were placed in a vial. Anhydrous toluene (2 mL) was added and the solution stirred at -30°C for 48 h. The solvent was then removed using rotary evaporator and the crude purified by flash chromatography to afford the desired product.

General Procedure for the Synthesis of Racemic Oxindoles 3

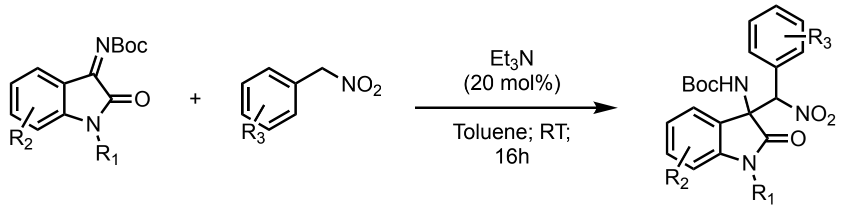

1a-g **2a-g** **3**

Ketimine **1a-g** (1 equiv., 0.20 mmol), Et_3N (20 mol%, 5.5 μL) and aryl nitromethane **2a-g** (1.1 equiv., 0.22 mmol) were placed in a vial. Anhydrous toluene (2 mL) was added and the solution stirred at RT for 16 h. The solvent was then removed using rotary evaporator and the crude purified by flash chromatography to afford the desired product.

Table S1. Reaction optimization using nitromethane[a]

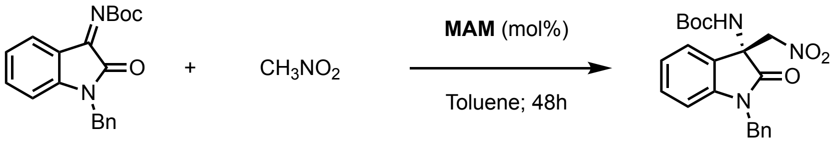

1a 2h 3ah

Entry	Catalyst (mol%)	Temperature (°C)	Yield (%)[b]	*ee* (%)[c]
1	**MAM** (10)	-30	16	54
2[d]	**MAM** (10)	-30	33	60
**3[d]**	**MAM (10)**	**0**	**>95**	**55**
4[d]	**MAM** (10)	25	>95	41
5[d,e]	**MAM** (10)	0	>95	49

[a]**1a** (0.20 mmol), **2h** (0.40 mmol), anhydrous toluene (2.0 mL). [b]Detected by ^{1}H NMR of the crude reaction mixture with acetonitrile as external standard. [c]Enantiomeric excess (*ee*) was determined by chiral HPLC after workup. [d]**2h** (1.8 mmol). [e]CPME as solvent.

Procedure for the Synthesis of Chiral Oxindole using Nitrometane Under Homogeneous Conditions

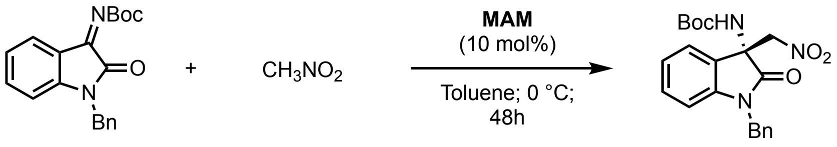

1a 2h 3ah

Ketimine **1a** (1 equiv., 0.20 mmol), Benz-MAM (10 mol %, 10 mg) and nitromethane **2h** (9 equiv, 1.80 mmol) were placed in a vial. Anhydrous toluene (2 mL) was added and the solution stirred at 0°C for 48 h. The solvent was then removed using rotary evaporator and the crude purified by flash chromatography to afford the desired product.

Procedure for the Synthesis of Racemic Oxindole using Nitrometane

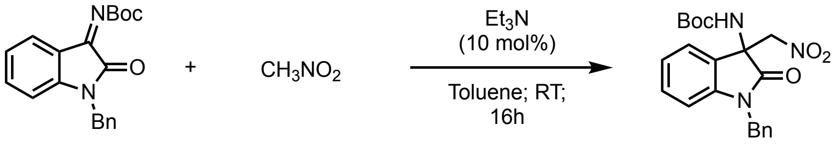

1a 2h 3ah

Ketimine **1a** (1 equiv., 0.20 mmol), Et_3N (10 mol%, 2.7 μL) and nitromethane **2h** (9 equiv., 1.80 mmol) were placed in a vial. Anhydrous toluene (2 mL) was added and the solution stirred at RT for 16 h. The solvent was then removed using rotary evaporator and the crude purified by flash chromatography to afford the desired product.

General Procedure for the Synthesis of Chiral Oxindoles 3 Under Heterogeneous Conditions and Catalyst Recycle

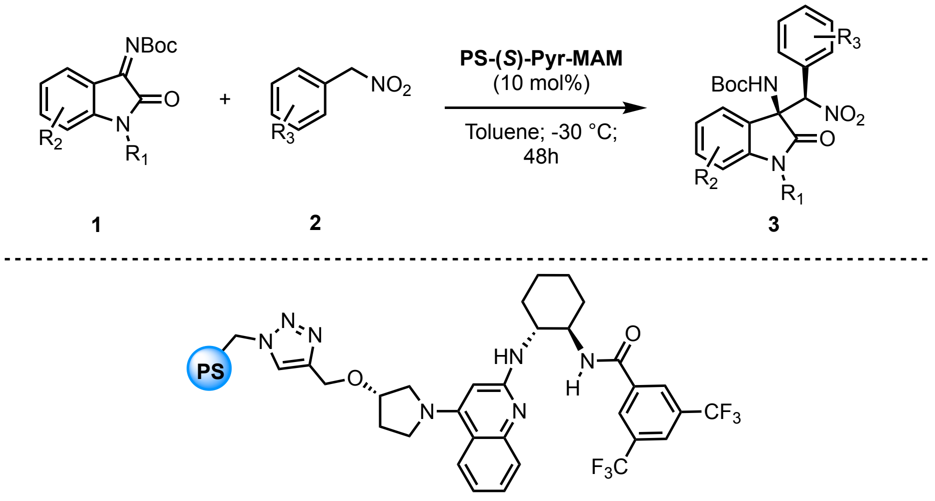

PS-(*S*)-Pyr-MAM (*f*: 0.60 mmol g^{-1})

N-Boc ketimine **1** (1 equiv., 0.20 mmol), PS-(*S*)-Pyr-MAM (10 mol %, *f* = 0.60 mmol g^{-1}, 33 mg) and aryl nitromethane **2** (1.1 equiv., 0.22 mmol) were placed in a vial. Anhydrous toluene (2 mL) was added and the solution stirred at -30°C for 48 h. The mixture was centrifuged, filtered and the solvent removed using rotary evaporator. The crude was then purified by flash chromatography to afford the desired product. The Catalyst was then washed multiple times with Toluene and dried for 8h at 40 °C under high vacuum conditions. The resulting catalyst was then used in the next cycle according to the same procedure.

Procedure for the Synthesis of Chiral Oxindole 3ac on gram-scale

N-Boc ketimine **1a** (1 equiv., 3.00 mmol, 1 g), **PS-(*S*)-Pyr-MAM** (10 mol %, *f* = 0.60 mmol g^{-1}, 500 mg) and aryl nitromethane **2c** (1.1 equiv., 3.30 mmol, 498 mg) were placed in a reaction flask. Anhydrous toluene (30 mL) was added and the solution stirred at -30°C for 48 h. The mixture was centrifuged, filtered and the solvent removed using rotary evaporator. The crude was then purified by flash chromatography (cyclohexane/EtOAc 8:2) to afford oxindole **3ac** as an amorphous white solid (82%, 2.46 mmol, 1.20 g). The major diastereomer was determined to be 88% *ee* with dr=87:13 The Catalyst was then washed multiple times with Toluene and dried for 8h at 40 °C under high vacuum conditions.

<u>*tert*-butyl ((*S*)-1-benzyl-3-((*R*)-(4-chlorophenyl)(nitro)methyl)-2-oxoindolin-3-yl)carbamate **3aa**</u>

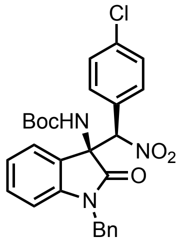

3aa

By following general procedure under homogenous conditions using N-Boc ketimine **1a** (1 equiv., 0.20 mmol, 68 mg) and aryl nitromethane **2a** (1.1 equiv., 0.22 mmol, 38 mg), oxindole **3aa** was obtained as an amorphous white solid (79%, 0.16 mmol, 80 mg) after purification by flash chromatography (cyclohexane/EtOAc 7:3). The major diastereomer was determined to be 92% ee with dr=92:8. By following general procedure under heterogeneous conditions using *N*-Boc ketimine **1a** (1 equiv., 0.20 mmol, 68 mg) and aryl nitromethane **2a** (1.1 equiv., 0.22 mmol, 38 mg), oxindole **3aa** was obtained as an amorphous white solid (79%, 0.16 mmol, 80 mg) after purification by flash chromatography (cyclohexane/EtOAc 7:3). The major diastereomer was determined to be 89% *ee* with dr=95:5.

ee and dr were determined by chiral HPLC (Chiralcel IA: 20% *i*PrOH/hexanes, 1.0 mL min^{-1}, λ = 254 nm): Major (t_{major} = 6.7 min, t_{minor} = 27 min), Minor (t_{major} = 10.9 min, t_{minor} = 17.4.)]. $[\alpha]_D^{20}$ = -23.75 (*c* 0.4, $CHCl_3$); ^{1}H NMR (500 MHz, $CDCl_3$) δ= 7.54 (dd, *J* = 7.5, 0.9 Hz, 1H), 7.31 – 7.27 (m, 1H), 7.25 – 7.19 (m, 3H), 7.17 – 7.10 (m, 3H), 6.94 (d, *J* = 8.6 Hz, 2H), 6.72 (dd, *J* = 6.2, 2.6 Hz, 2H), 6.56 (d, *J* = 7.5 Hz, 1H), 6.06 (s, 1H), 5.96 (s, 1H), 4.92 (d, *J* = 15.8 Hz, 1H), 4.42 (d, *J* = 15.8 Hz, 1H), 1.32 (s, 9H).^{13}C{^{1}H} NMR (101MHz, $CDCl_3$) δ= 172.7, 153.3, 143.9, 137.0, 134.7, 131.3 (2C), 130.5, 128.6 (2C), 128.6 (2C), 127.6, 126.8 (2C), 126.2, 125.4, 124.3, 123.2, 109.6, 92.1, 81.3, 63.8, 44.6, 28.1 (3C).; HRMS (ESI) m/z: $[M + H]^+$ calcd. for $C_{27}H_{27}ClN_3O_5$ 508.1634 found 508.1619.

<u>*tert*-butyl ((*S*)-1-benzyl-3-((*R*)-(4-bromophenyl)(nitro)methyl)-2-oxoindolin-3-yl)carbamate **3ab**</u>

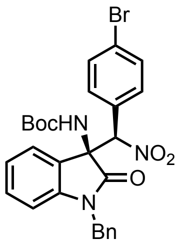

3ab

By following general procedure under homogenous conditions using *N*-Boc ketimine **1a** (1 equiv., 0.20 mmol, 68 mg) and aryl nitromethane **2b** (1.1 equiv., 0.22 mmol, 48 mg), oxindole **3ab** was obtained as an amorphous white solid (91%, 0.18 mmol, 100 mg) after purification by flash chromatography (cyclohexane/EtOAc 8:2). The major diastereomer was determined to be 85% *ee* with 95:5 dr.

ee and dr were determined by chiral HPLC (Chiralcel IA: 20% *i*PrOH/hexanes, 1.0 mL min^{-1}, λ = 254 nm): Major (t_{major} =6.6 min, t_{minor} = 24.6 min), Minor (t_{major} = 10.7 min, t_{minor} = 9.2)]. $[\alpha]_D^{20}$ = -28.3 (*c* 1.6, $CHCl_3$);^{1}H NMR (400 MHz, $CDCl_3$) δ 7.56 (d, *J* = 7.6 Hz, 1H), 7.31 – 7.27 (m, 3H), 7.26 – 7.21 (m, 3H), 7.17 – 7.1 (m, , 1H), 6.88 (d, *J* = 8.6 Hz, 2H), 6.74 – 6.69 (m, 2H), 6.56 (d, *J* = 7.6 Hz, 1H), 6.05 (s, 1H), 5.94 (s, 1H), 4.92 (d, *J* = 15.8 Hz, 1H), 4.42 (d, *J* = 15.8 Hz, 1H), 1.32 (s, 9H).^{13}C{^{1}H} NMR (101 MHz, $CDCl_3$) δ 172.6, 153.3, 143.9, 134.6, 132.4, 131.6 (2C), 131.5 (2C), 130.6, 128.7 (2C), 127.6, 126.8 (2C), 126.7, 125.4, 124.3, 123.2, 109.6, 92.2, 81.3, 63.7, 44.6, 28.1 (3C). HRMS (ESI) m/z: $[M + H]^+$ calcd. for $C_{27}H_{27}BrN_3O_5$ 552.1129 found 552.1136.

<u>*tert*-butyl ((*S*)-1-benzyl-3-((*R*)-nitro(*p*-tolyl)methyl)-2-oxoindolin-3-yl)carbamate **3ac**[3]</u>

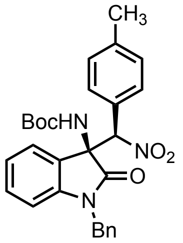

3ac

By following general procedure under homogenous conditions using *N*-Boc ketimine **1a** (1 equiv., 0.20 mmol, 68 mg) and aryl nitromethane **2c** (1.1 equiv., 0.22 mmol, 34 mg), oxindole **3ac** was obtained as an amorphous white solid (88%, 0.18 mmol, 86 mg) after purification by flash chromatography (cyclohexane/EtOAc 8:2). The major diastereomer was determined to be 96% *ee* with dr=99:1. By following general procedure under heterogeneous conditions using *N*-Boc ketimine **1a** (1 equiv., 0.20 mmol, 68 mg) and aryl nitromethane **2c** (1.1 equiv., 0.22 mmol, 34 mg), oxindole **3ac** was obtained as an amorphous white solid (86%, 0.17 mmol, 84 mg) after purification by flash chromatography (cyclohexane/EtOAc 8:2). The major diastereomer was determined to be 90% *ee* with dr=94:6. By following general procedure using recycled heterogeneous catalyst using *N*-Boc ketimine **1a** (1 equiv., 0.20 mmol, 68 mg) and aryl nitromethane **2c** (1.1 equiv., 0.22 mmol, 34 mg), oxindole **3ac** was obtained at the sixth reuse of the catalyst as an amorphous white solid (81%, 0.16 mmol, 79 mg) after purification by flash chromatography (cyclohexane/EtOAc 8:2). The major diastereomer at the sixth reuse of the catalyst was determined to be 84% *ee* with dr=80:20.

ee and dr were determined by chiral HPLC (Chiralcel IA: 20% iPrOH/hexanes, 1.0 mL min^{-1}, λ = 254 nm): Major (t_{major} =6.15 min, t_{minor} = 13.7 min), Minor (t_{major} = 8.9 min, t_{minor} = 15.6.)]. $[\alpha]_D{}^{20}$ = -18.6 (*c* 0.1, $CHCl_3$); ^1H NMR (400 MHz, $CDCl_3$) δ= 7.53 (d, *J* = 7.4 Hz, 1H), 7.26 – 7.21 (m, 1H), 7.14 (ddd, *J* = 11.5, 11.5, 7.4 Hz, 4H), 6.97 (d, *J* = 8.1 Hz, 2H), 6.90 (d, *J* = 8.1 Hz, 2H), 6.71 (d, *J* = 7.3 Hz, 2H), 6.50 (d, *J* = 7.3 Hz, 1H), 6.02 (s, 1H), 5.96 (s, 1H), 4.90 (d, *J* = 16.0 Hz, 1H), 4.42 (d, *J* = 16.0 Hz, 1H), 2.32 (s, 3H), 1.31 (s, 9H).$^{13}C\{^1H\}$ NMR (101MHz, $CDCl_3$) δ= 172.9, 153.4, 143.9, 140.7, 134.9, 130.2, 129.8 (2C), 129.0 (2C), 128.4 (2C), 127.3, 126.9 (2C), 125.4, 124.9, 124.7, 123.0, 109.5, 92.9, 81.1, 63.9, 44.5, 28.1 (3C), 21.4; HRMS (ESI) m/z: $[M + H]^+$ calcd. for $C_{28}H_{30}N_3O_5$ 488.2180 found 488.2167.

tert-butyl ((S)-1-benzyl-3-((R)-(3-chlorophenyl)(nitro)methyl)-2-oxoindolin-3-yl)carbamate **3ad**

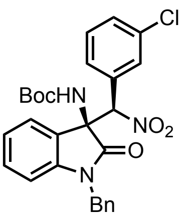
3ad

By following general procedure under homogenous conditions using *N*-Boc ketimine **1a** (1 equiv., 0.20 mmol, 68 mg) and aryl nitromethane **2d** (1.1 equiv., 0.22 mmol, 34 mg), oxindole **3ad** was obtained as an amorphous white solid (90%, 0.18 mmol, 90 mg) after purification by flash chromatography (cyclohexane/EtOAc 8:2). The major diastereomer was determined to be 92% *ee* with dr=93:7.

ee and dr were determined by chiral HPLC (Chiralcel IA: 20% *i*PrOH/hexanes, 1.0 mL min^{-1}, λ = 254 nm): Major (t_{major} =6.6 min, t_{minor} = 15.7 min), Minor (t_{major} = 13.1 min, t_{minor} = 8.9.)]. $[\alpha]_D{}^{20}$ = -2.6 (*c* 0.7, $CHCl_3$); ^{1}H NMR (400 MHz, $CDCl_3$) δ= 7.58 (d, *J* = 7.5 Hz, 1H), 7.45 – 7.27 (m, 3H), 7.22 – 7.07 (m, 4H), 7.03 – 6.95 (m, 2H), 6.84 (d, *J* = 7.5 Hz, 2H), 6.59 (d, *J* = 7.5 Hz, 1H), 6.11 (s, 1H), 5.88 (s, 1H), 4.84 (d, *J* = 15.8 Hz, 1H), 4.50 (d, *J* = 15.8 Hz, 1H), 1.33 (s, 9H).^{13}C{^{1}H} NMR (101MHz, $CDCl_3$) δ 172.5, 153.5, 143.7, 134.8, 134.2, 130.7, 130.6, 130.0, 129.5, 129.4, 128.7 (2C), 128.1, 127.5, 126.9 (2C), 125.5, 124.4, 123.3, 109.6, 91.9, 81.3, 63.5, 44.5, 28.1 (3C); HRMS (ESI) m/z: $[M + H]^+$ calcd. for $C_{27}H_{27}ClN_3O_5$ 508.1634 found 508.1623.

tert-butyl ((S)-1-benzyl-3-((R)-(3-bromophenyl)(nitro)methyl)-2-oxoindolin-3-yl)carbamate **3ae**

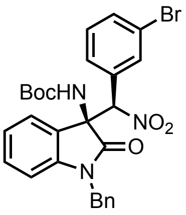
3ae

By following general procedure under homogenous conditions using *N*-Boc ketimine **1a** (1 equiv., 0.20 mmol, 68 mg) and aryl nitromethane **2e** (1.1 equiv., 0.22 mmol, 48 mg), oxindole **3ae** was obtained as an amorphous white solid (87%, 0.17 mmol, 96 mg) after purification by flash chromatography (cyclohexane/EtOAc 8:2). The major diastereomer was determined to be 71% *ee* with dr=94:6.

ee and dr were determined by chiral HPLC (Chiralcel IA: 20% iPrOH/hexanes, 1.0 mL min^{-1}, λ = 254 nm): Major (t_{major} =6.6 min, t_{minor} = 15.3 min), Minor- (t_{major} = 13.5 min, t_{minor} = 12.6.)]. $[\alpha]_D{}^{20}$ = -5.5 (*c* 0.33 , CHCl3); ^{1}H NMR (400 MHz, $CDCl_3$) δ= 7.56 – 7.54 (m, 1H), 7.41 – 7.35 (m, 1H), 7.26 – 7.10 (m, 6H), 7.06 – 7.02 (m, 2H), 6.69 (d, *J* = 7.0 Hz, 2H), 6.49 (d, *J* = 7.8 Hz, 1H), 6.07 (s, 1H), 5.96 (s, 1H), 4.83 (d, *J* = 16.0 Hz, 1H), 4.45 (d, *J* = 16.0 Hz, 1H), 1.32 (s, 9H).^{13}C{^{1}H} NMR (101 MHz, $CDCl_3$) δ= 172.8, 153.4, 143.8, 134.8, 130.5, 130.3, 130.0 (2C), 128.6 (2C), 128.3 (2C), 127.8, 127.3, 126.7 (2C), 125.5, 124.6, 123.0, 109.5, 93.0, 81.1, 63.9, 44.4, 28.1 (3C); HRMS (ESI) m/z: $[M + H]^+$ calcd. for $C_{27}H_{27}BrN_3O_5$ 552.1129 found 552.1143.

tert-butyl ((*S*)-1-benzyl-3-((*R*)-nitro(*m*-tolyl)methyl)-2-oxoindolin-3-yl)carbamate **3af**

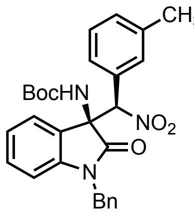

3af

By following general procedure under homogenous conditions using *N*-Boc ketimine **1a** (1 equiv., 0.20 mmol, 68 mg) and aryl nitromethane **2f** (1.1 equiv., 0.22 mmol, 34 mg), oxindole **3af** was obtained as an amorphous white solid (94%, 0.19 mmol, 92 mg) after purification by flash chromatography (cyclohexane/EtOAc 8:2). The major diastereomer was determined to be 76% *ee* with dr=97:3.

ee and dr were determined by chiral HPLC (Chiralcel IA: 20% *i*PrOH/hexanes, 1.0 mL min^{-1}, λ = 254 nm): Major (t_{major} =6.3 min, t_{minor} = 13.9 min), Minor (t_{major} = 18.9 min, t_{minor} = 10.9.)]. $[\alpha]_D^{20}$ = -7.64 (*c* 0.43, $CHCl_3$); ^{1}H NMR (400 MHz, $CDCl_3$) δ= 7.55 (d, *J* = 7.5 Hz, 1H), 7.24 – 7.03 (m, 7H), 6.85 (d, *J* = 7.5 Hz, 1H), 6.78 (s, 1H), 6.65 (d, *J* = 7.5 Hz, 2H), 6.48 (d, *J* = 8.1 Hz, 1H), 6.03 (s, 1H), 5.95 (s, 1H), 4.90 (d, *J* = 15.9 Hz, 1H), 4.42 (d, *J* = 15.9 Hz, 1H), 2.13 (s, 3H), 1.32 (s, 9H). $^{13}C\{^1H\}$ NMR (101 MHz, $CDCl_3$) δ= 172.8, 153.4, 143.9, 138.1, 134.9, 131.2, 130.6, 130.2, 128.6 (2C), 128.2, 127.3, 127.0, 126.7 (2C), 125.5, 123.0, 109.4, 93.0, 81.1, 63.9, 44.4, 28.1 (3C), 21.2; HRMS (ESI): calcd. for $C_{28}H_{30}N_3O_5$ $[M+H]^+$: 488.2180 found 488.2166.

tert-butyl ((*S*)-1-benzyl-3-((*R*)-nitro(phenyl)methyl)-2-oxoindolin-3-yl)carbamate **3ag**[3]

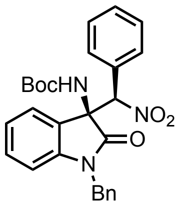

3ag

By following general procedure under homogenous conditions using *N*-Boc ketimine **1a** (1 equiv., 0.20 mmol, 68 mg) and aryl nitromethane**2g** (1.1 equiv., 0.22 mmol, 30 mg), oxindole **3ag** was obtained as an amorphous white solid (91%, 0.18 mmol, 96 mg) after purification by flash chromatography (cyclohexane/EtOAc 8:2). The major diastereomer was determined to be 73% *ee* with dr=93:7.

ee and dr were determined by chiral HPLC (Chiralcel IA: 20% iPrOH/hexanes, 1.0 mL min^{-1}, λ = 254 nm): Major (t_{major} =6.8 min, t_{minor} = 18.2 min), Minor (t_{major} = 11.8 min, t_{minor} = 15.4.)]. $[\alpha]^D$ 20 = -5.1 (*c* 0.14, $CHCl_3$); ^{1}H NMR (400 MHz, $CDCl_3$) δ=7.56 (d, *J* = 7.5 Hz, 1H), 7.46 – 7.29 (m, 2H), 7.25 – 7.09 (m, 6H), 7.05 (d, *J* = 8.0 Hz, 2H), 6.69 (d, *J* = 7.5 Hz, 2H), 6.49 (d, *J* = 7.5 Hz, 1H), 6.08 (s, 1H), 5.99 (s, 1H), 4.84 (d, *J* = 15.9 Hz, 1H), 4.45 (d, *J* = 15.9 Hz, 1H), 1.32 (s, 9H). $^{13}C\{^1H\}$ NMR (400 MHz, $CDCl_3$) δ= 172.9, 153.4, 143.8, 134.8, 130.5, 130.3, 130.0 (2C), 128.6 (2C), 128.3 (2C), 127.8, 127.3, 126.8 (2C), 125.5, 124.6, 123.0, 109.5, 93.0, 81.1, 63.9, 44.4, 28. (3C); HRMS (ESI): calcd. for $C_{27}H_{28}N_3O_5$ $[M+H]^+$: 474.2023 found 474.2025.

tert-butyl ((*S*)-3-((*R*)-(4-chlorophenyl)(nitro)methyl)-2-oxoindolin-3-yl)carbamate **3ba**

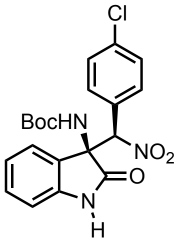

3ba

By following general procedure under homogenous conditions using *N*-Boc ketimine **1b** (1 equiv., 0.20 mmol, 50 mg) and aryl nitromethane **2a** (1.1 equiv., 0.22 mmol, 38 mg), oxindole **3ba** was obtained as an amorphous white solid (84%, 0.17 mmol, 64 mg) after purification by flash chromatography (cyclohexane/EtOAc 7:3). The major diastereomer was determined to be 80% *ee* with dr=72:28.

ee and dr were determined by chiral HPLC (Chiralcel IA: 20% iPrOH/hexanes, 1.0 mL min^{-1}, λ = 254 nm): Major (t_{major} =5.7 min, t_{minor} = 16.9 min), Minor (t_{major} = 10.2 min, t_{minor} = 13.6.)]. $[\alpha]^D$ 20 = -35.7 (*c* 0.5, $CHCl_3$); ^{1}H NMR (400 MHz, $CDCl_3$) δ 7.47 (d, *J* = 7.6 Hz, 1H), 7.41 – 7.36 (m, 1H), 7.34 – 7.29 (m, 1H), 7.21 – 7.16 (m, 2H), 7.07 – 7.03 (m, 1H), 6.96 (d, *J* = 8.4 Hz, 2H), 6.70 (d, *J* = 8.4 Hz, 1H), 6.02 – 5.94 (m, 2H), 1.33 (s, 9H).^{13}C{^{1}H} NMR (101 MHz, $CDCl_3$) δ= 162.1, 151.8, 141.1, 131.4, 131.2 (2C), 130.6, 129.4, 128.4 (2C), 126.1, 125.7, 123.3, 110.1, 91.8, 81.5, 64.0, 28.0 (3C); HRMS (ESI): calcd. for $C_{20}H_{21}ClN_3O_5$ $[M+H]^+$: 418.1164 found 418.1160.

tert-butyl ((*S*)-3-((*R*)-(4-chlorophenyl)(nitro)methyl)-1-methyl-2-oxoindolin-3-yl)carbamate **3ca**[3]

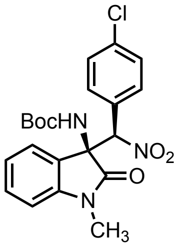

3ca

By following general procedure under homogenous conditions using *N*-Boc ketimine **1c** (1 equiv., 0.20 mmol, 52 mg) and aryl nitromethane **2a** (1.1 equiv., 0.22 mmol, 38 mg), oxindole **3ca** was obtained as an amorphous white solid (93%, 0.19 mmol, 74 mg) after purification by flash chromatography (cyclohexane/EtOAc 7:3). The major diastereomer was determined to be 84% *ee* with dr=92:8.

ee and dr were determined by chiral HPLC (Chiralcel IA: 20% *i*PrOH/hexanes, 1.0 mL min^{-1}, λ = 254 nm): Major (t_{major} =7.4 min, t_{minor} = 18.4 min), Minor (t_{major} = 22.1 min, t_{minor} = 19.1.)]. $[\alpha]_D{}^{20}$ = -5.08 (*c* 1.3, $CHCl_3$); ^{1}H NMR (400 MHz, $CDCl_3$) δ= 7.54 (d, *J* = 7.4 Hz, 1H), 7.47 – 7.31 (m, 1H), 7.21 – 7.10 (m, 3H), 6.95 (d, *J* = 8.5 Hz, 2H), 6.68 (d, *J* = 7.4 Hz, 1H), 6.04 (s, 1H), 5.86 (s, 1H), 2.89 (s, 3H), 1.30 (s, 9H).^{13}C{^{1}H} NMR (101 MHz, $CDCl_3$) δ= 172.2, 153.5, 144.1, 136.7, 130.9 (2C), 130.6, 128.6, 128.2 (2C), 126.9, 125.4, 123.2, 108.4, 91.9, 81.2, 55.4, 28.0 (2C); HRMS (ESI): calcd. for $C_{21}H_{23}ClN_3O_5$ $[M+H]^+$: 432.1321 found 432.1342.

tert-butyl ((*S*)-1-benzyl-5-chloro-3-((*R*)-(4-chlorophenyl)(nitro)methyl)-2-oxoindolin-3-yl)carbamate **3da**

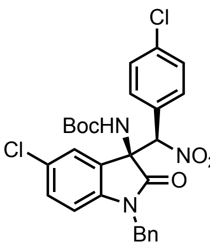

3da

By following general procedure under homogenous conditions using *N*-Boc ketimine **1d** (1 equiv., 0.20 mmol, 74 mg) and aryl nitromethane **2a** (1.1 equiv., 0.22 mmol, 38 mg), oxindole **3ca** was obtained as an amorphous white solid (89%, 0.18 mmol, 74 mg) after purification by flash chromatography (cyclohexane/EtOAc 7:3). The major diastereomer was determined to be 85% *ee* with dr=95:5.

ee and dr were determined by chiral HPLC (Chiralcel IA: 20% *i*PrOH/hexanes, 1.0 mL min^{-1}, λ = 254 nm): Major (t_{major} =6.1 min, t_{minor}= 14.9 min), Minor (t_{major} = 9.7 min, t_{minor} = 16.8.)]. $[\alpha]_D{}^{20}$ = -38.5 (*c* 2.0, $CHCl_3$); ^1H NMR (400 MHz, $CDCl_3$) δ= 7.58 (d, *J* = 2.1 Hz, 1H), 7.25 – 7.13 (m, 6H), 6.98 (d, *J* = 8.6 Hz, 2H), 6.76 – 6.66 (m, 2H), 6.46 (d, *J* = 8.6 Hz, 1H), 6.03 (s, 1H), 5.90 (s, 1H), 4.87 (d, *J* = 15.9 Hz, 1H), 4.43 (d, *J* = 15.9 Hz, 1H), 1.35 (s, 9H).$^{13}C\{^1H\}$ NMR (101 MHz, $CDCl_3$) δ= 172.3, 153.3, 142.5, 137.2, 134.2, 131.1 (2C), 130.5, 128.8 (2C), 128.7 (2C), 127.7, 126.8, 126.0, 126.0, 125.9, 110.6, 92.0, 81.6, 63.8, 44.6, 28.1 (3C); HRMS (ESI): calcd. for $C_{27}H_{26}Cl_2N_3O_5$ $[M+H]^+$: 542.1244 found 542.1248.

tert-butyl ((*S*)-1-benzyl-3-((*R*)-(4-chlorophenyl)(nitro)methyl)-5-methoxy-2-oxoindolin-3-yl)carbamate **3ea**

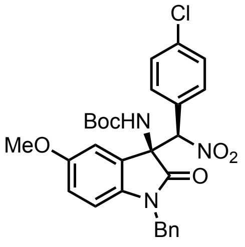

3ea

By following general procedure under homogenous conditions using *N*-Boc ketimine **1e** (1 equiv., 0.20 mmol, 74 mg) and 1aryl nitromethane **2a** (1.1 equiv., 0.22 mmol, 38 mg), oxindole **3ea** was obtained as an amorphous white solid (93%, 0.19 mmol, 100 mg) after purification by flash chromatography (cyclohexane/EtOAc 7:3). The major diastereomer was determined to be 90% *ee* with dr=95:5. By following general procedure under heterogeneous conditions using *N*-Boc ketimine **1e** (1 equiv., 0.20 mmol, 74 mg) and aryl nitromethane **2a** (1.1 equiv., 0.22 mmol, 38 mg), oxindole **3ea** was obtained as an amorphous white solid (87%, 0.17 mmol, 93 mg) after purification by flash chromatography (cyclohexane/EtOAc 7:3). The major diastereomer was determined to be 82% *ee* with dr=96:4.

ee and dr were determined by chiral HPLC (Chiralcel IA: 20% *i*PrOH/hexanes, 1.0 mL min^{-1}, λ = 254 nm): Major (t_{major} =6.4 min, t_{minor}= 27.3 min), Minor (t_{major} = 12.7 min, t_{minor} = 22.7.)]. $[\alpha]_D{}^{20}$ = -36.2 (*c* 2.5, $CHCl_3$); ^1H NMR (400 MHz, $CDCl_3$) δ= ^1H NMR (500 MHz, $CDCl_3$) δ 7.41 (d, *J* = 3.0 Hz, 1H), 7.24 – 7.19 (m, 2H), 7.17 – 7.13 (m, 3H), 6.97 (d, *J* = 8.6 Hz, 2H), 6.79 (dd, *J* = 8.6, 2.6 Hz, 1H), 6.75 – 6.66 (m, 2H), 6.45 (d, *J* = 8.6 Hz, 1H), 6.05 (s, 1H), 5.94 (s, 1H), 4.88 (d, *J* = 15.8 Hz, 1H), 4.39 (d, *J* = 15.8 Hz, 1H), 3.81 (s, 3H), 1.33 (s, 9H).$^{13}C\{^1H\}$ NMR (101 MHz, $CDCl_3$) δ= 172.4, 156.2, 153.4, 137.2, 137.0, 134.7, 131.4 (2C), 128.6 (2C), 128.6 (2C), 127.6, 126.9 (2C), 126.2, 125.5, 115.3, 112.3, 110.2, 92.1, 81.3, 64.1, 55.9, 44.6, 28.1 (3C); HRMS (ESI): calcd.for $C_{28}H_{29}ClN_3O_6$ $[M+H]^+$: 538.1739 found 538.1745.

tert-butyl ((*S*)-1-benzyl-6-chloro-3-((*R*)-(4-chlorophenyl)(nitro)methyl)-2-oxoindolin-3-yl)carbamate **3fa**

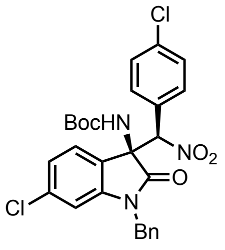
3fa

By following general procedure under homogenous conditions using *N*-Boc ketimine **1f** (1 equiv., 0.20 mmol, 74 mg) and aryl nitromethane **2a** (1.1 equiv., 0.22 mmol, 38 mg), oxindole **3fa** was obtained as an amorphous white solid (95%, 0.19 mmol, 102 mg) after purification by flash chromatography (cyclohexane/EtOAc 7:3). The major diastereomer was determined to be 90% *ee* with dr=97:3. By following general procedure under heterogeneous conditions using *N*-Boc ketimine **1f** (1 equiv., 0.20 mmol, 74 mg) and aryl nitromethane **2a** (1.1 equiv., 0.22 mmol, 38 mg), oxindole **3fa** was obtained as an amorphous white solid (90%, 0.18 mmol, 98 mg) after purification by flash chromatography (cyclohexane/EtOAc 7:3). The major diastereomer was determined to be 85% *ee* with dr=97:3.

ee and dr were determined by chiral HPLC (Chiralcel IA: 20% *i*PrOH/hexanes, 1.0 mL/min, $\lambda = 254$ nm): Major ($t_{major} = 5.3$ min, $t_{minor} = 15$ min), Minor ($t_{major} = 7.5$ min, $t_{minor} = 20.5$.)]. $[\alpha]_D{}^{20} = -20.3$ (*c* 1.05, $CHCl_3$); ^{1}H NMR (400 MHz, $CDCl_3$) δ= 7.48 (d, *J* = 8.1 Hz, 1H), 7.25 – 7.21 (m, 3H), 7.17 (d, *J* = 8.6 Hz, 2H), 7.12 (dd, *J* = 8.6, 1.8 Hz, 1H), 6.98 (d, *J* = 8.6 Hz, 2H), 6.74 – 6.69 (m, 2H), 6.55 (d, *J* = 1.8 Hz, 1H), 6.02 (s, 1H), 5.91 (s, 1H), 4.89 (d, *J* = 15.9 Hz, 1H), 4.40 (d, *J* = 15.9 Hz, 1H), 1.34 (s, 9H). $^{13}C\{^1H\}$ NMR (101 MHz, $CDCl_3$) δ= 172.7, 153.3, 145.1, 137.2, 136.4, 134.1, 131.1 (2C), 128.8 (4C), 127.8, 126.8 (2C), 126.5, 125.9, 123.2, 122.7, 110.2, 91.9, 81.6, 63.5, 44.7, 28.1 (3C); HRMS (ESI): calculated for $C_{27}H_{26}Cl_2N_3O_5$ $[M+H]^+$: 542.1244 found 542.1250.

tert-butyl ((*S*)-1-benzyl-3-((*R*)-(4-chlorophenyl)(nitro)methyl)-6-methoxy-2-oxoindolin-3-yl)carbamate **3ga**

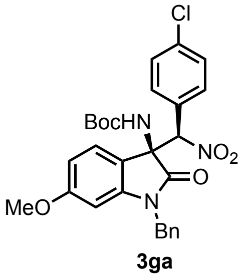
3ga

By following general procedure under homogenous conditions using *N*-Boc ketimine **1g** (1 equiv., 0.20 mmol, 74 mg) and aryl nitromethane **2a** (1.1 equiv., 0.22 mmol, 38 mg), oxindole **3ga** was obtained as an amorphous white solid (85%, 0.17 mmol, 92 mg) after purification by flash chromatography (cyclohexane/EtOAc 7:3). The major diastereomer was determined to be 90% *ee* with dr=87:13. By following general procedure under heterogeneous conditions using *N*-Boc ketimine **1g** (1 equiv., 0.20 mmol, 74 mg) and aryl nitromethane **2a** (1.1 equiv., 0.22 mmol, 38 mg), oxindole **3ga** was obtained as an amorphous white solid (84%, 0.17 mmol, 90 mg) after purification by flash chromatography (cyclohexane/EtOAc 7:3). The major diastereomer was determined to be 86% *ee* with dr=92:8.

ee and dr were determined by chiral HPLC (Chiralcel IA: 20% *i*PrOH/hexanes, 1.0 mL/min, $\lambda = 254$ nm): Major ($t_{major} =7$ min, $t_{minor} = 31.7$ min), Minor ($t_{major} = 10.2$ min, $t_{minor} = 13.6$.)]. $[\alpha]_D{}^{20} = -22.5$ (*c* 1.25, $CHCl_3$); ^{1}H NMR (400 MHz, $CDCl_3$) δ7.44 (d, *J* = 8.5 Hz, 1H), 7.24 – 7.19 (m, 3H), 7.15 (d, *J* = 8.5 Hz, 2H), 6.99 (d, *J* = 8.5 Hz, 2H), 6.74 – 6.69 (m, 2H), 6.61 (dd, *J* = 8.5, 2.3 Hz, 1H), 6.13 (d, *J* = 2.3 Hz, 1H), 6.06 (s, 1H), 5.90 (s, 1H), 4.89 (d, *J* = 15.8 Hz, 1H), 4.39 (d, *J* = 15.8 Hz, 1H), 3.74 (s, 3H), 1.34 (s, 9H). $^{13}C\{^1H\}$ NMR (101 MHz, $CDCl_3$) δ= 173.1, 161.6, 153.4, 145.2, 136.9, 134.6, 131.3 (2C), 129.4, 128.6 (2C), 128.6 (2C), 127.6, 126.9 (2C), 126.4, 116.0, 106.8, 97.7, 92.2, 81.1, 63.5, 55.4, 44.5, 28.1 (3C); HRMS (ESI): calculated for $C_{28}H_{29}ClN_3O_6$ $[M+H]^+$: 538.1739 found 538.1743.

tert-butyl ((*S*)-3-((*R*)-nitro(phenyl)methyl)-2-oxoindolin-3-yl)carbamate **3bg**[3]

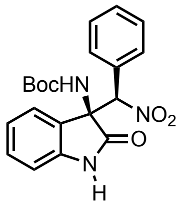

3bg

By following general procedure under homogenous conditions *N*-Boc ketimine **1b** (1 equiv., 0.20 mmol, 50 mg) and aryl nitromethane **2g** (1.1 equiv., 0.22 mmol, 30 mg), oxindole **3bg** was obtained as an amorphous white solid (91%, 0.18 mmol, 70 mg) after purification by flash chromatography (cyclohexane/EtOAc 7:3). The major diastereomer was determined to be 82 % *ee* with dr=95:5.

ee and dr were determined by chiral HPLC (Chiralcel IA: 20% *i*PrOH/hexanes, 1.0 mL/min, λ = 254 nm): Major (t_{major} = 5.9 min, t_{minor} = 11.5 min), Minor (t_{major} = 9.6 min, t_{minor} = 6.4.)]. $[\alpha]_D{}^{20}$ = -10.8 (*c* 1.3, $CHCl_3$); ^{1}H NMR (400 MHz, $CDCl_3$) δ=7.45 (d, *J* = 7.6 Hz, 1H), 7.38 – 7.30 (m, 2H), 7.19 (t, *J* = 7.6 Hz, 2H), 7.11 (td, *J* = 7.6, 0.9 Hz, 1H), 7.05 – 7.00 (m, 2H), 6.66 (d, *J* = 7.6 Hz, 1H), 6.05 (s, 1H), 5.96 (s, 1H), 1.32 (s, 9H); $^{13}C\{^1H\}$ NMR (101 MHz, $CDCl_3$) δ= 173.7, 153.7, 141.3, 130.4, 130.4, 129.8 (2C), 128.2 (2C), 127.7, 125.7, 125.0, 123.0, 110.1, 92.7, 81.4, 64.2, 28.1 (3C); HRMS (ESI): calculated for $C_{20}H_{22}N_3O_5$ $[M+H]^+$: 384.1554 found 384.1560.

tert-butyl (*R*)-(1-benzyl-3-(nitromethyl)-2-oxoindolin-3-yl)carbamate **3ah**[10]

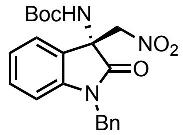

3ah

By following general procedure using nitromethane under homogenous conditions *N*-Boc ketimine **1a** (1 equiv., 0.20 mmol, 50 mg) and nitromethane **2h** (9 equiv., 1.80 mmol, 96 μL), oxindole **3ah** was obtained as an amorphous yellow solid (90%, 0.18 mmol, 71 mg) after purification by flash chromatography (cyclohexane/EtOAc 6:1). The major enantiomer was determined to be 55 % *ee*. *ee* was determined by chiral HPLC (Chiralcel IA: 20% *i*PrOH/hexanes, 1.0 mL/min, λ = 267 nm): Major (t_{major} = 14.8 min), Minor (t_{minor} = 8.3 min). $[\alpha]_D{}^{20}$ = -5.4 (*c* 1.1, $CHCl_3$); ^{1}H NMR (500 MHz, DMSO-d_6, T =120 °C) δ 7.45 (d, *J* = 6.9 Hz, 3H), 7.37 (d, *J* = 7.4 Hz, 1H), 7.35 – 7.30 (m, 2H), 7.27 (d, *J* = 7.4 Hz, 1H), 7.24 (td, *J* = 7.4, 1.3 Hz, 1H), 7.03 (td, *J* = 7.4, 1.3 Hz, 1H), 6.82 (d, *J* = 7.4 Hz, 1H), 5.06 (d, *J* = 13.0 Hz, 1H), 5.03 (d, *J* = 13.0 Hz, 1H), 4.94 (d, *J* = 15.9 Hz, 1H), 4.86 (d, *J* = 15.9 Hz, 1H), 1.27 (s, 9H). $^{13}C\{^1H\}$ NMR (126 MHz, DMSO-d_6, T =120 °C) δ 173.4, 153.4, 143.4, 136.0, 129.7, 128.3 (4C), 127.5, 127.3, 123.3, 122.5, 109.3, 79.4, 77.9, 59.5, 43.5, 27.8 (3C). HRMS (ESI): calculated for $C_{21}H_{24}N_3O_5$ $[M+H]^+$: 398.1710 found 398.1705.

References

(1) Toldo, S.; Poletti, L.; Di Carmine, G.; Bortolini, O.; De Risi, C.; Felletti, S.; Compagnin, G.; Massi, A.; Ragno, D. Effective and Practical Stereoselective Synthesis of Nutlins Precursors by Immobilization of Privileged Chiral Mono-Amidine Catalyst. *Adv. Synth. Catal.* **2025**, *367*, e202500058. https://doi.org/10.1002/adsc.202500058.

(2) Vara, B. A.; Mayasundari, A.; Tellis, J. C.; Danneman, M. W.; Arredondo, V.; Davis, T. A.; Min, J.; Finch, K.; Guy, R. K.; Johnston, J. N. Organocatalytic, Diastereo- and Enantioselective Synthesis of Nonsymmetric Cis -Stilbene Diamines: A Platform for the Preparation of Single-Enantiomer Cis -Imidazolines for Protein-Protein Inhibition. *J. Org. Chem.* **2014**, *79*, 6913–6938. https://doi.org/10.1021/jo501003r.

(3) Hu, Y.; Zhou, Z.; Gong, L.; Meggers, E. Asymmetric Aza-Henry Reaction to Provide Oxindoles with Quaternary Carbon Stereocenter Catalyzed by a Metal-Templated Chiral Brønsted Base. *Org. Chem. Front.* **2015**, *2*, 968–972. https://doi.org/10.1039/c5qo00132c.

(4) Ying, M.; Wang, K.; Yan, W.; Pu, M.; Lin, L. Stable Axially Chiral Cyclohexylidenes from Catalytic Asymmetric Knoevenagel Condensation. *Chemistry – A European Journal* **2024**, *30* (39). https://doi.org/10.1002/chem.202401243.

(5) Lazreg, F.; Lesieur, M.; Samson, A. J.; Cazin, C. S. J. Light-Stable Silver N-Heterocyclic Carbene Catalysts for the Alkynylation of Ketones in Air. *ChemCatChem* **2016**, *8* (1), 209–213. https://doi.org/10.1002/cctc.201500869.

(6) Aikawa, K.; Mimura, S.; Numata, Y.; Mikami, K. Palladium-Catalyzed Enantioselective Ene and Aldol Reactions with Isatins, Keto Esters, and Diketones: Reliable Approach to Chiral Tertiary Alcohols. *European J Org Chem* **2011**, *2011* (1), 62–65. https://doi.org/10.1002/ejoc.201001356.

(7) Lerchner, A.; Carreira, E. M. First Total Synthesis of (±)-Strychnofoline via a Highly Selective Ring-Expansion Reaction. *J Am Chem Soc* **2002**, *124* (50), 14826–14827. https://doi.org/10.1021/ja027906k.

(8) Yan, W.; Wang, D.; Feng, J.; Li, P.; Zhao, D.; Wang, R. Synthesis of *N* -Alkoxycarbonyl Ketimines Derived from Isatins and Their Application in Enantioselective Synthesis of 3-Aminooxindoles. *Org Lett* **2012**, *14* (10), 2512–2515. https://doi.org/10.1021/ol3007953.

(9) Hu, F.-L.; Wei, Y.; Shi, M.; Pindi, S.; Li, G. Asymmetric Catalytic Aza-Morita–Baylis–Hillman Reaction for the Synthesis of 3-Substituted-3-Aminooxindoles with Chiral Quaternary Carbon Centers. *Org Biomol Chem* **2013**, *11* (12), 1921. https://doi.org/10.1039/c3ob27495k.

(10) Fang, B.; Liu, X.; Zhao, J.; Tang, Y.; Lin, L.; Feng, X. Chiral Bifunctional Guanidine-Catalyzed Enantioselective Aza-Henry Reaction of Isatin-Derived Ketimines. *J. Org. Chem.* **2015**, *80*, 6, 3332–3338. https://doi.org/10.1021/acs.joc.5b00075.

NMRs of Starting Compounds

NMRs of Isatins S1d-g

^{1}H-NMR (400 MHz), ^{13}C{^{1}H}-NMR (101 MHz) of 1-benzyl-5-chloroindoline-2,3-dione **S1d** – $CDCl_3$

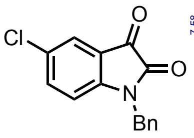

S1d

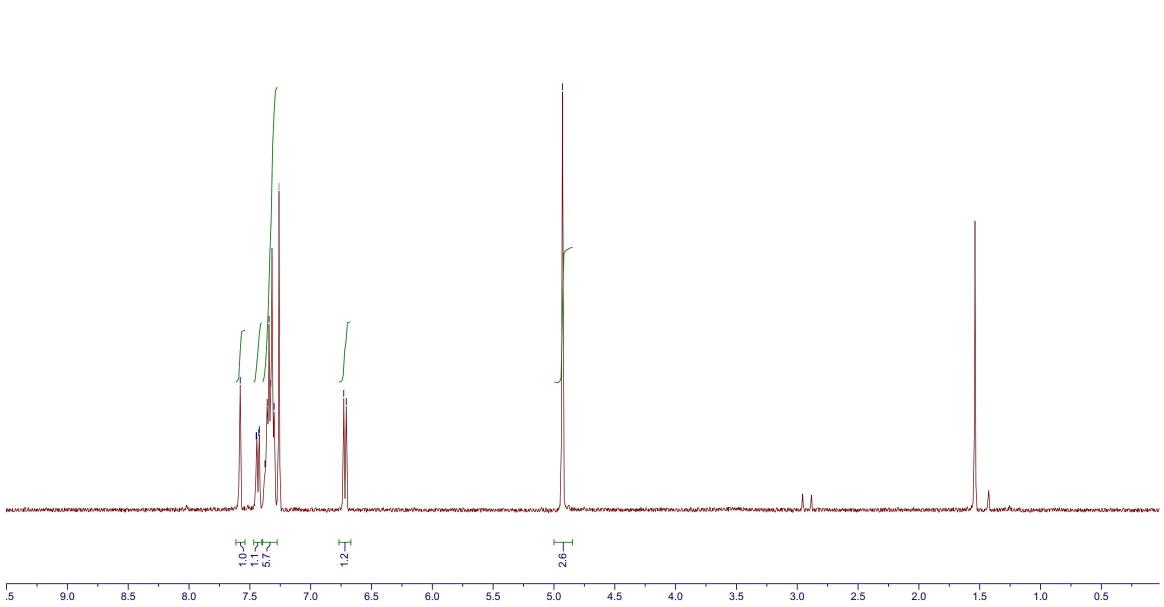

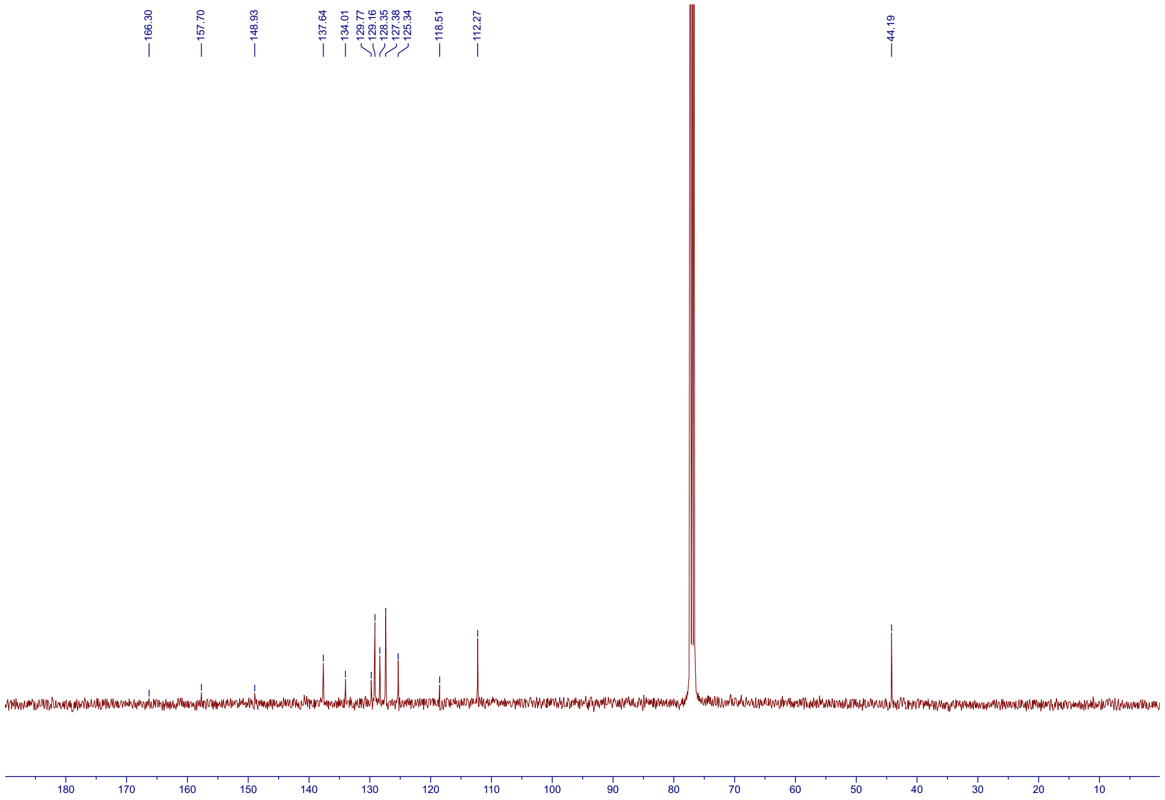

^{1}H-NMR (400 MHz), ^{13}C{^{1}H}-NMR (101 MHz) of 1-benzyl-5-methoxyindoline-2,3-dione **S1e** – $CDCl_3$

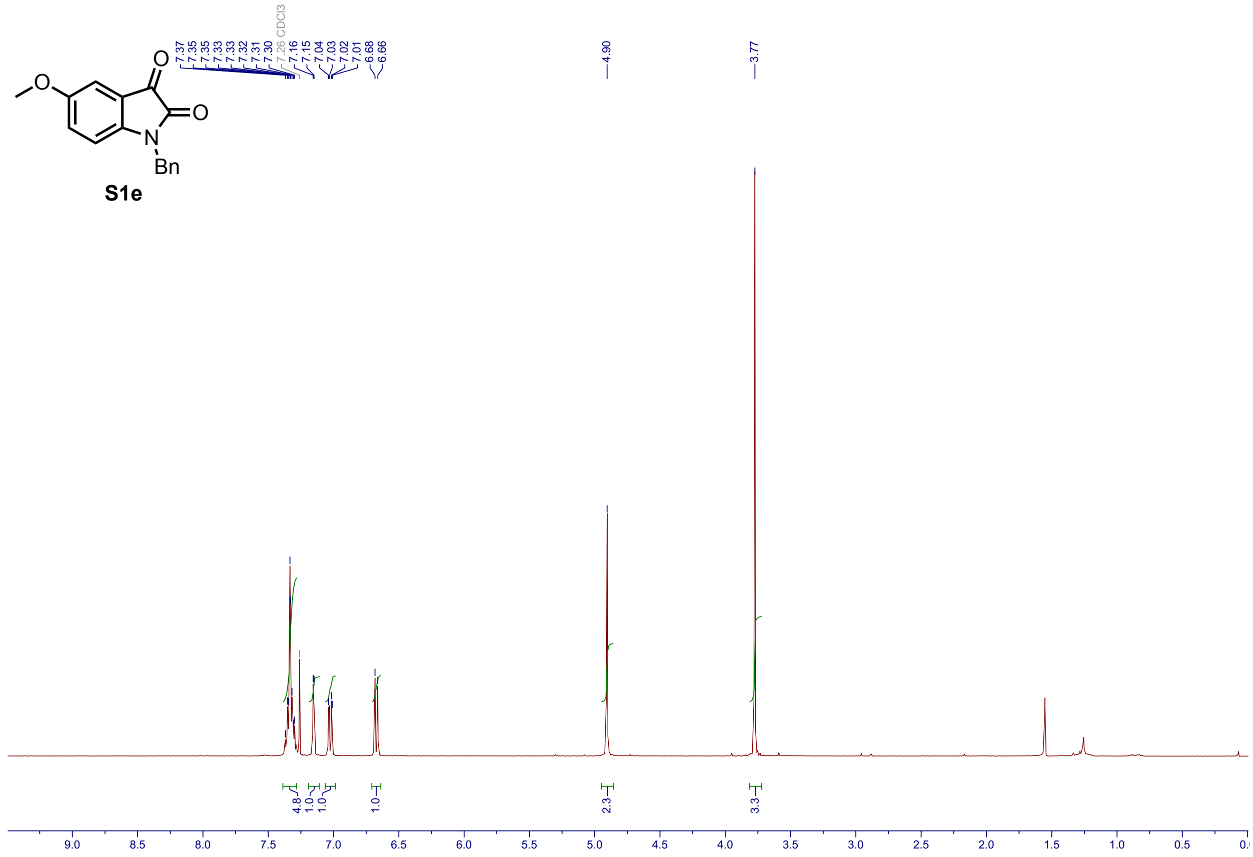

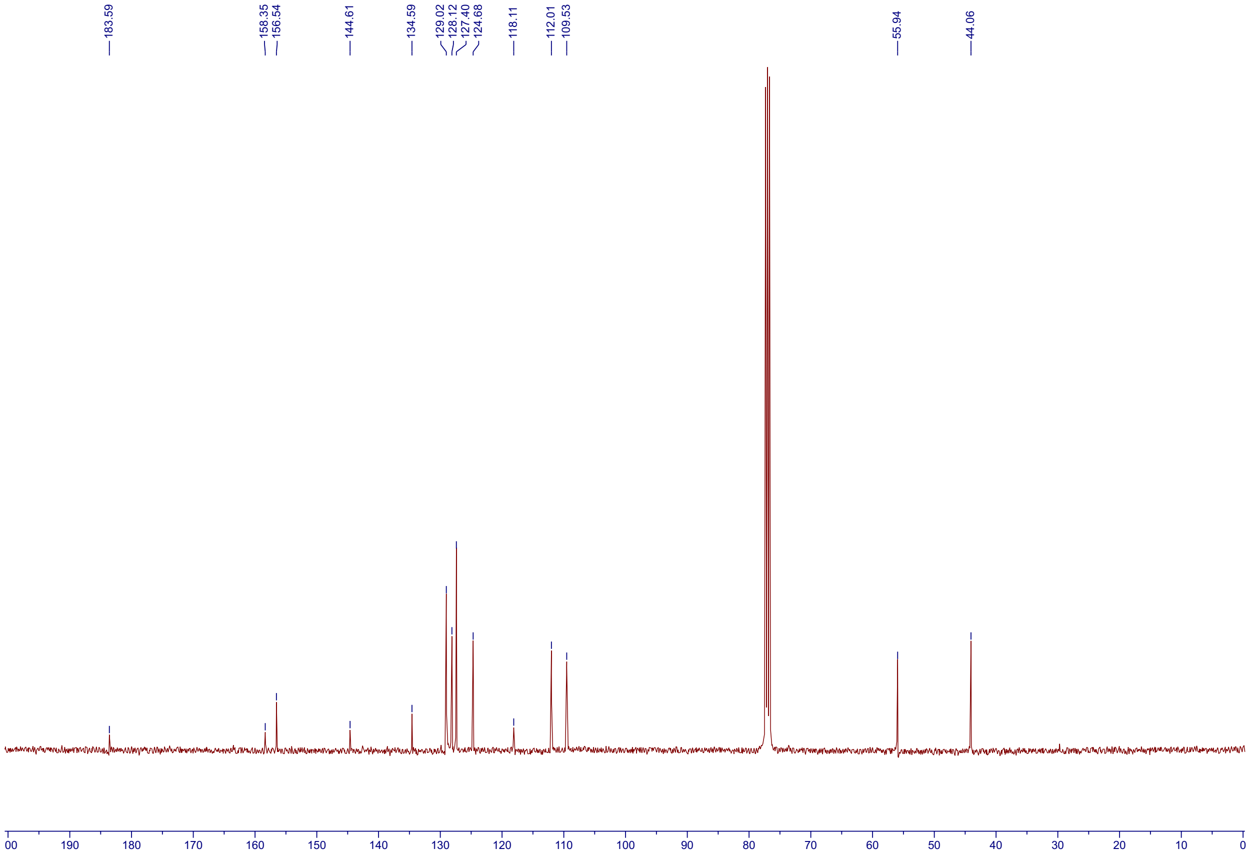

^{1}H-NMR (400 MHz), $^{13}C\{^{1}H\}$-NMR (101 MHz) of 1-benzyl-6-chloroindoline-2,3-dione **S1f** – $CDCl_3$

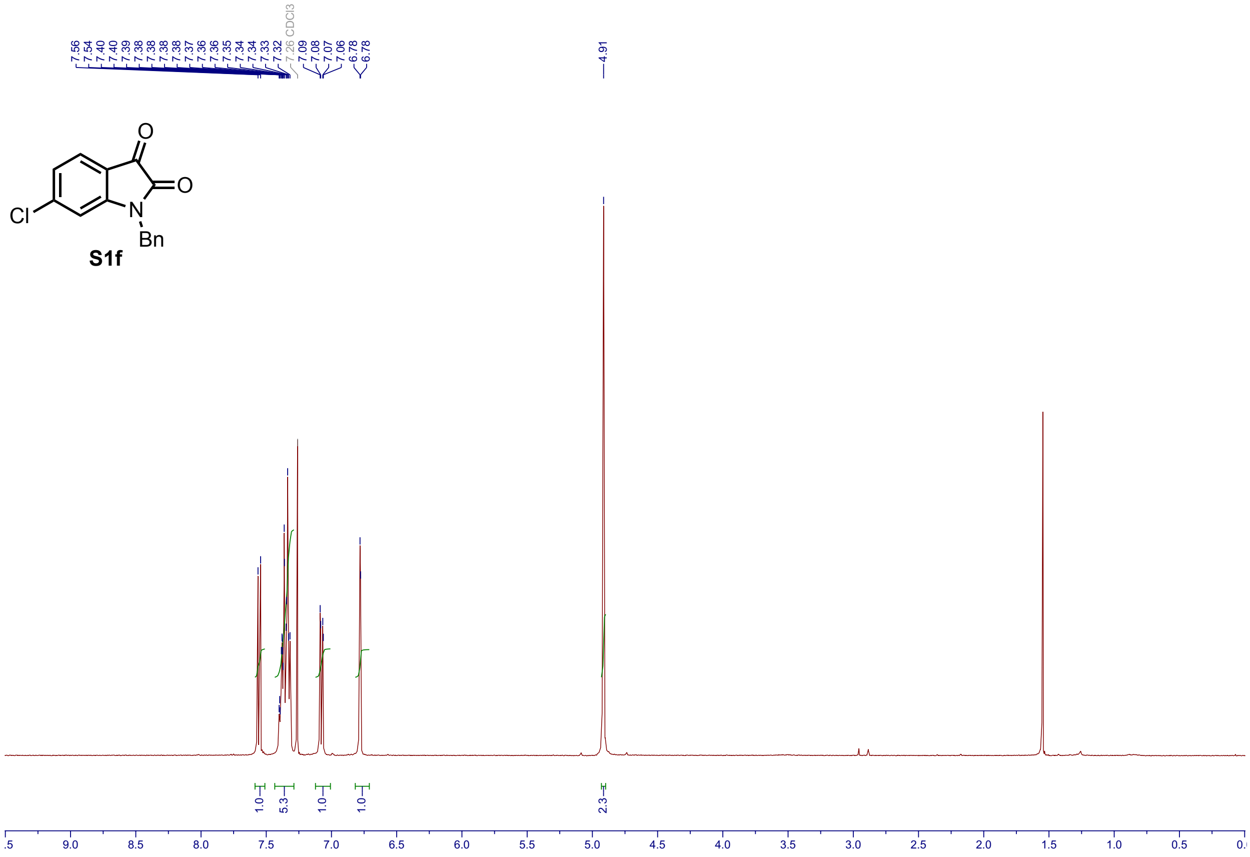

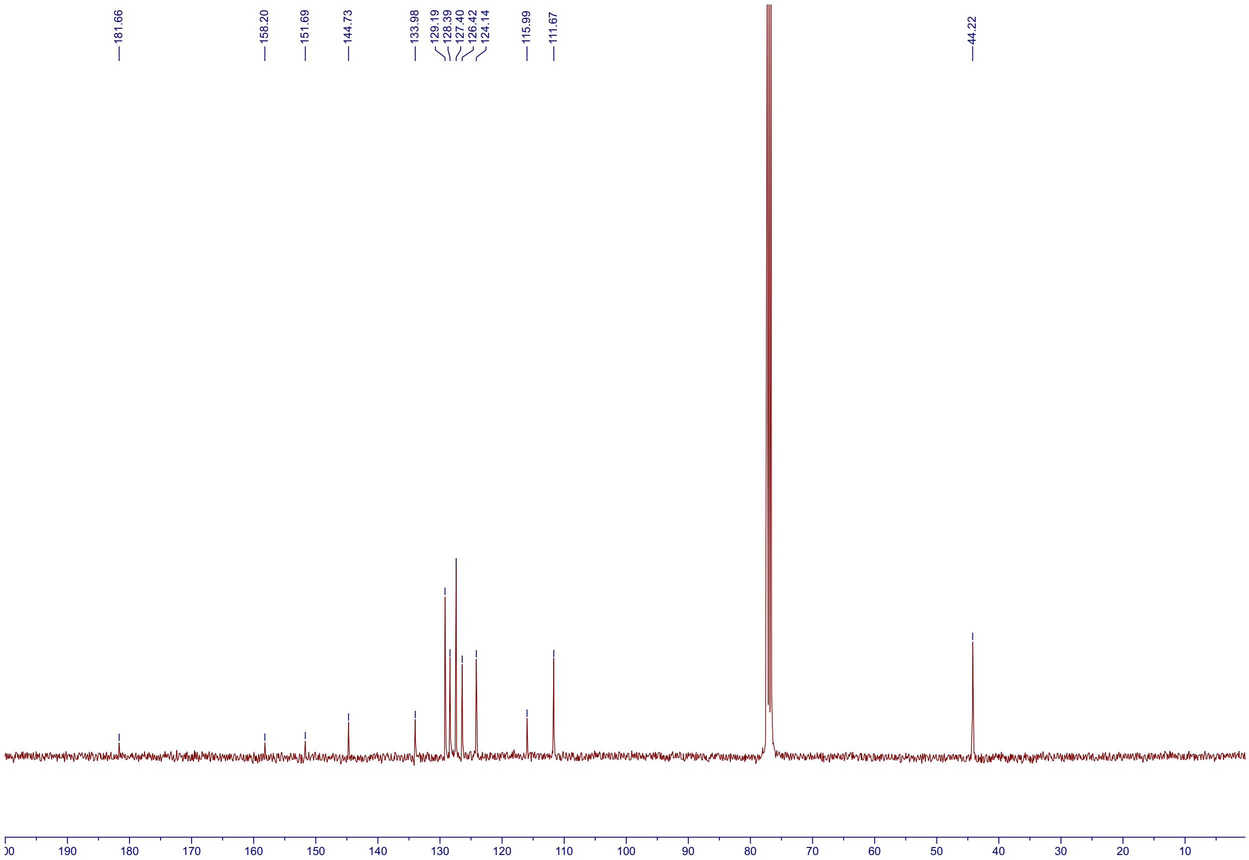

^{1}H-NMR (400 MHz), ^{13}C{^{1}H}-NMR (101 MHz) of 1-benzyl-6-methoxyindoline-2,3-dione **S1g** – $CDCl_3$

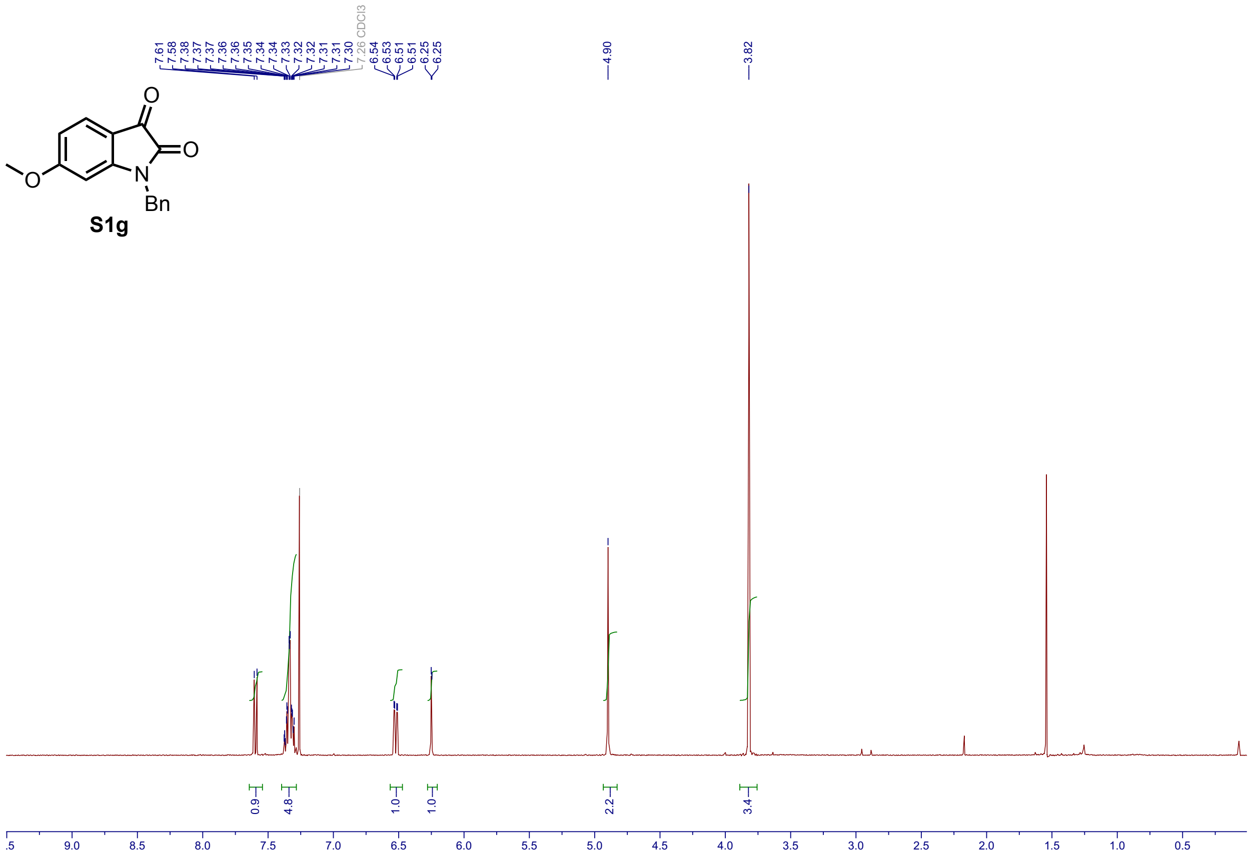

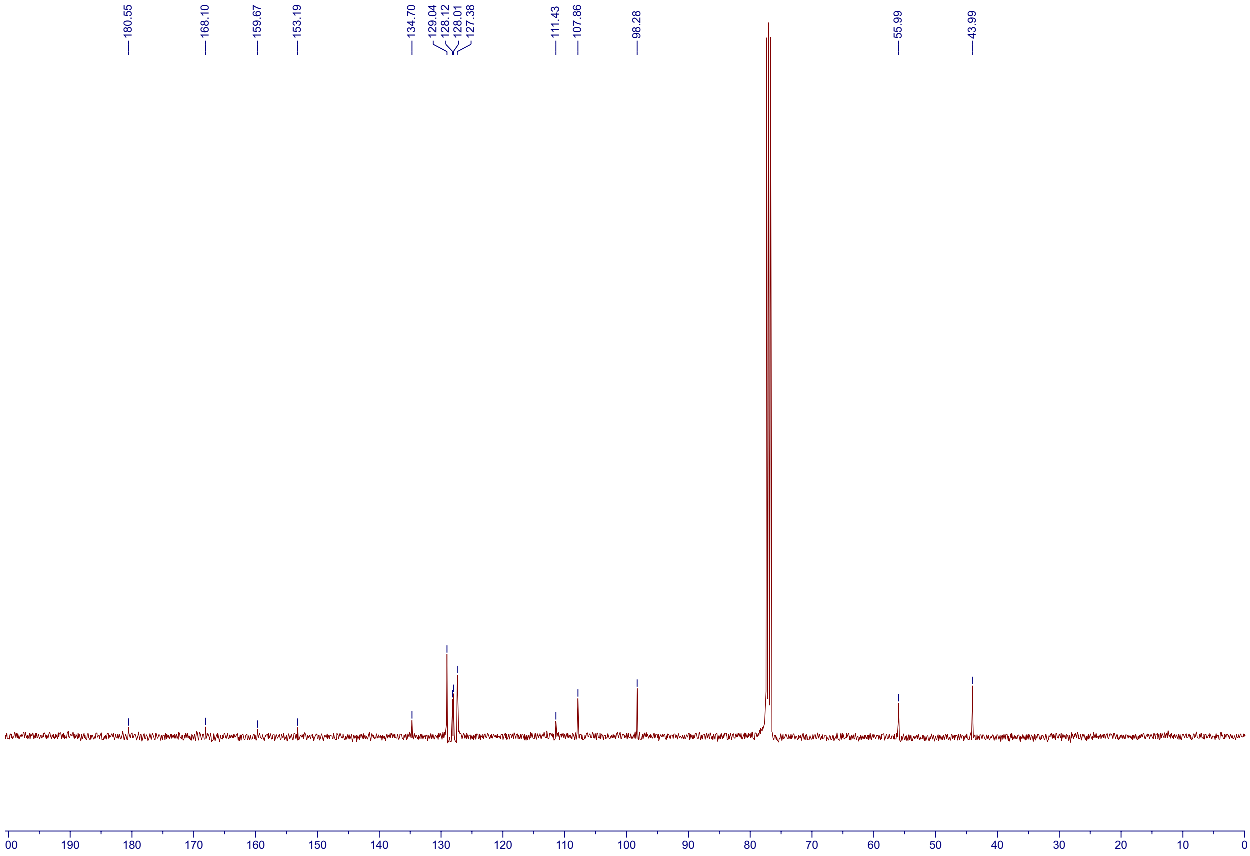

NMRs of *N*-Boc ketimines 1a-g

^{1}H-NMR (400 MHz), ^{13}C{^{1}H}-NMR (101 MHz) of *tert*-butyl (*E*)-(1-benzyl-2-oxoindolin-3-ylidene)carbamate **1a** – $CDCl_3$

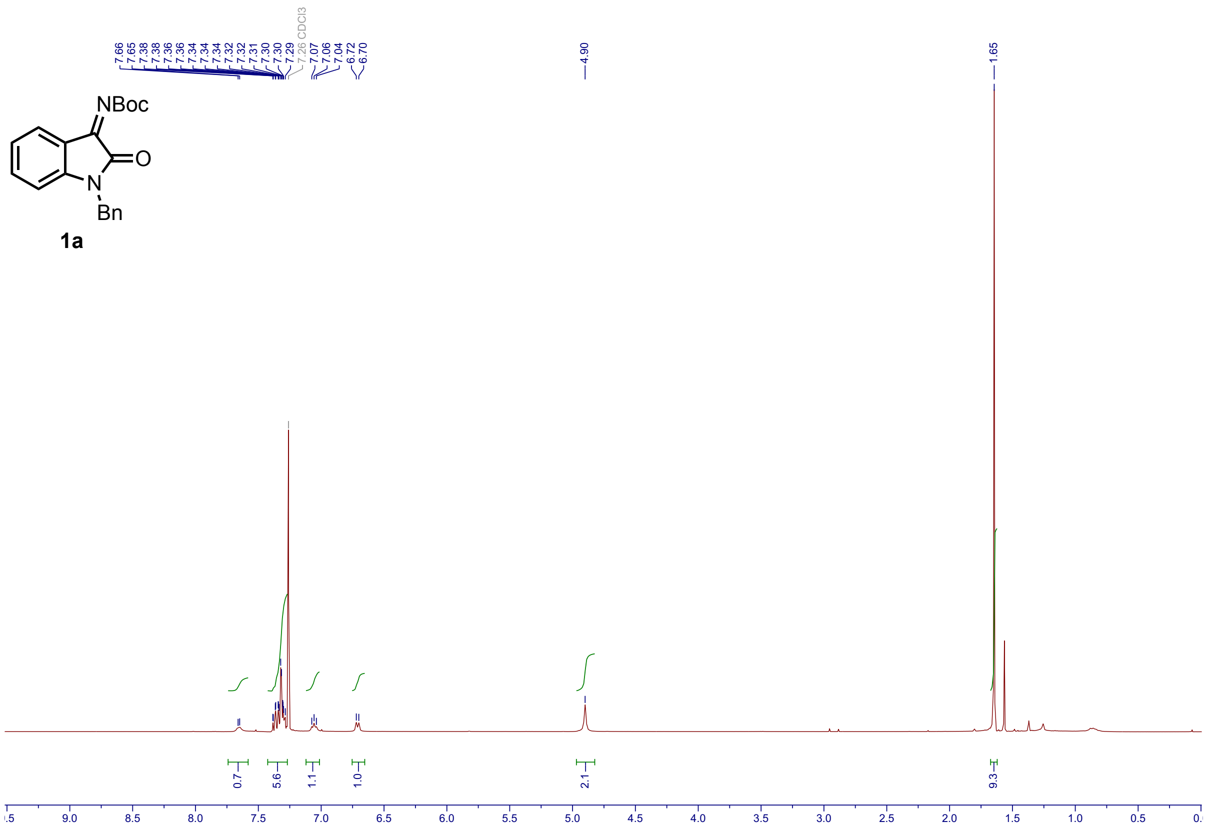

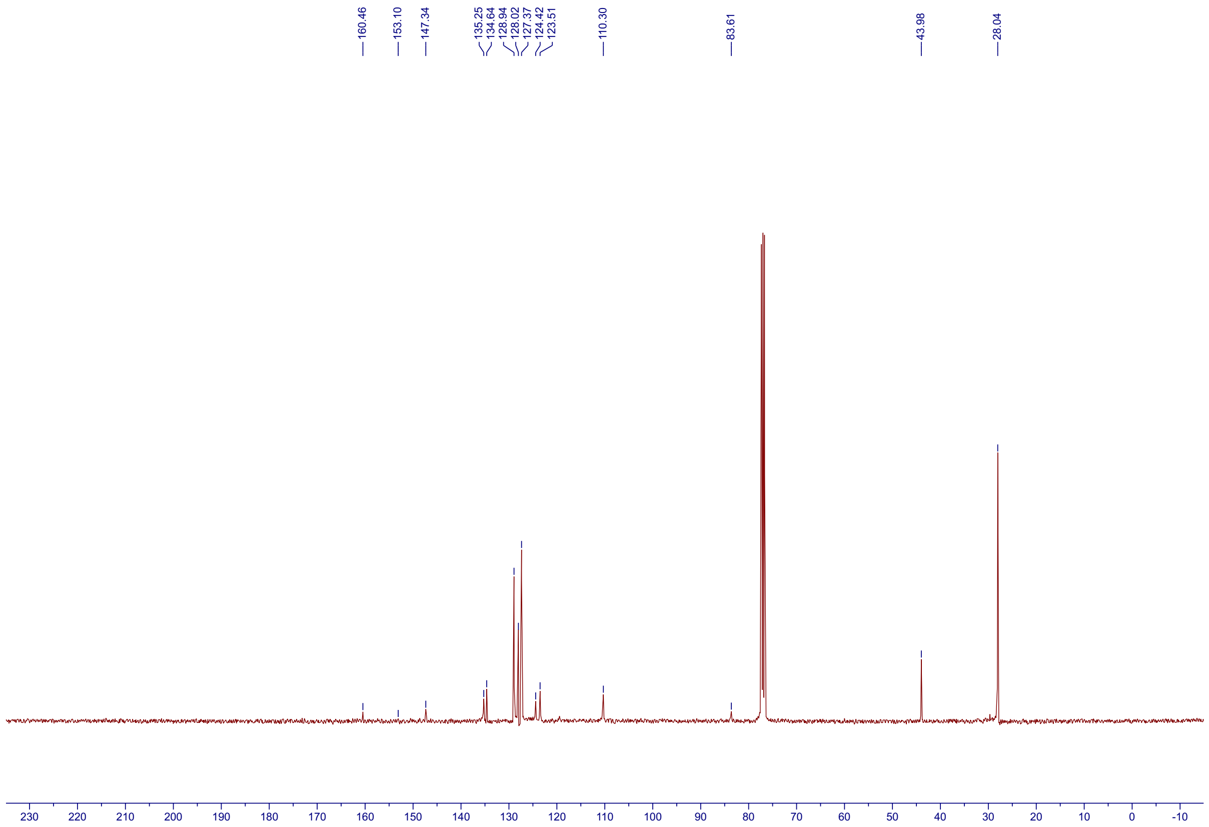

^{1}H-NMR (400 MHz), ^{13}C{^{1}H}-NMR (101 MHz) of *tert*-butyl (*E*)-(2-oxoindolin-3-ylidene)carbamate **1b** – $CDCl_3$

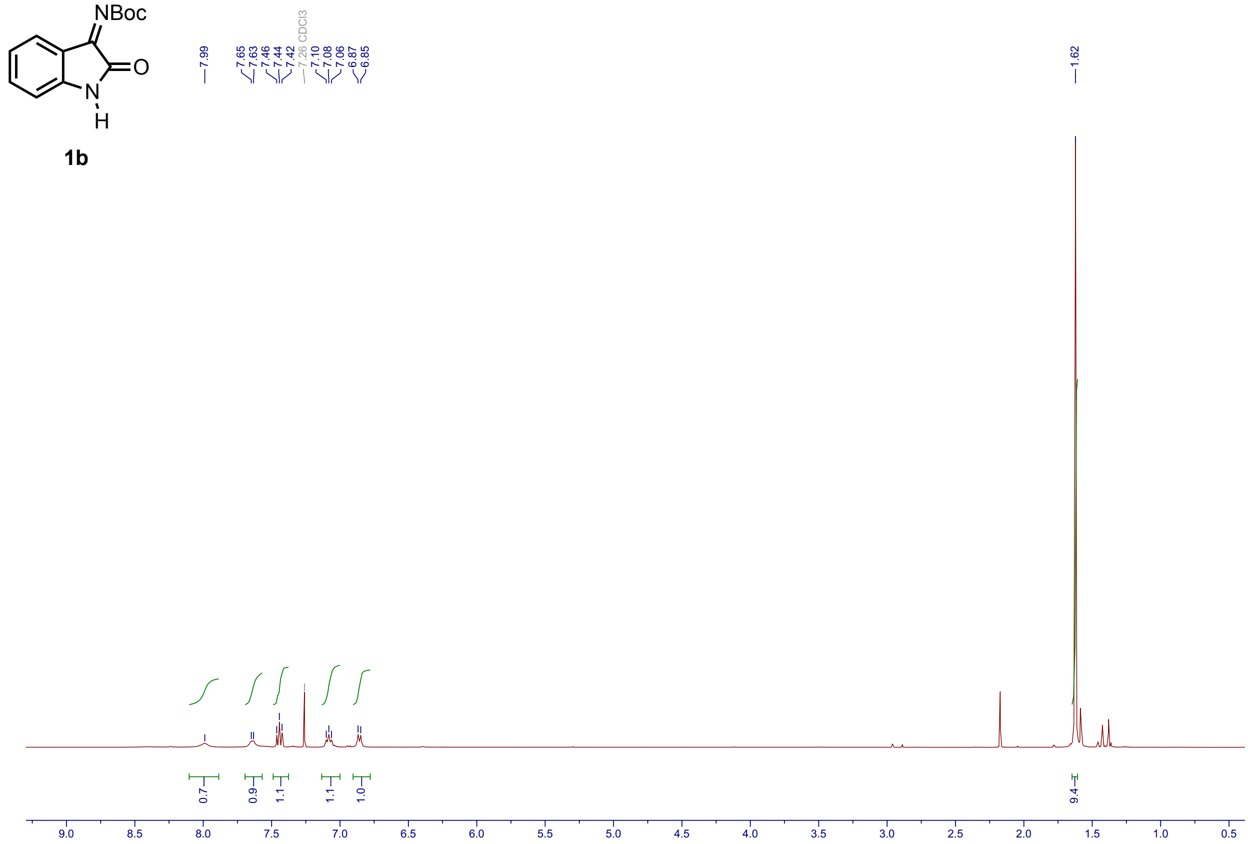

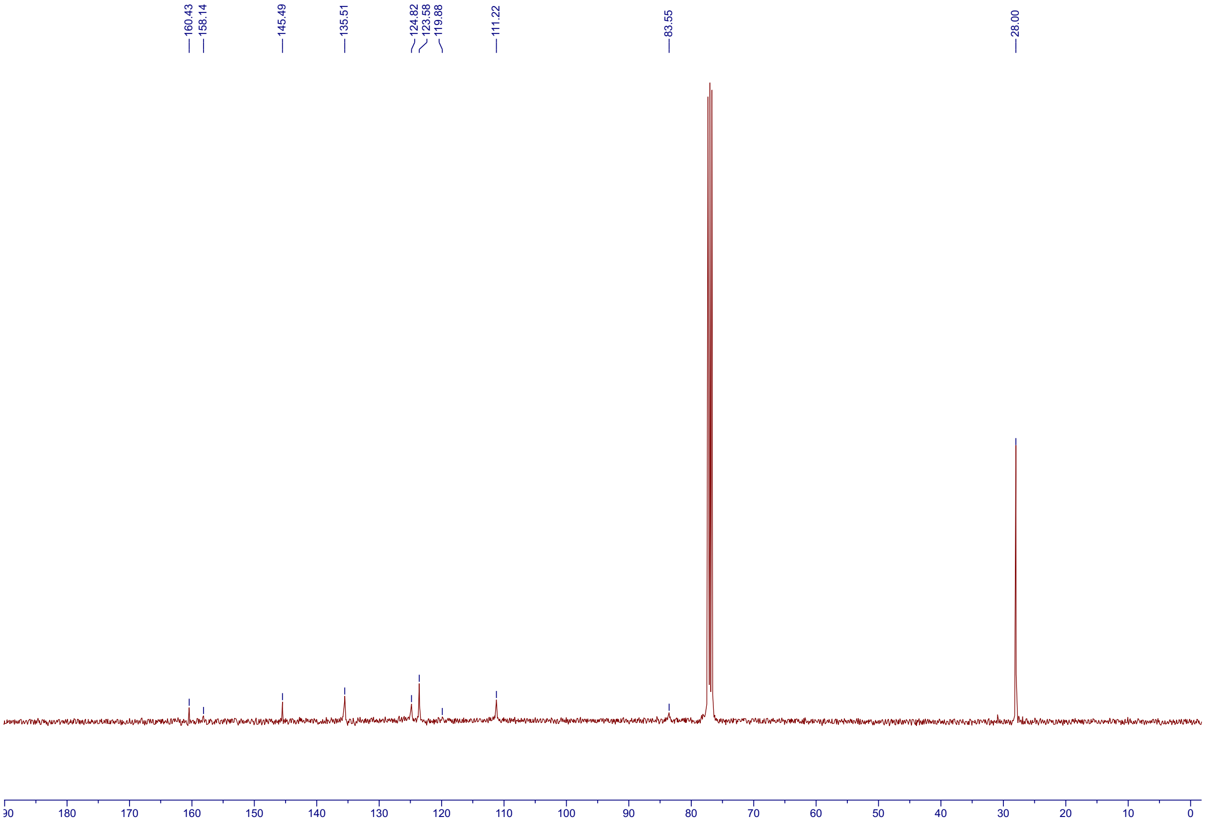

^{1}H-NMR (400 MHz), ^{13}C{^{1}H}-NMR (101 MHz) of *tert*-butyl (*E*)-(1-methyl-2-oxoindolin-3-ylidene)carbamate **1c** – $CDCl_3$

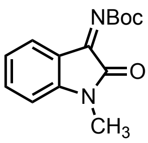

1c

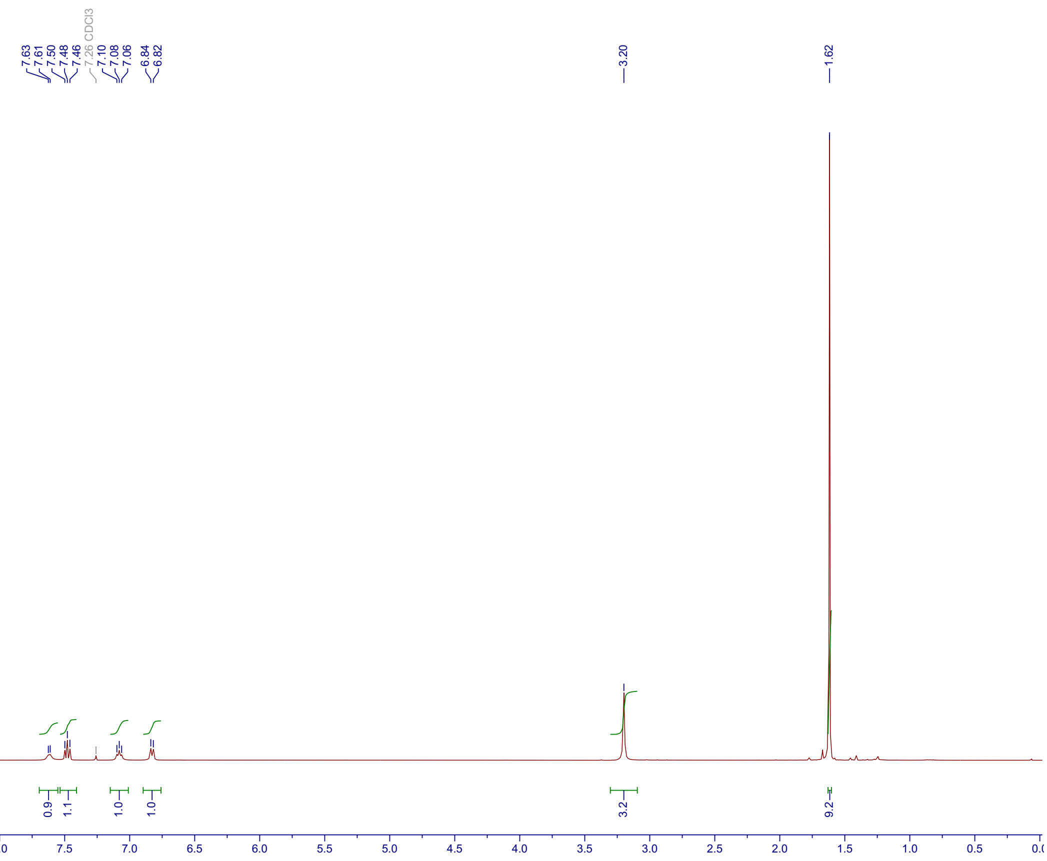

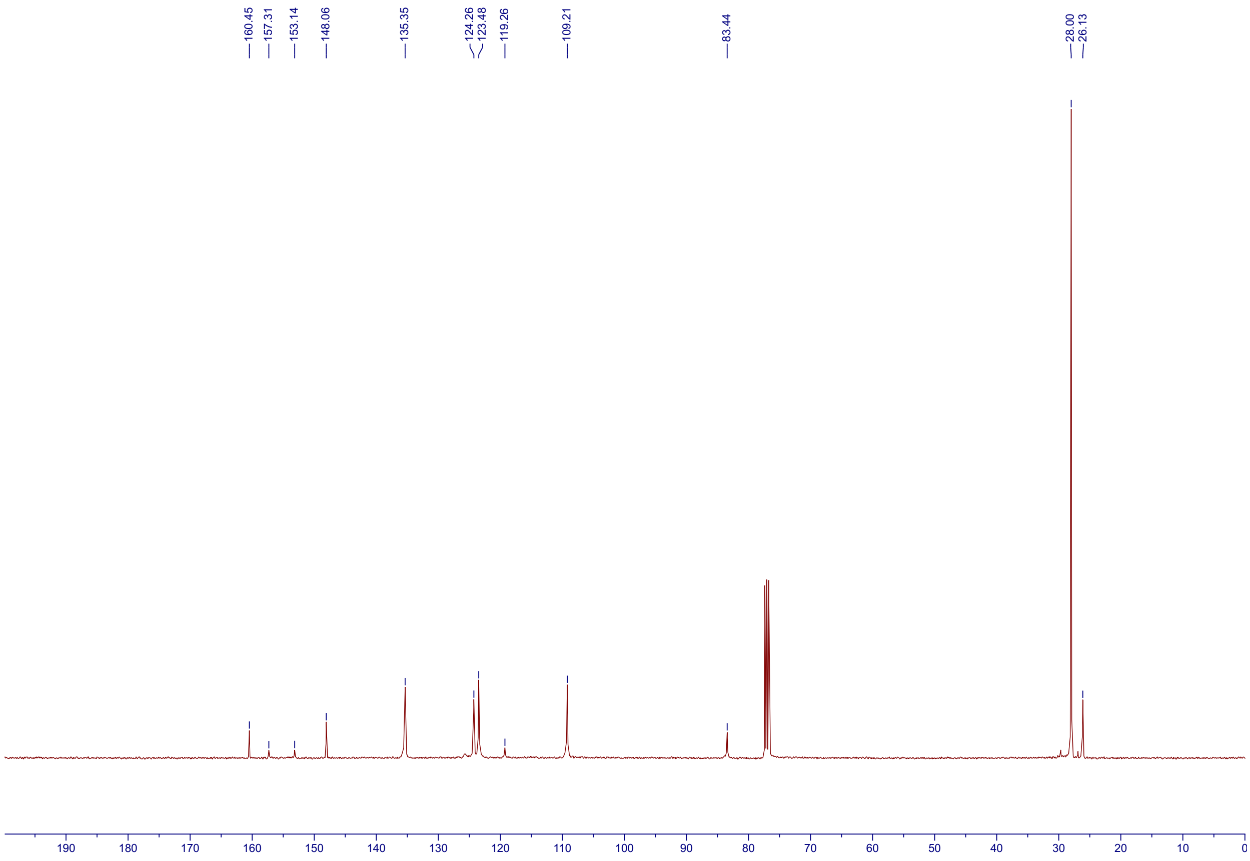

^{1}H-NMR (400 MHz), $^{13}C\{^{1}H\}$-NMR (101 MHz) of *tert*-butyl (*E*)-(5-methoxy-1-methyl-2-oxoindolin-3-ylidene)carbamate **1d**– $CDCl_3$

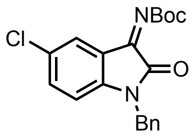

1d

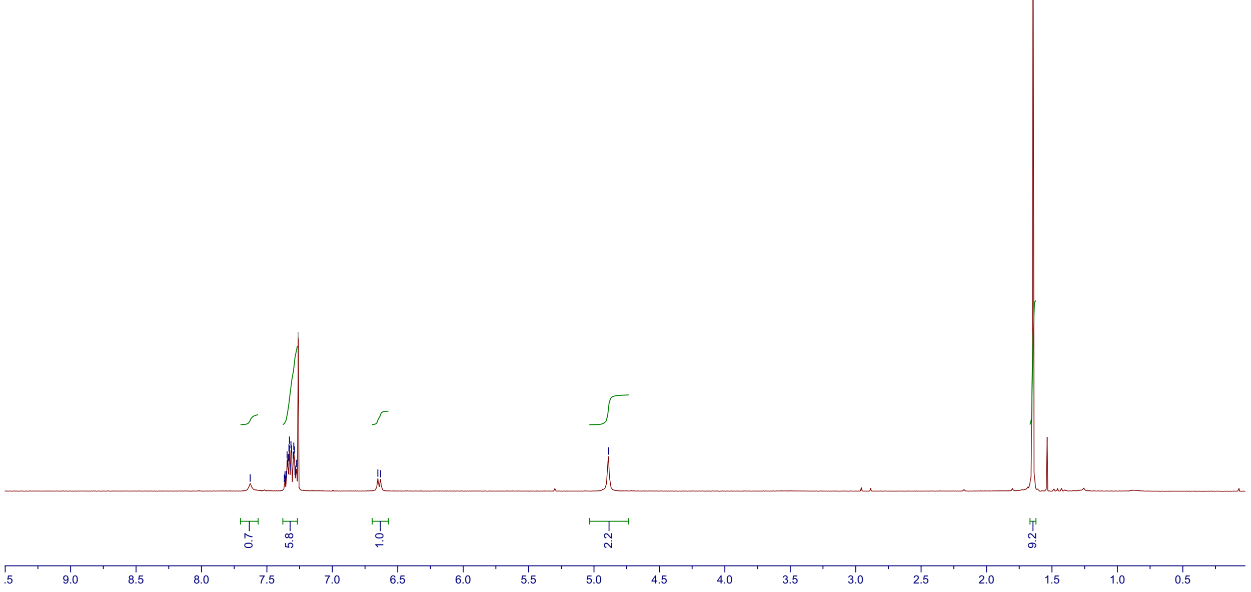

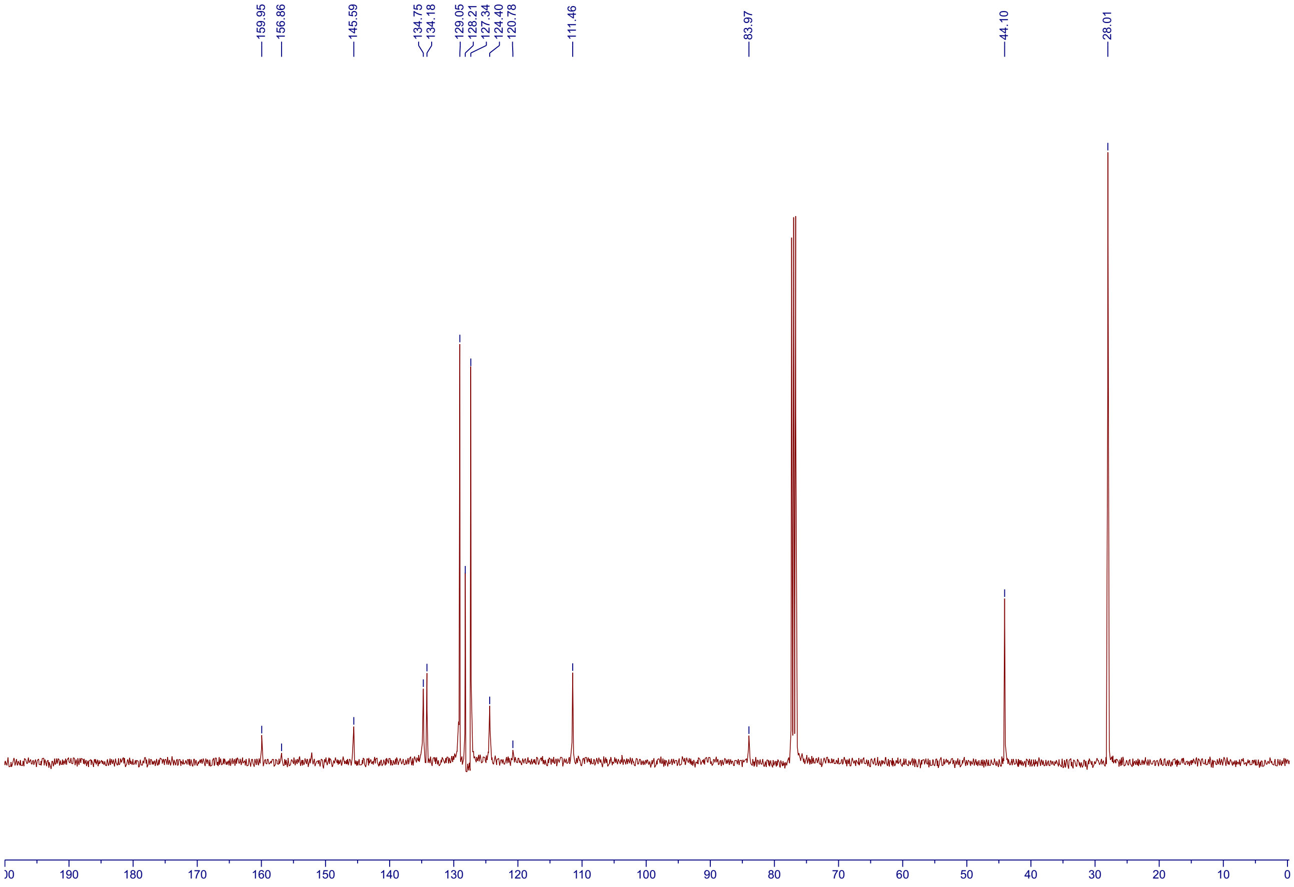

^{1}H-NMR (400 MHz), $^{13}C\{^{1}H\}$-NMR (101 MHz) of *tert*-butyl (*E*)-(1-benzyl-5-methoxy-2-oxoindolin-3-ylidene)carbamate **1e** – $CDCl_3$

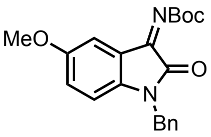

1e

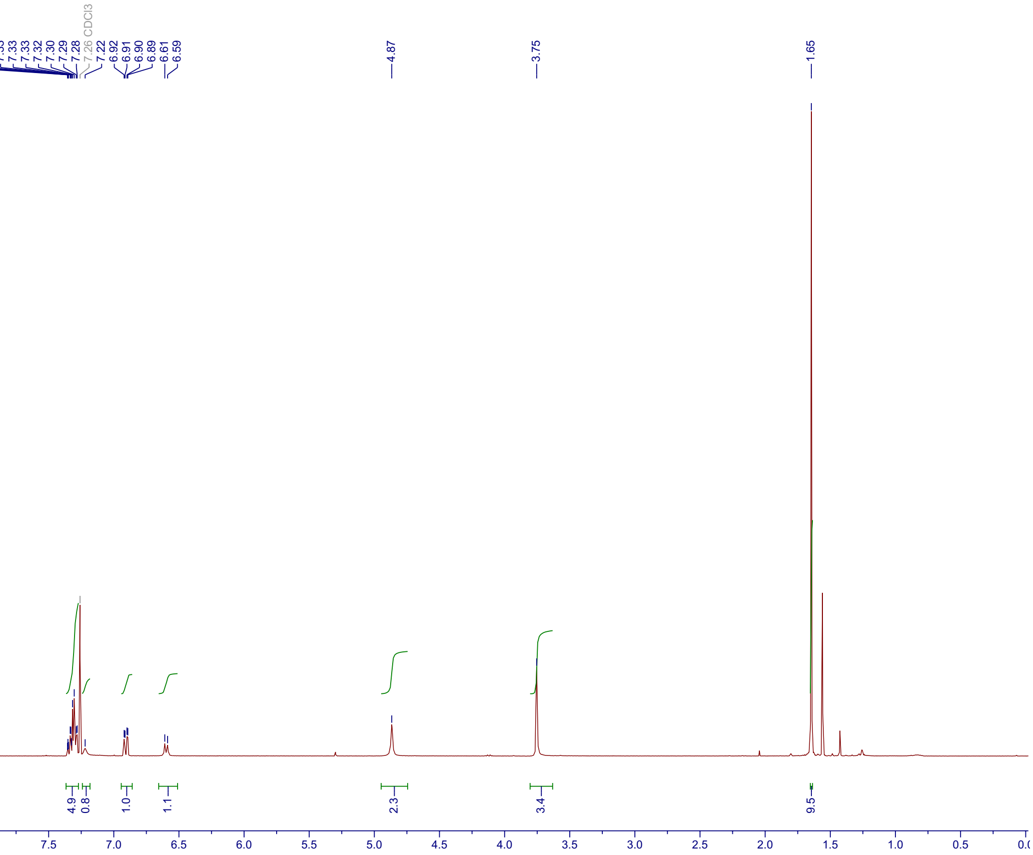

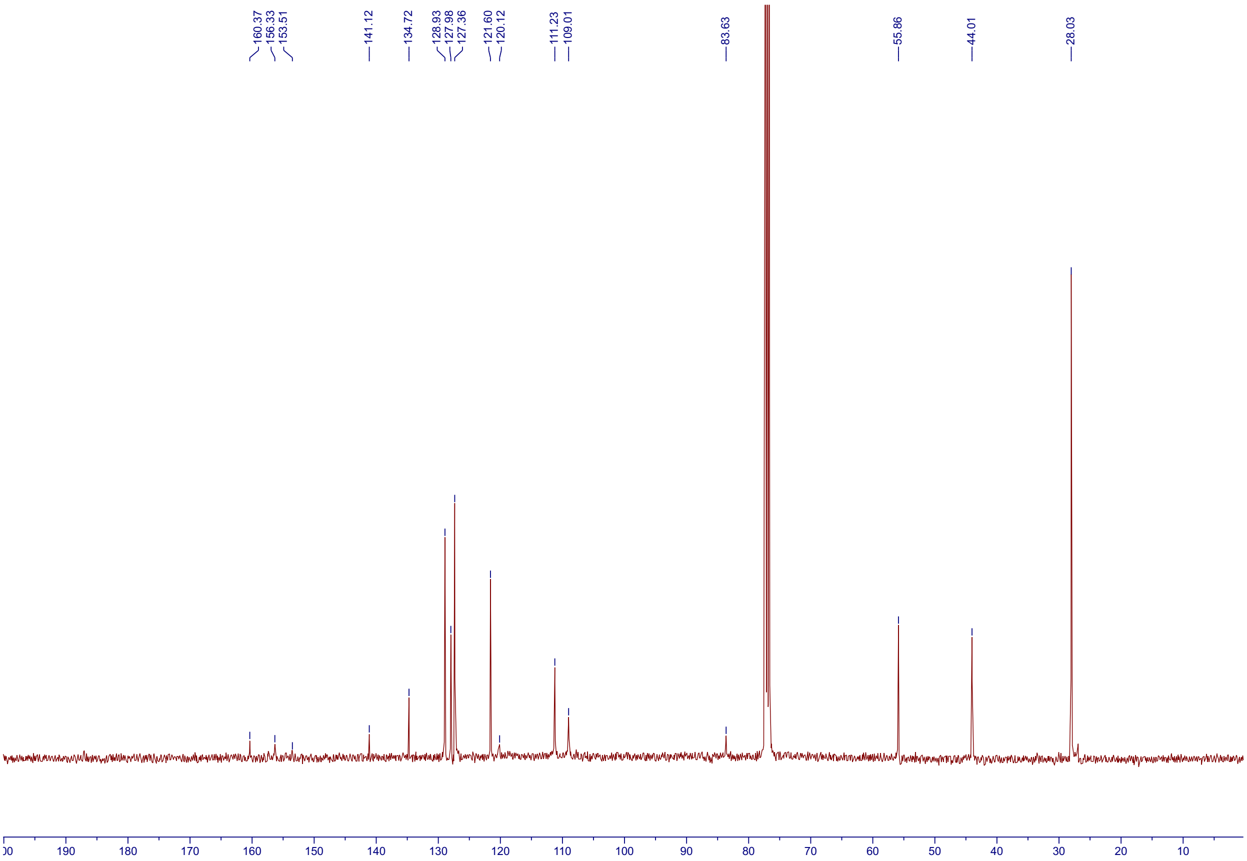

^{1}H-NMR (400 MHz), ^{13}C{^{1}H}-NMR (101 MHz) of *tert*-butyl (*E*)-(1-benzyl-6-chloro-2-oxoindolin-3-ylidene)carbamate **1f** – $CDCl_3$

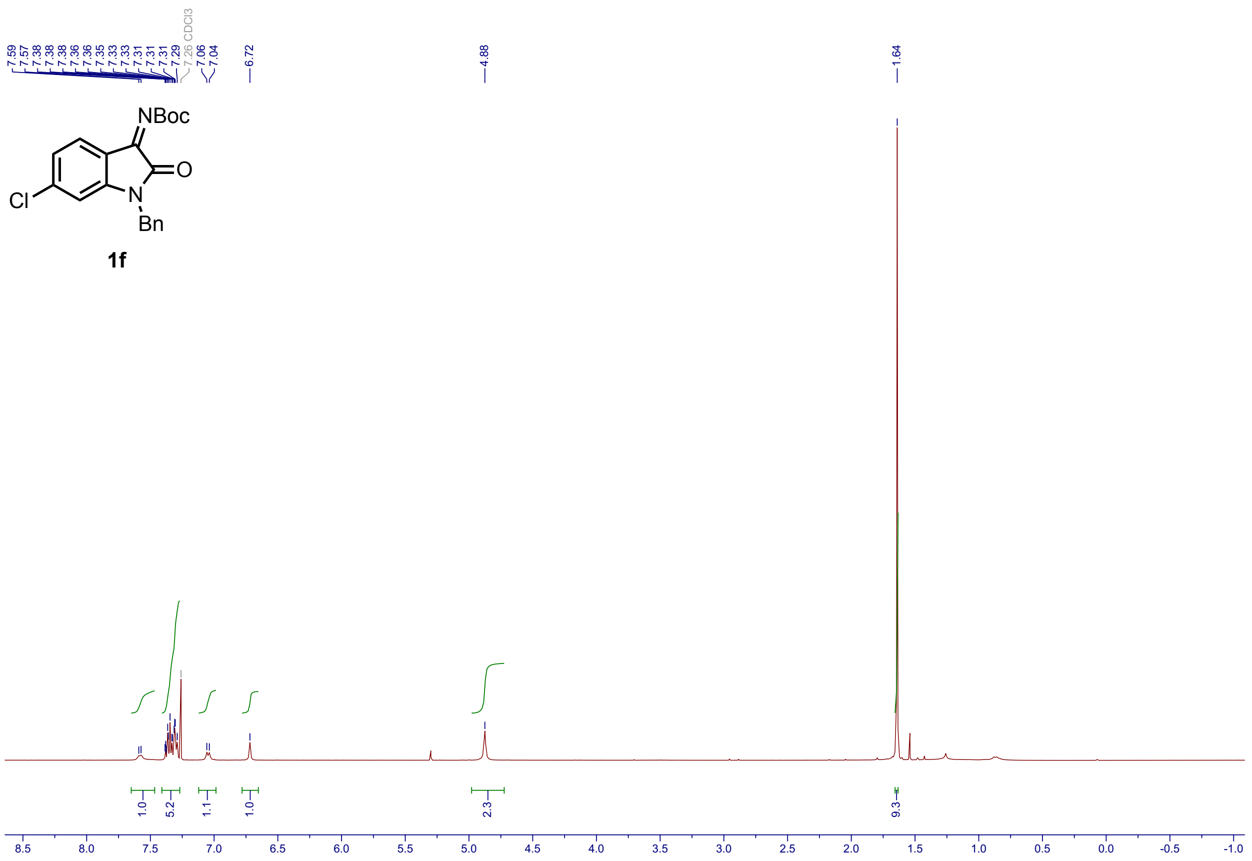

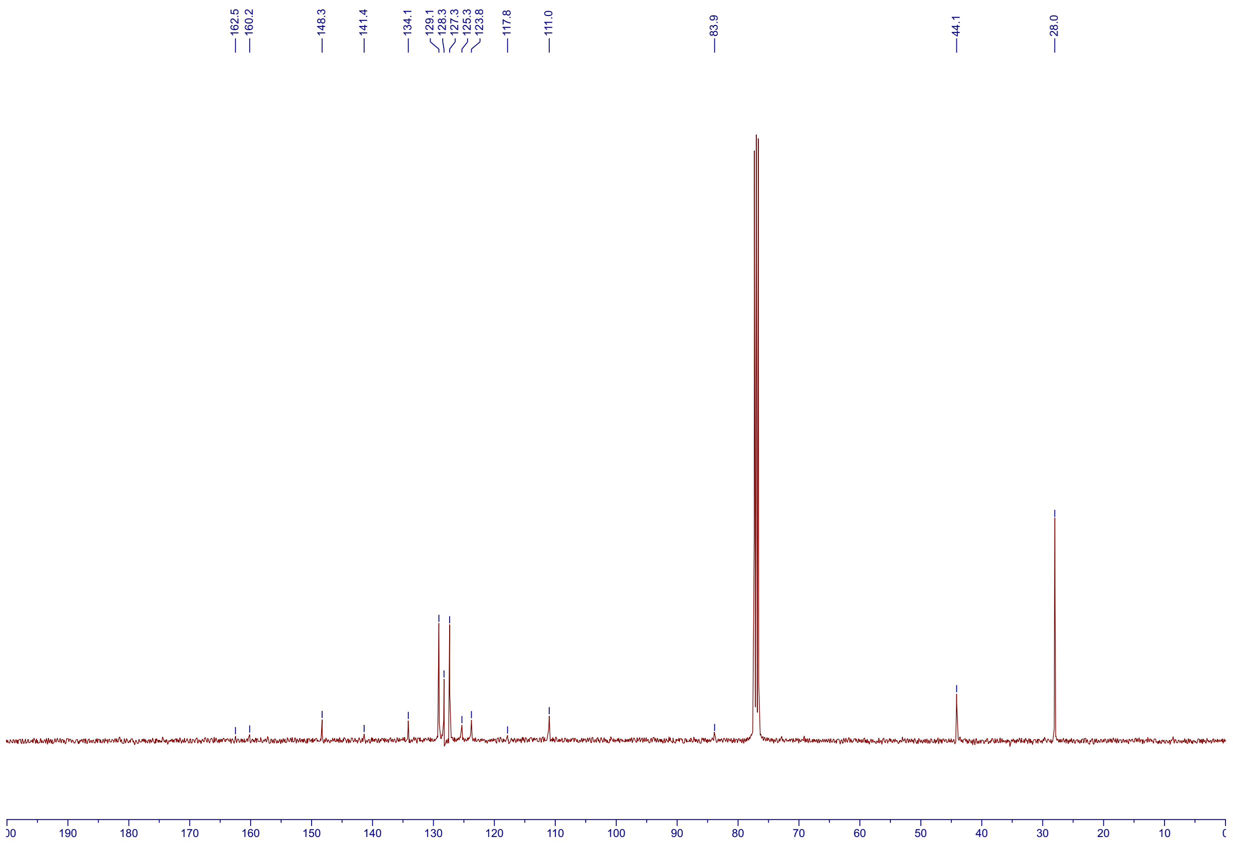

^{1}H-NMR (400 MHz), ^{13}C{^{1}H}-NMR (101 MHz) of *tert*-butyl (*E*)-(1-benzyl-6-methoxy-2-oxoindolin-3-ylidene)carbamate **1g**– $CDCl_3$

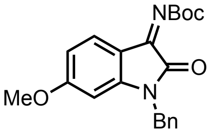

1g

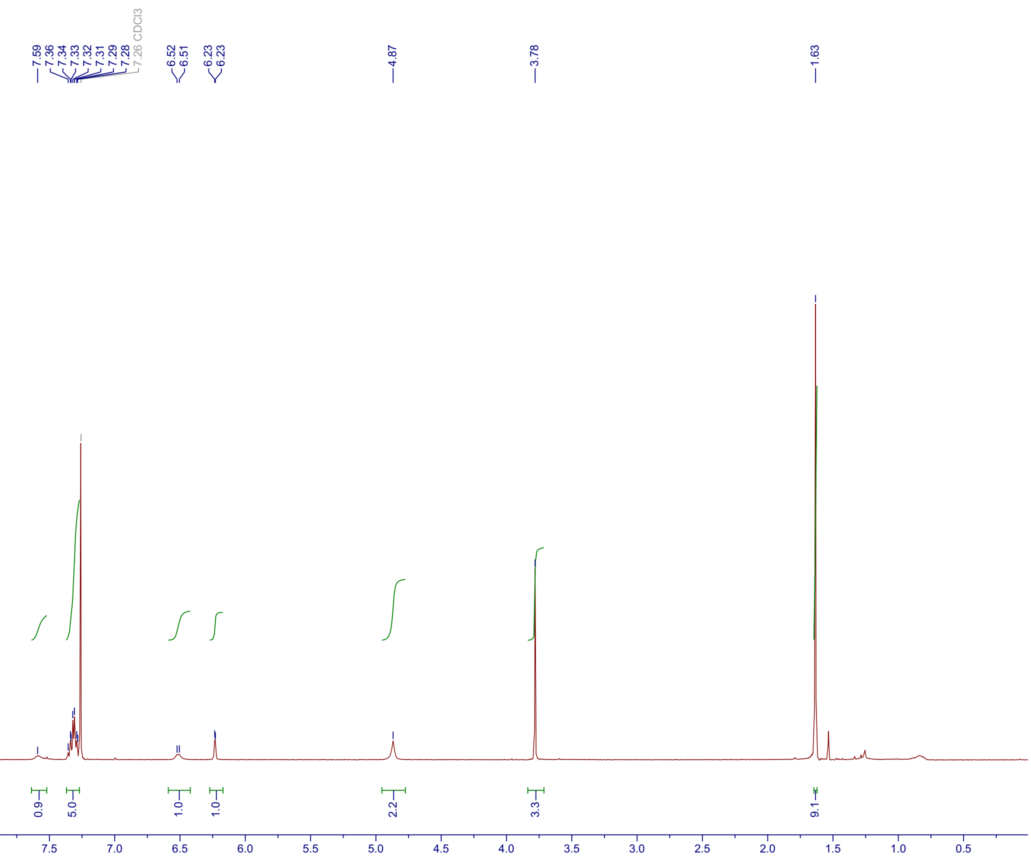

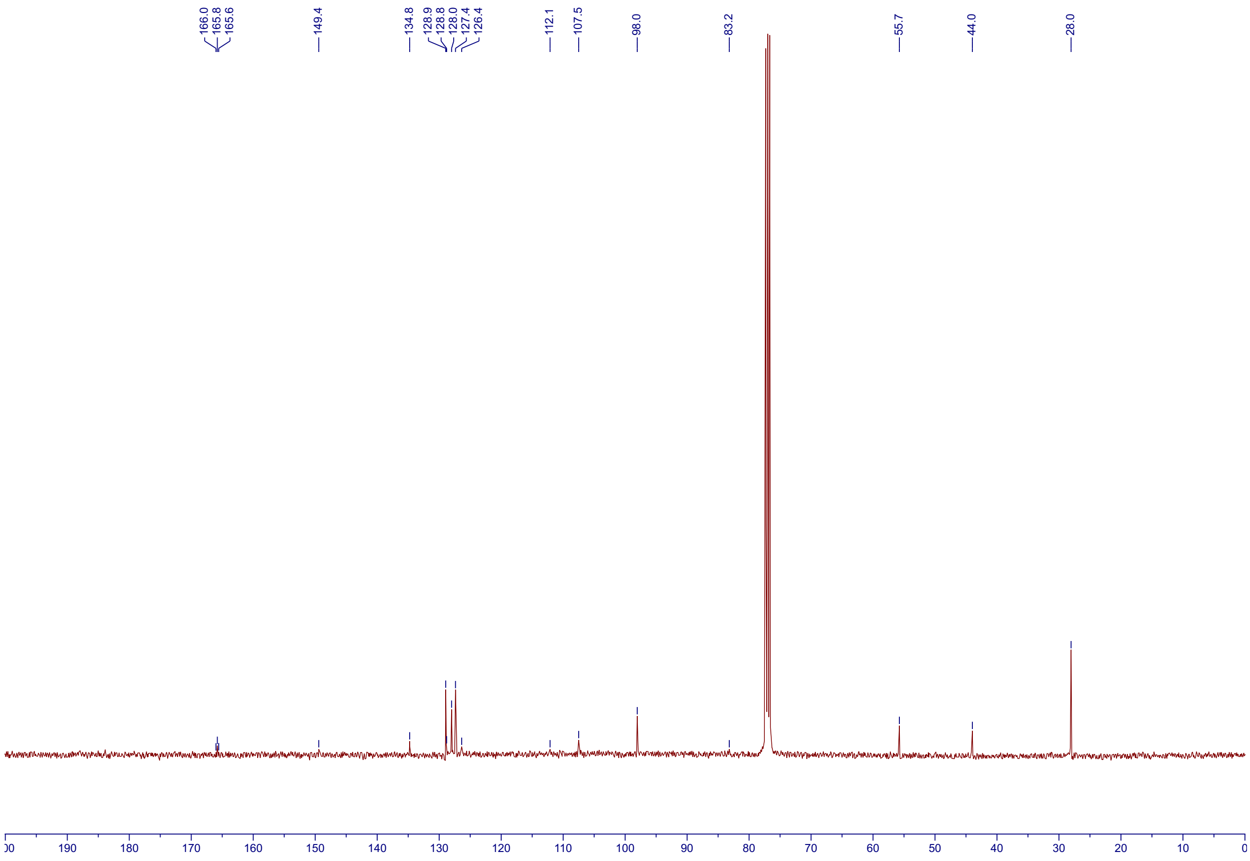

NMRs of Oxindoles 3

^{1}H-NMR (400 MHz), ^{13}C{^{1}H}-NMR (101 MHz) of *tert*-butyl ((*S*)-1-benzyl-3-((*R*)-(4-chlorophenyl)(nitro)methyl)-2-oxoindolin-3-yl)carbamate **3aa** – $CDCl_3$

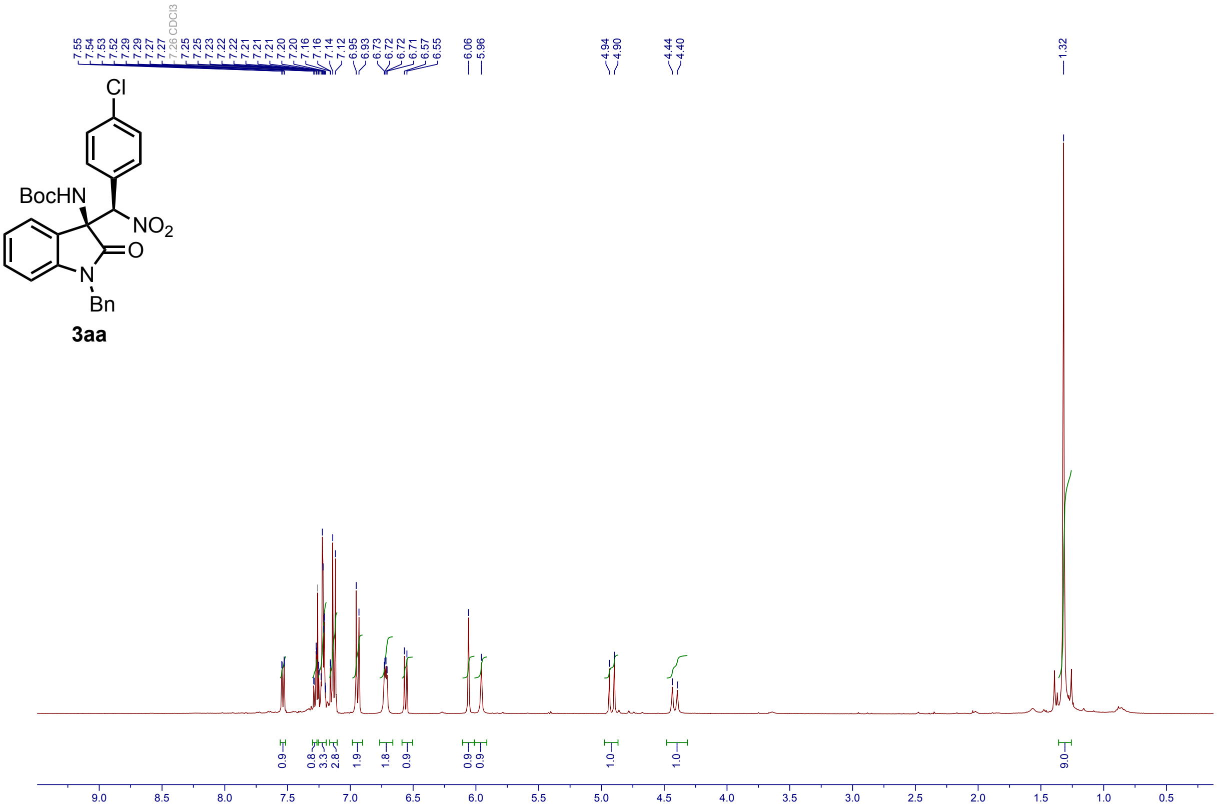

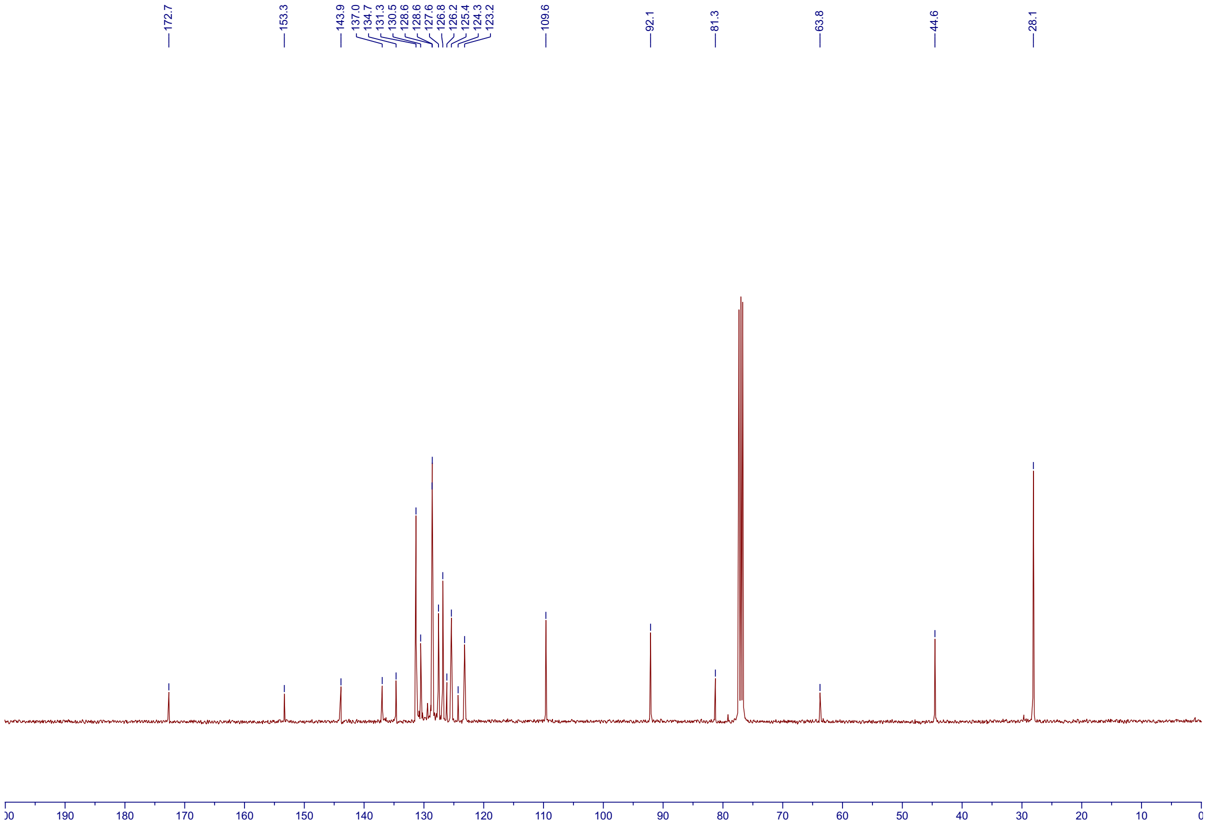

^{1}H-NMR (400 MHz), ^{13}C{^{1}H}-NMR (101 MHz) of *tert*-butyl ((*S*)-1-benzyl-3-((*R*)-(4-bromophenyl)(nitro)methyl)-2-oxoindolin-3-yl)carbamate **3ab** – $CDCl_3$

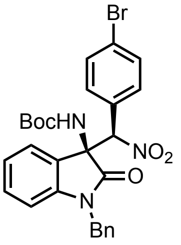

3ab

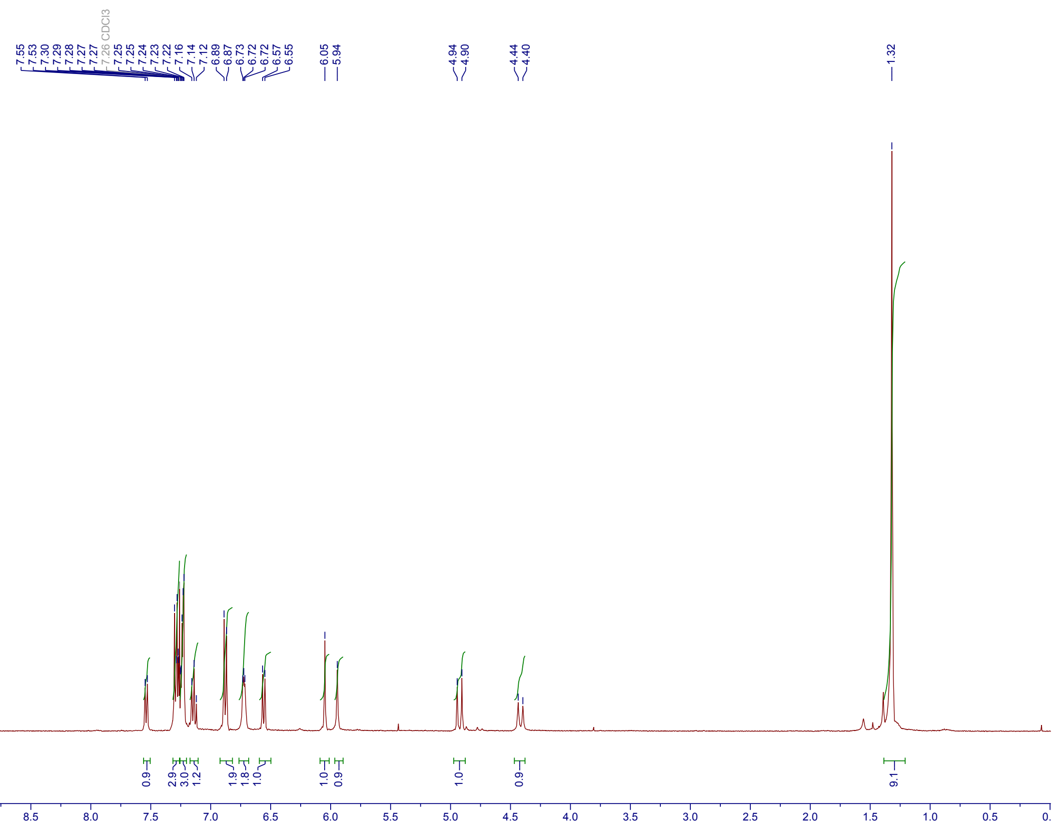

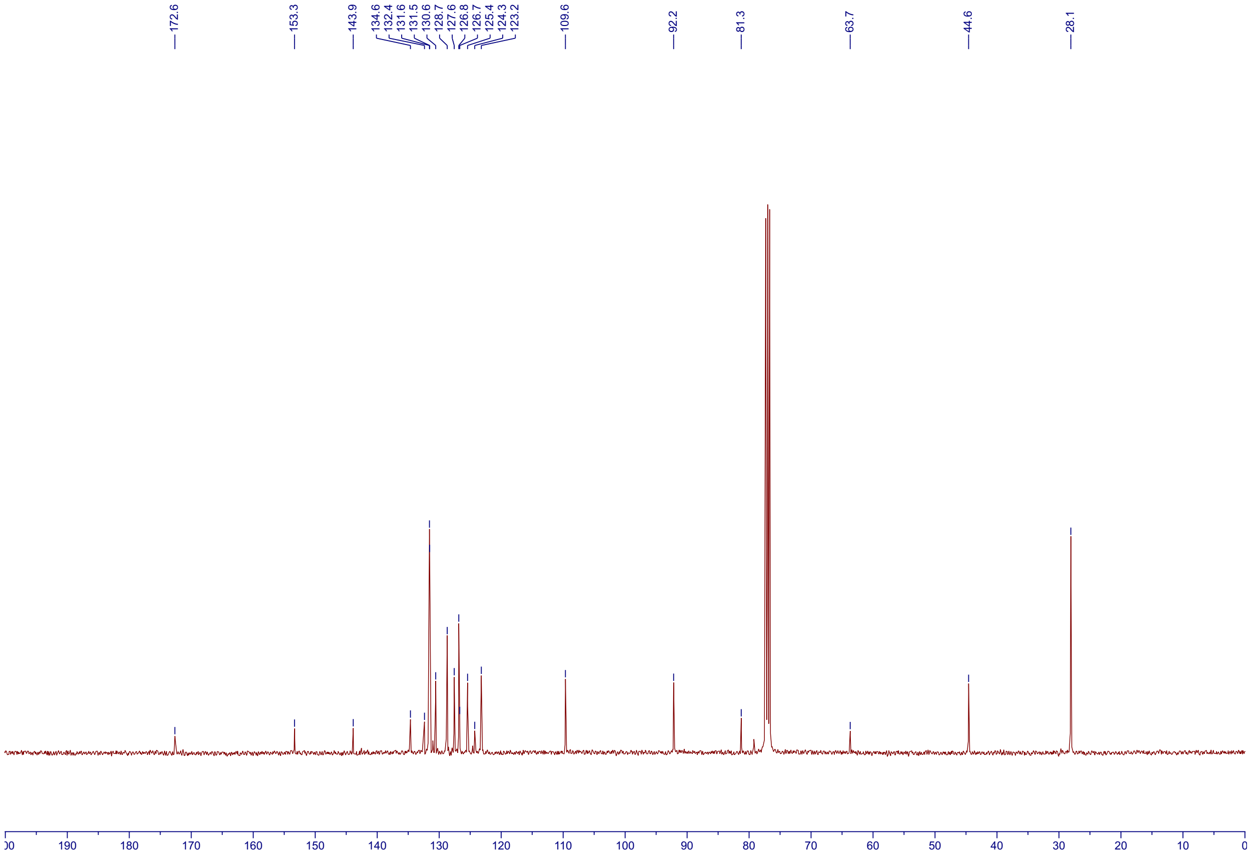

^{1}H-NMR (400 MHz), ^{13}C{^{1}H}-NMR (101 MHz) of *tert*-butyl ((*S*)-1-benzyl-3-((*R*)-nitro(*p*-tolyl)methyl)-2-oxoindolin-3-yl)carbamate **3ac** – $CDCl_3$

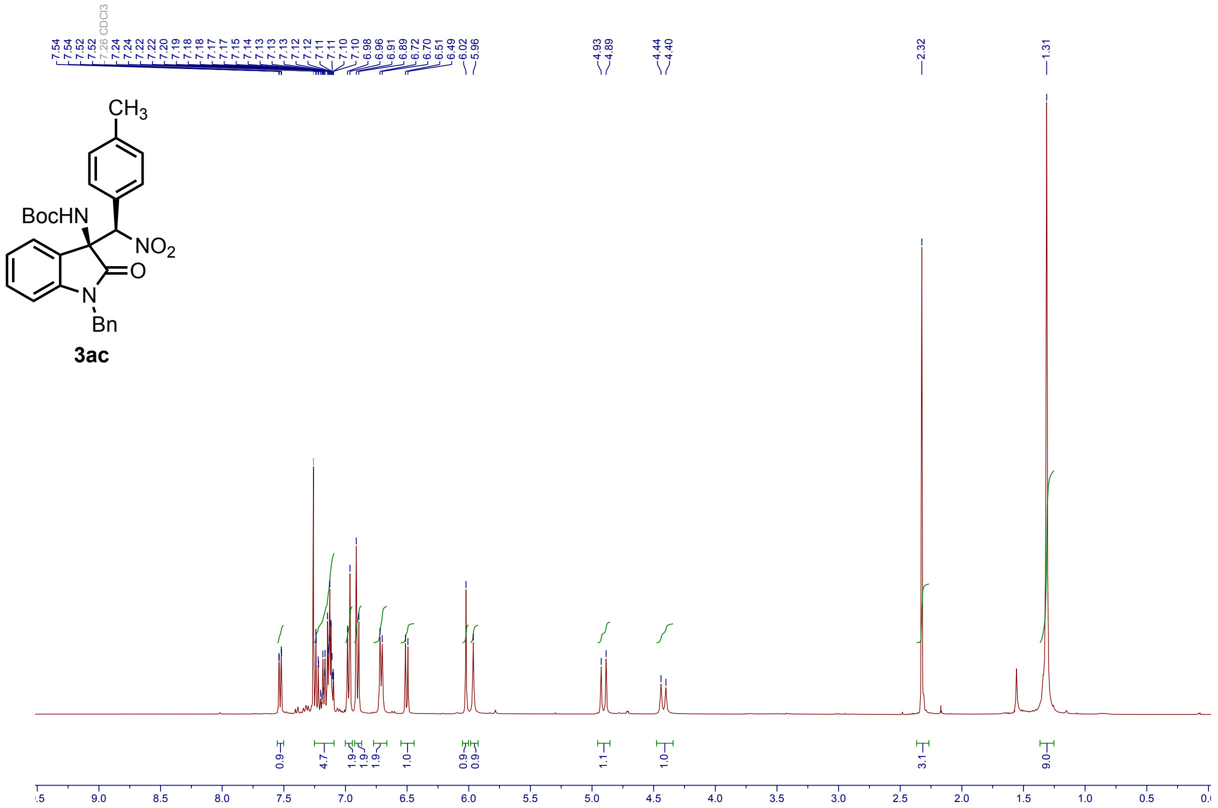

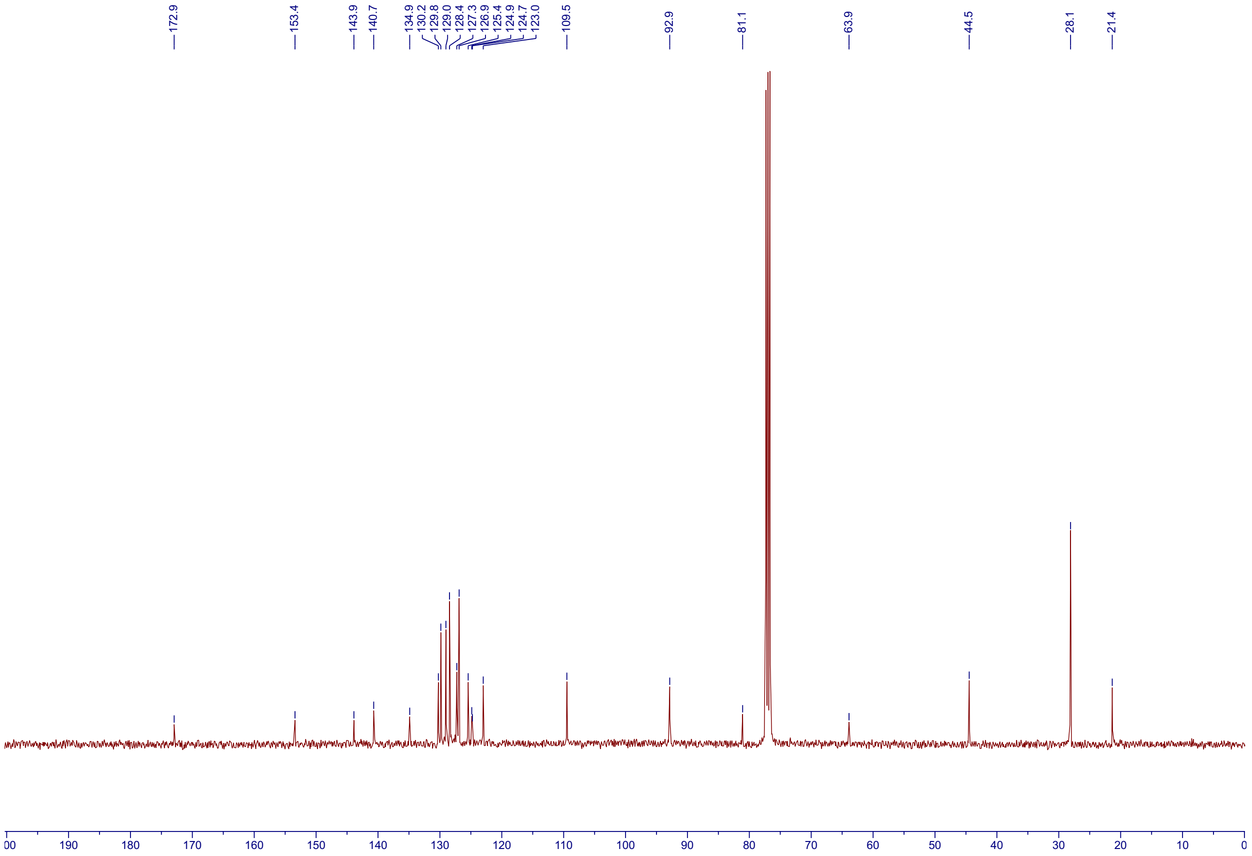

^{1}H-NMR (400 MHz), ^{13}C{^{1}H}-NMR (101 MHz) of *tert*-butyl ((*S*)-1-benzyl-3-((*R*)-(3-chlorophenyl)(nitro)methyl)-2-oxoindolin-3-yl)carbamate **3ad**

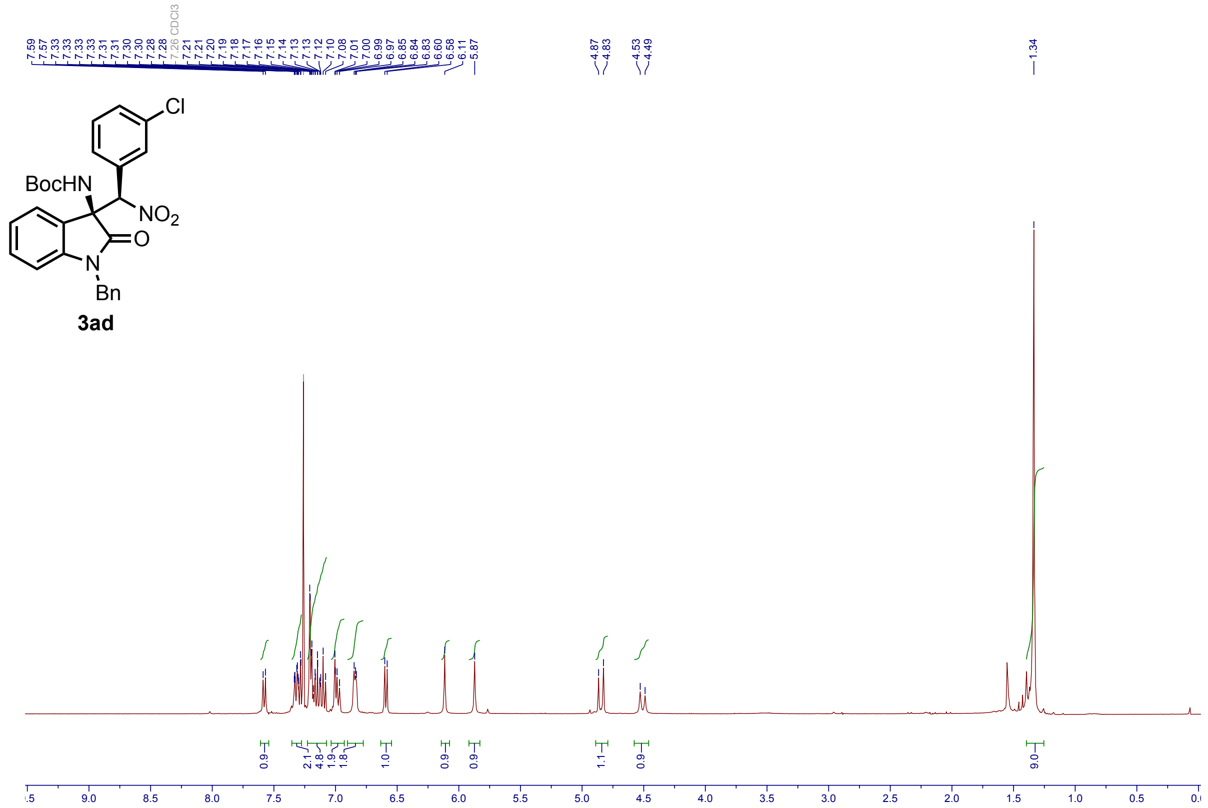

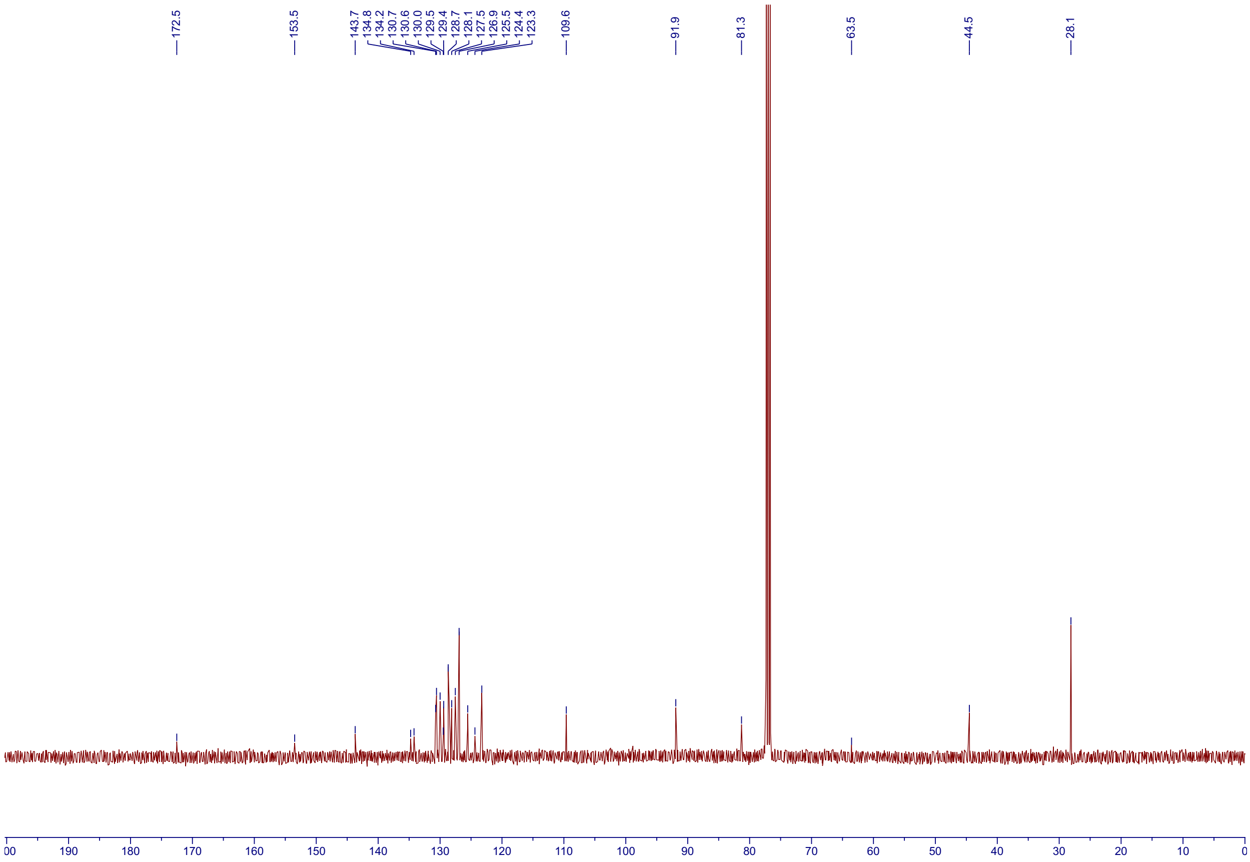

^{1}H-NMR (400 MHz), ^{13}C{^{1}H}-NMR (101 MHz) of *tert*-butyl ((*S*)-1-benzyl-3-((*R*)-(3-bromophenyl)(nitro)methyl)-2-oxoindolin-3-yl)carbamate **3ae**

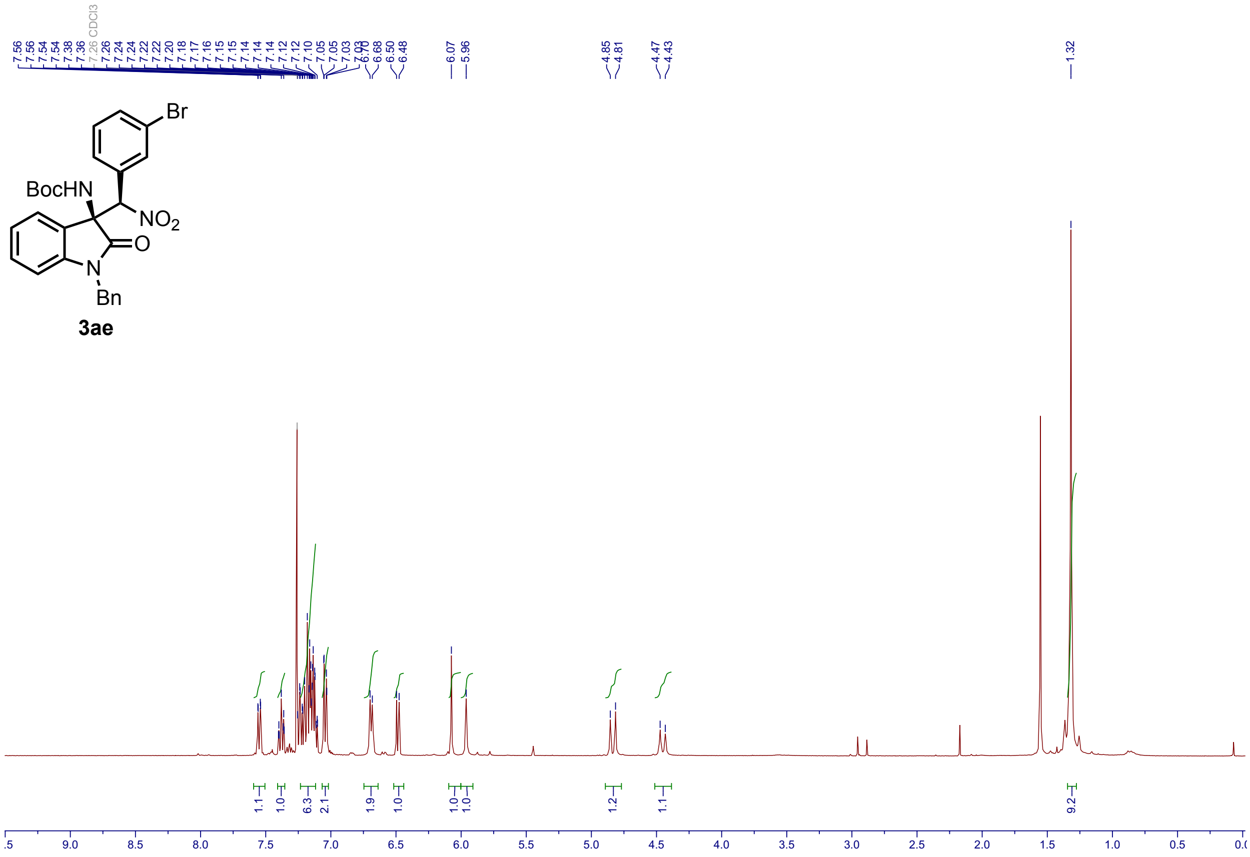

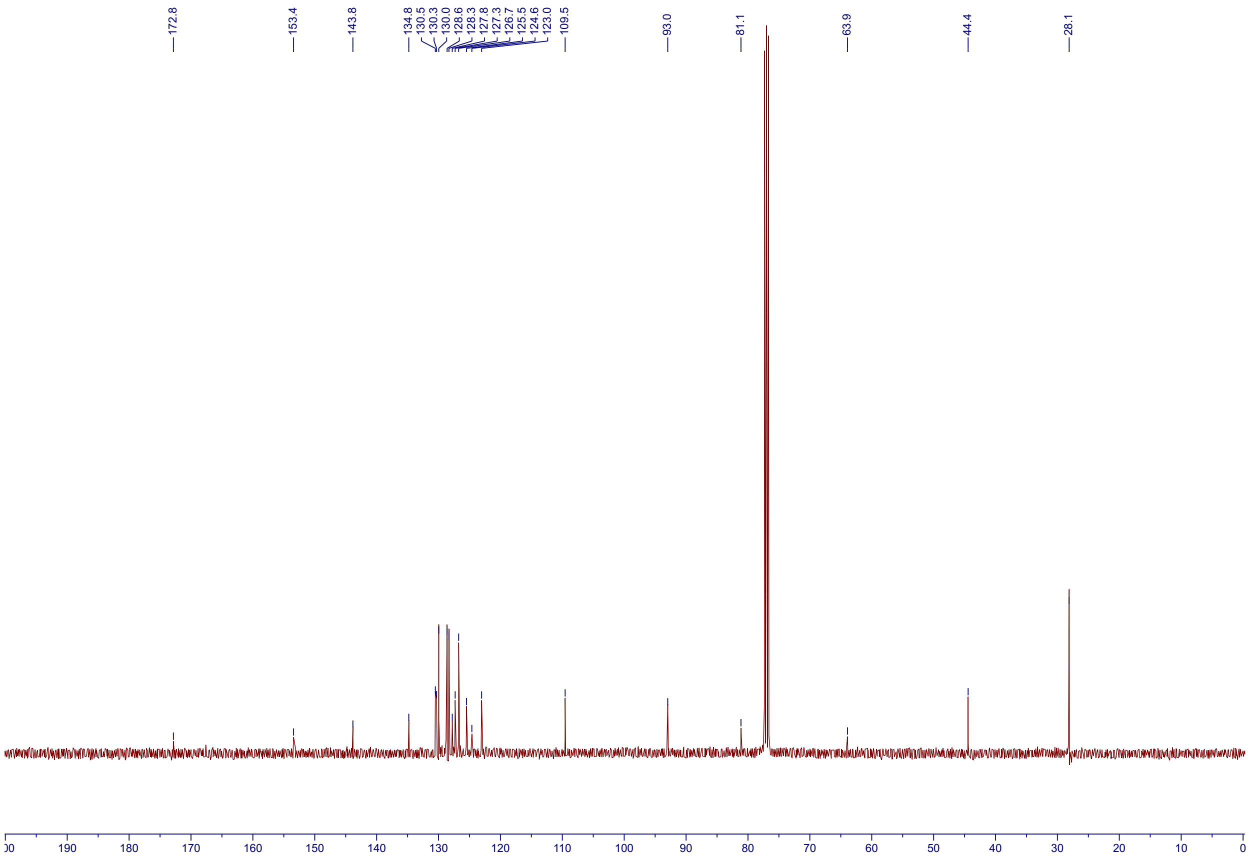

^{1}H-NMR (400 MHz), ^{13}C{^{1}H}-NMR (101 MHz) of *tert*-butyl ((*S*)-1-benzyl-3-((*R*)-nitro(*m*-tolyl)methyl)-2-oxoindolin-3-yl)carbamate **3af**

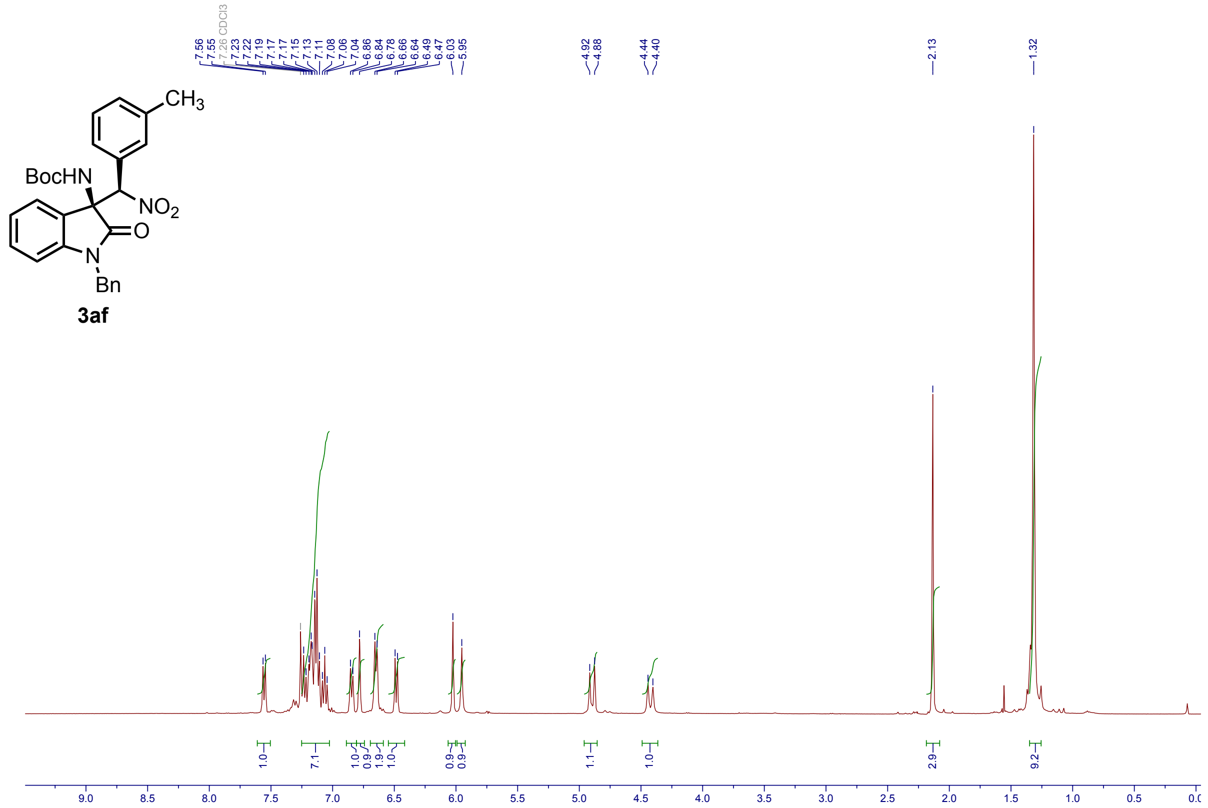

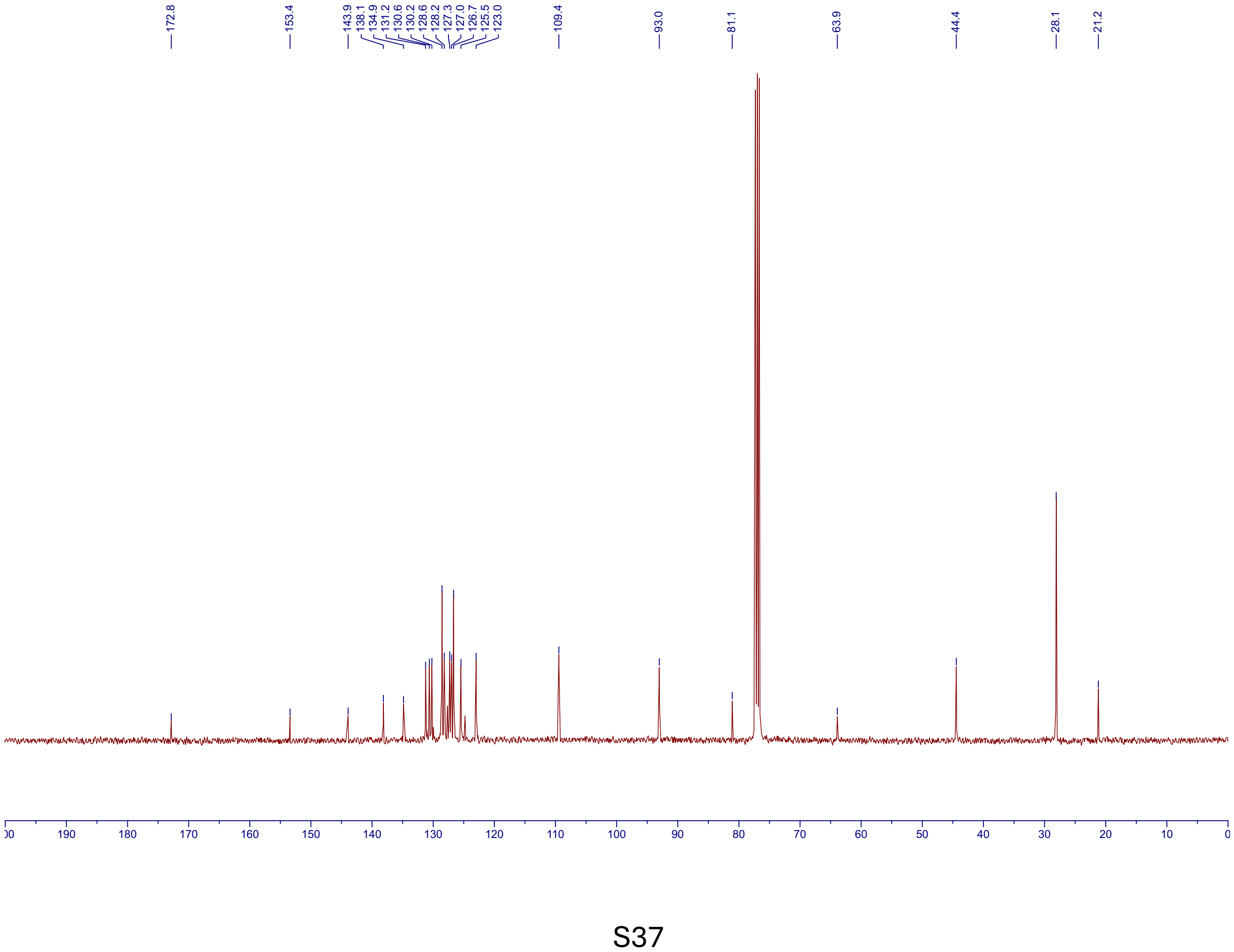

^{1}H-NMR (400 MHz), ^{13}C{^{1}H}-NMR (101 MHz) of *tert*-butyl ((*S*)-1-benzyl-3-((*R*)-nitro(phenyl)methyl)-2-oxoindolin-3-yl)carbamate **3ag**

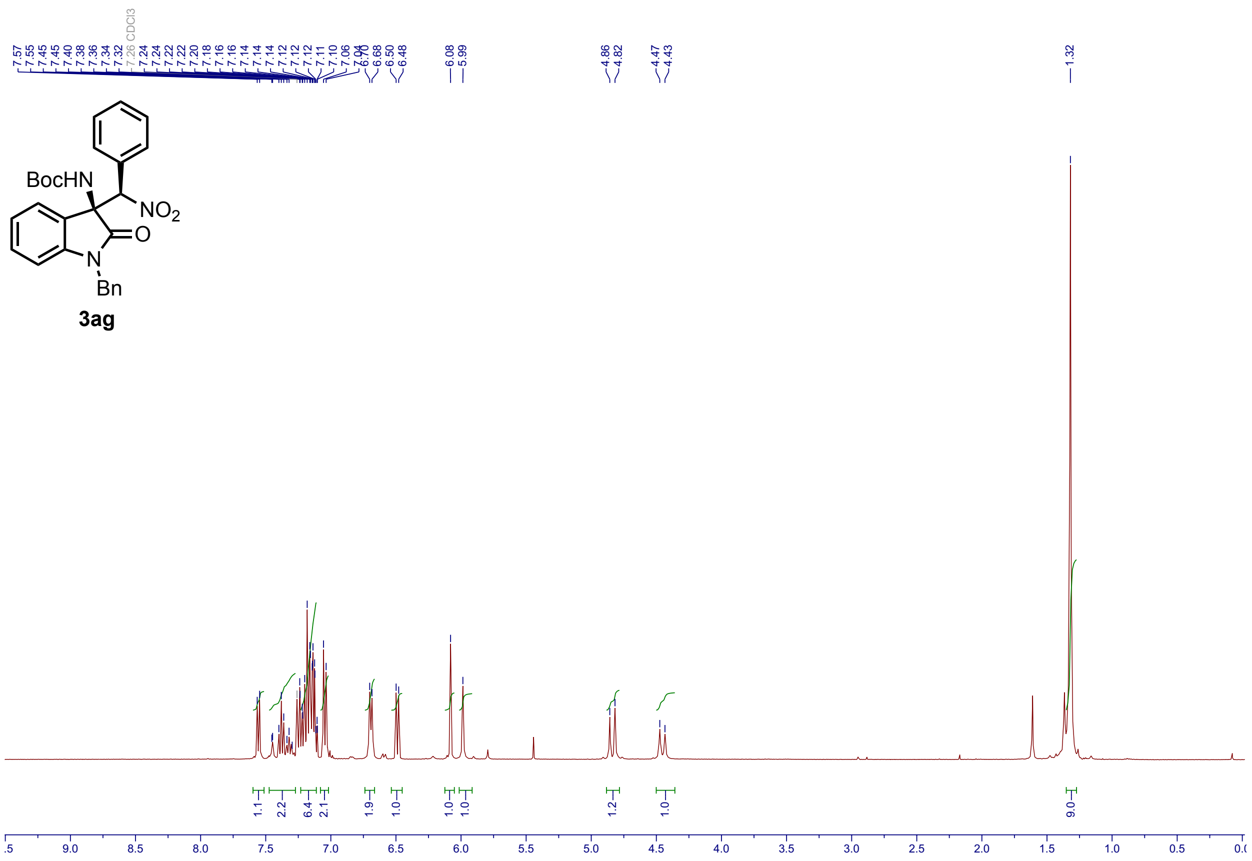

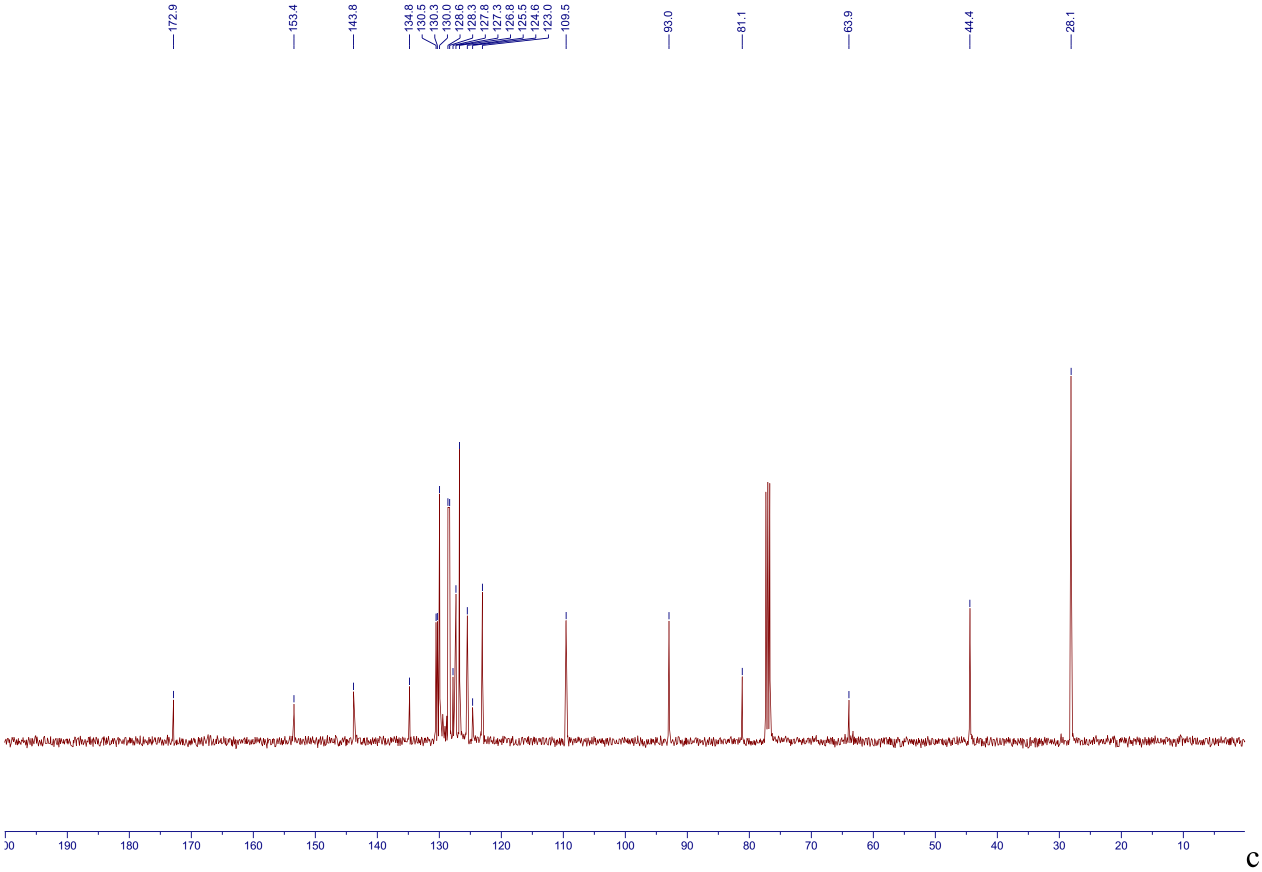

c

^{1}H-NMR (400 MHz), ^{13}C{^{1}H}-NMR (101 MHz) of *tert*-butyl ((*S*)-3-((*R*)-(4-chlorophenyl)(nitro)methyl)-2-oxoindolin-3-yl)carbamate **3ba**

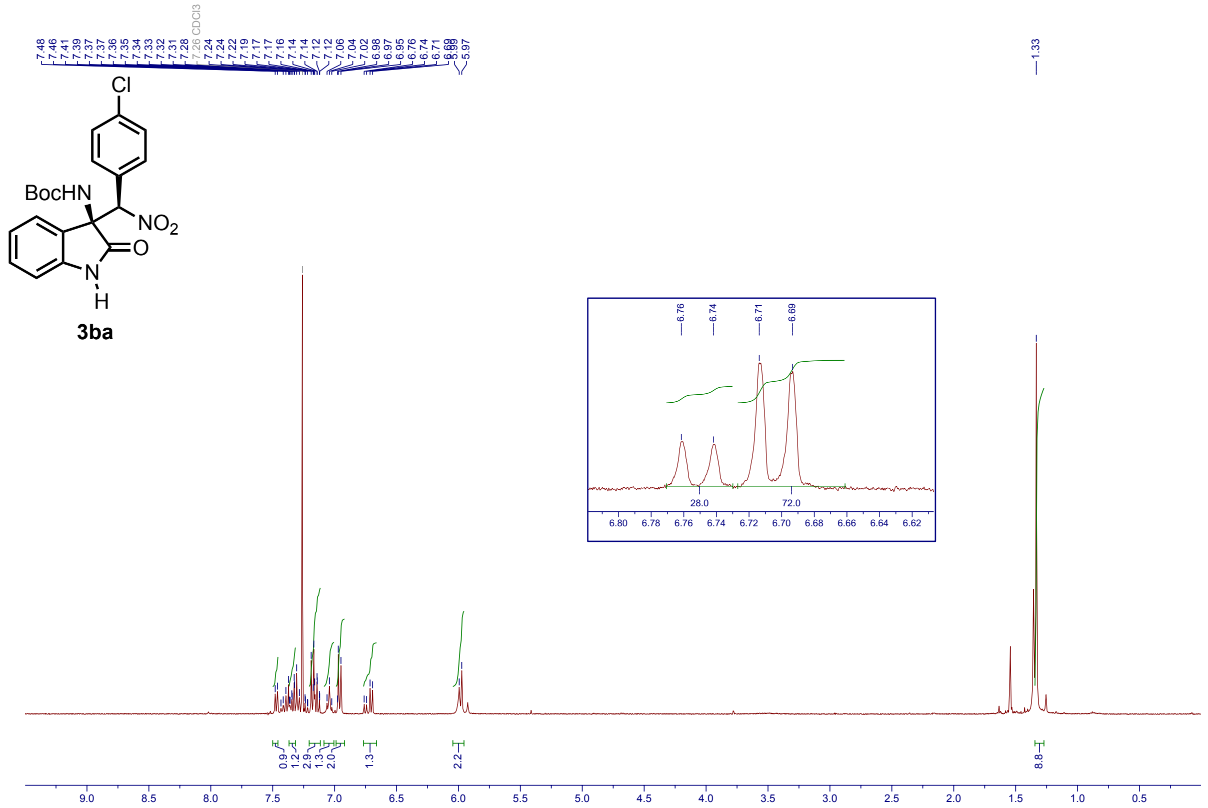

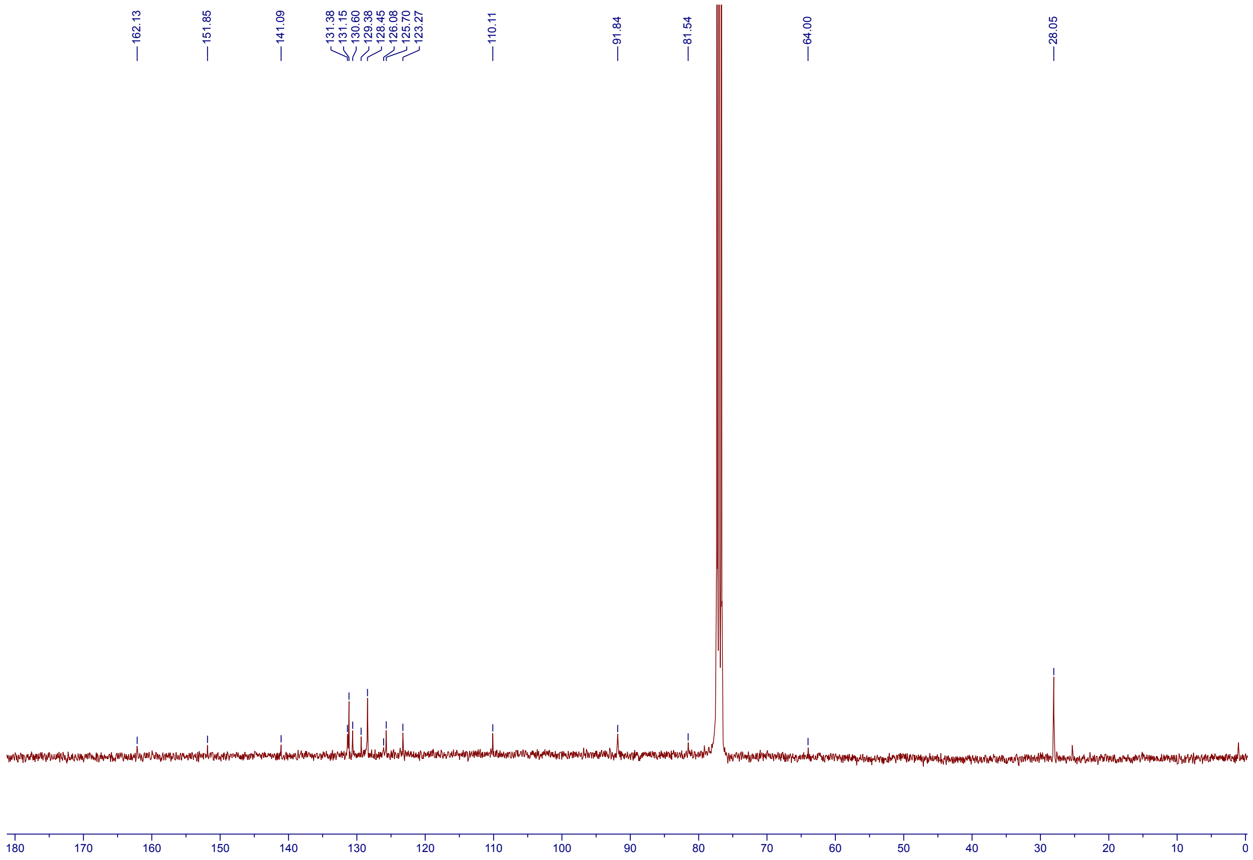

^{1}H-NMR (400 MHz), ^{13}C{^{1}H}-NMR (101 MHz) of *tert*-butyl ((*S*)-3-((*R*)-(4-chlorophenyl)(nitro)methyl)-1-methyl-2-oxoindolin-3-yl)carbamate **3ca**

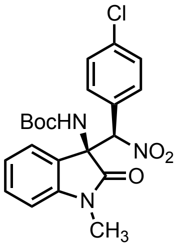

3ca

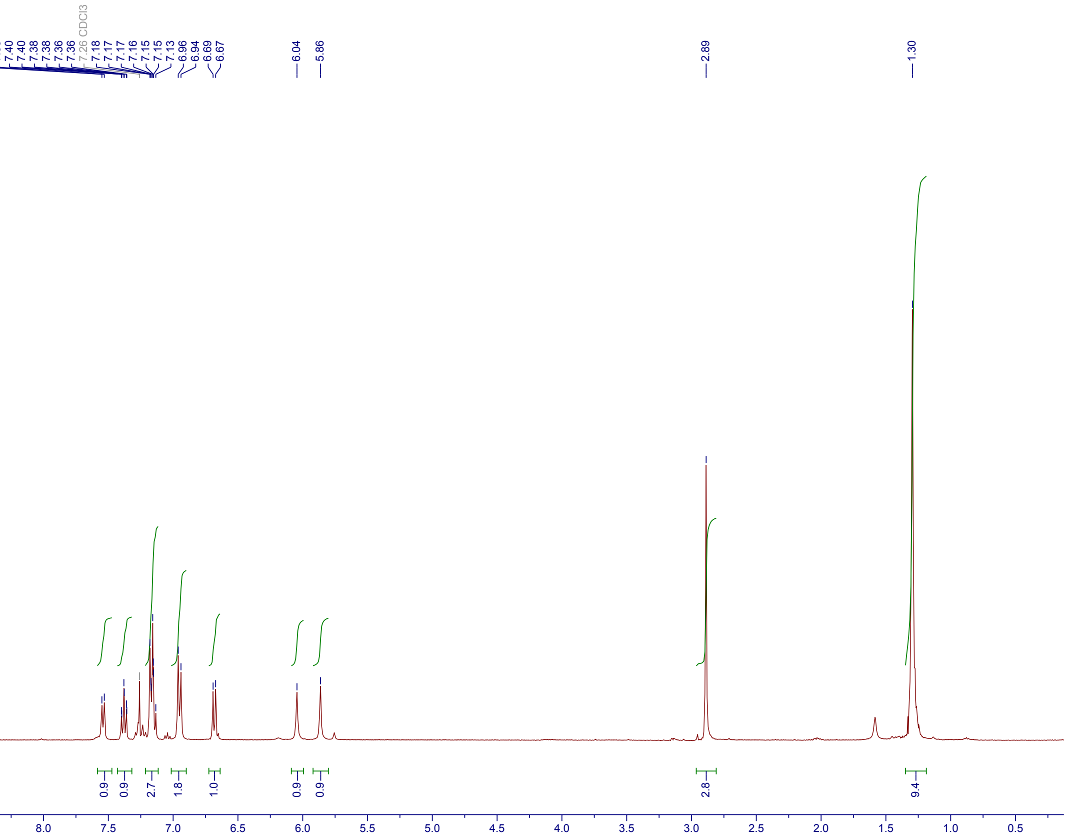

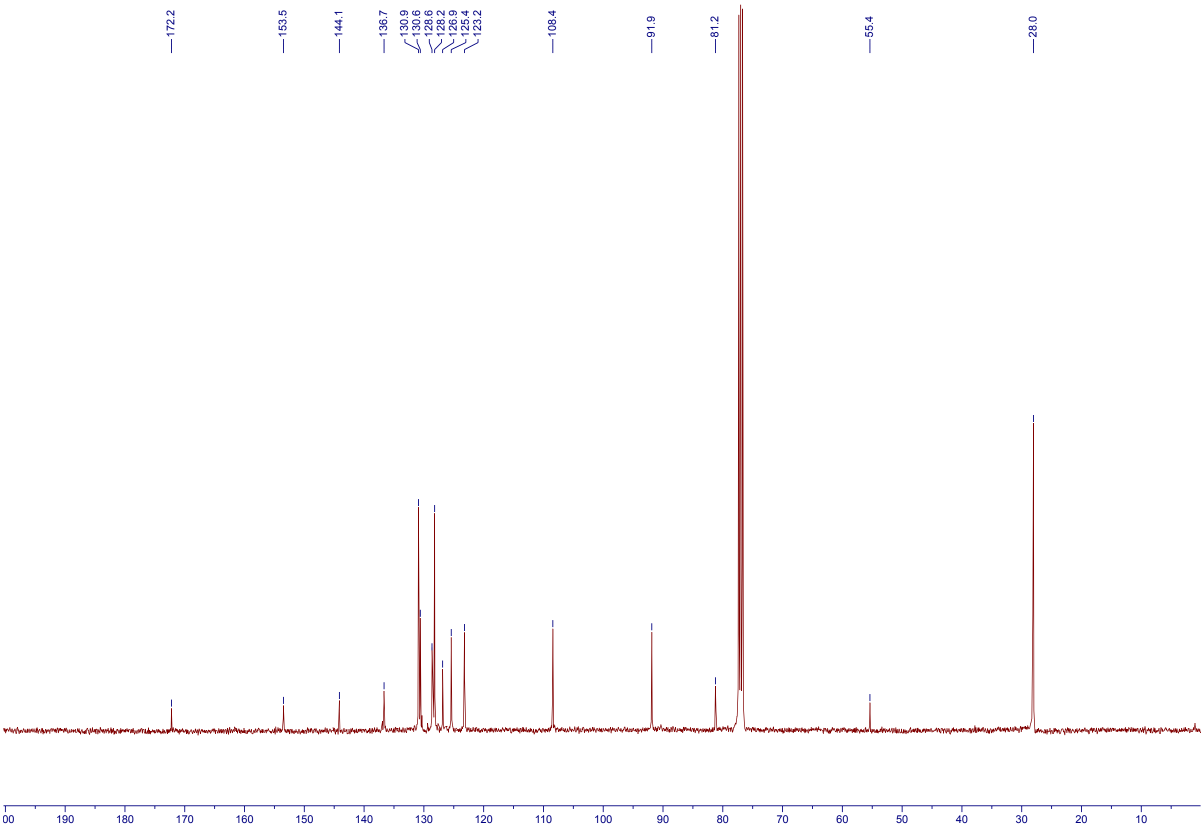

^{1}H-NMR (400 MHz), ^{13}C{^{1}H}-NMR (101 MHz) of *tert*-butyl ((*S*)-1-benzyl-5-chloro-3-((*R*)-(4-chlorophenyl)(nitro)methyl)-2-oxoindolin-3-yl)carbamate **3da**

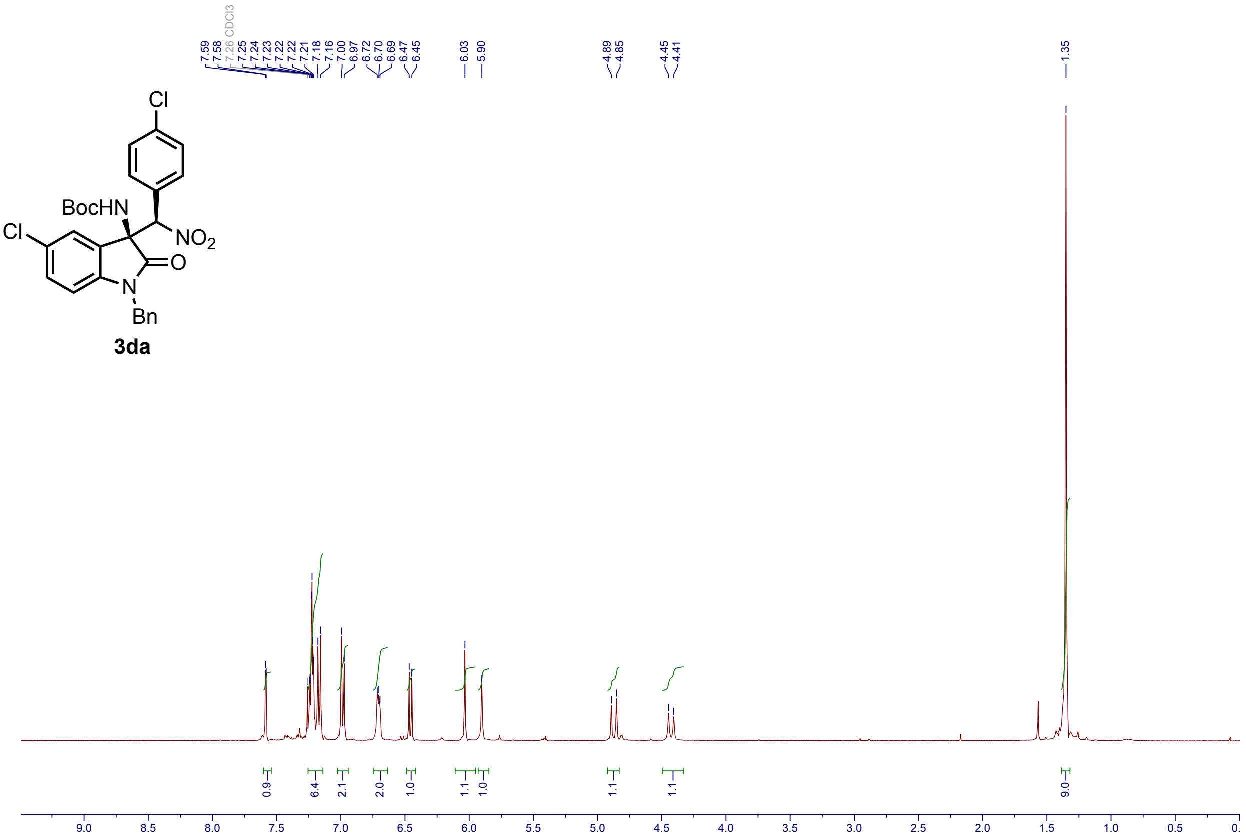

3da

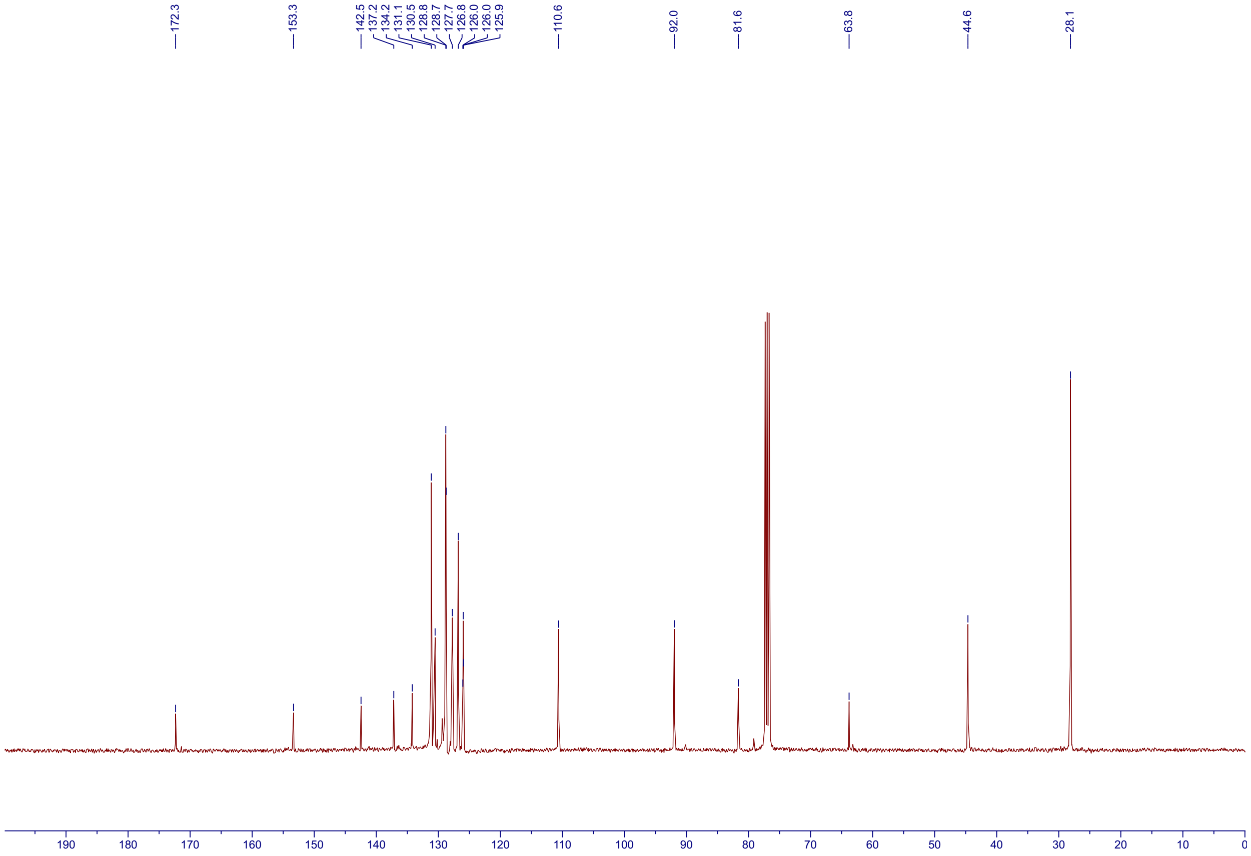

^{1}H-NMR (500 MHz), ^{13}C{^{1}H}-NMR (126 MHz) of *tert*-butyl ((*S*)-1-benzyl-3-((*R*)-(4-chlorophenyl)(nitro)methyl)-5-methoxy-2-oxoindolin-3-yl)carbamate **3ea**

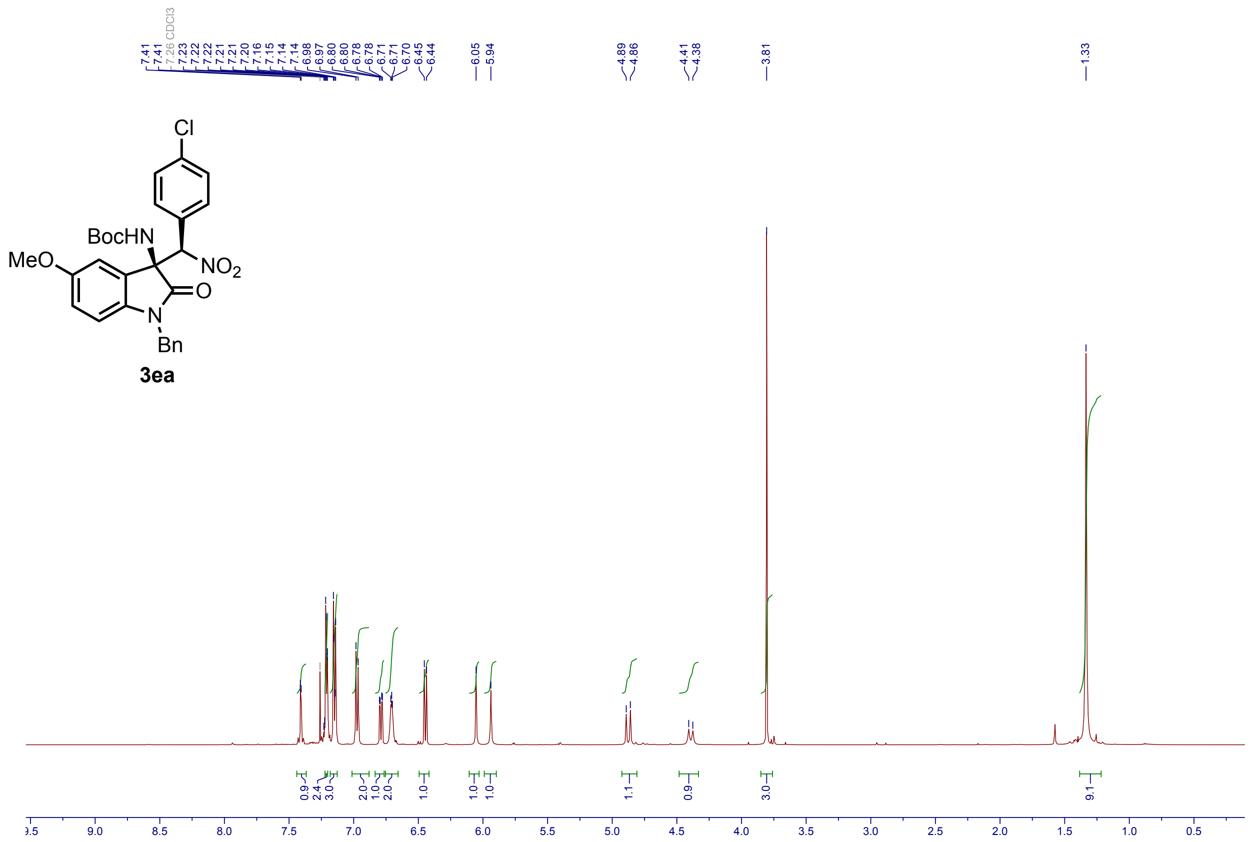

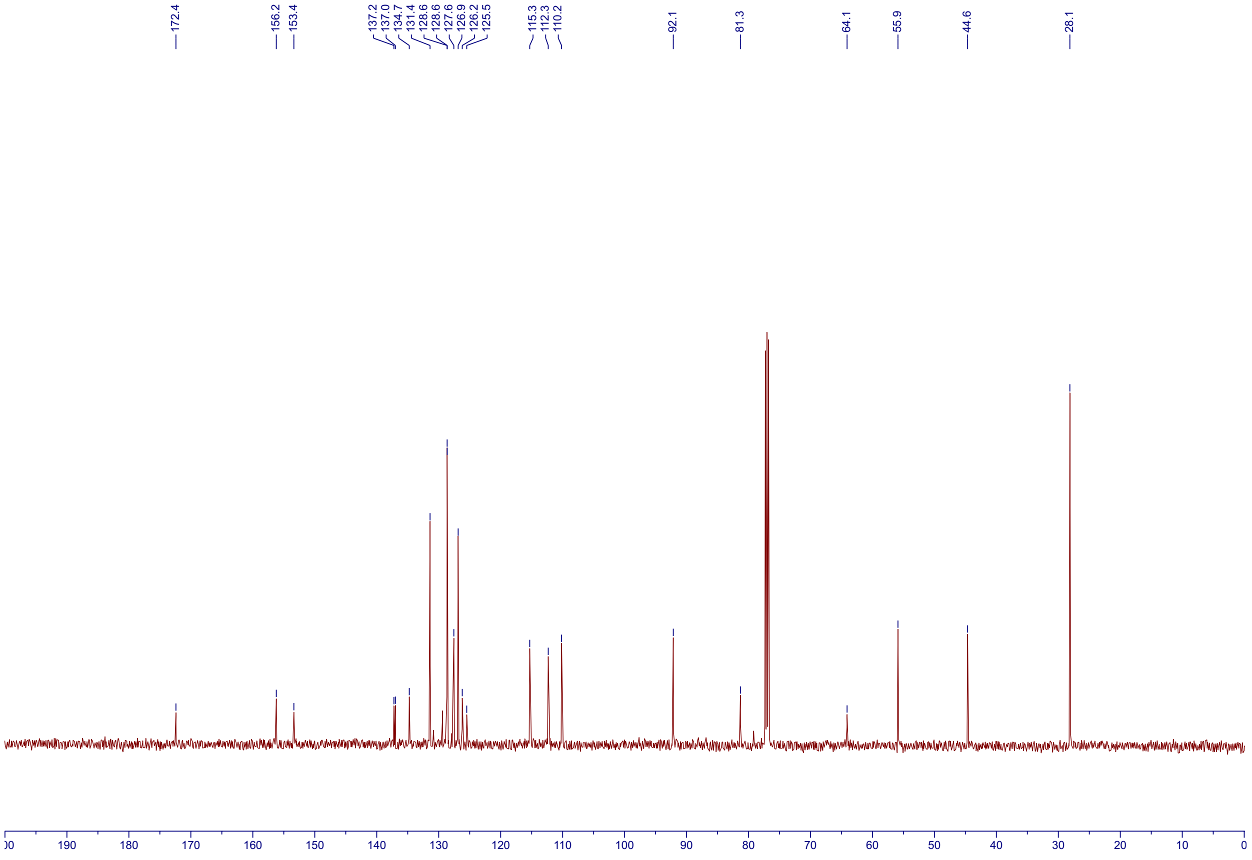

^{1}H-NMR (400 MHz), ^{13}C{^{1}H}-NMR (101 MHz) of *tert*-butyl ((*S*)-1-benzyl-6-chloro-3-((*R*)-(4-chlorophenyl)(nitro)methyl)-2-oxoindolin-3-yl)carbamate **3fa**

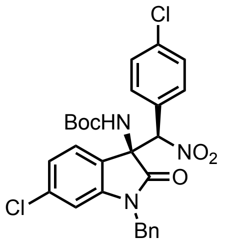

3fa

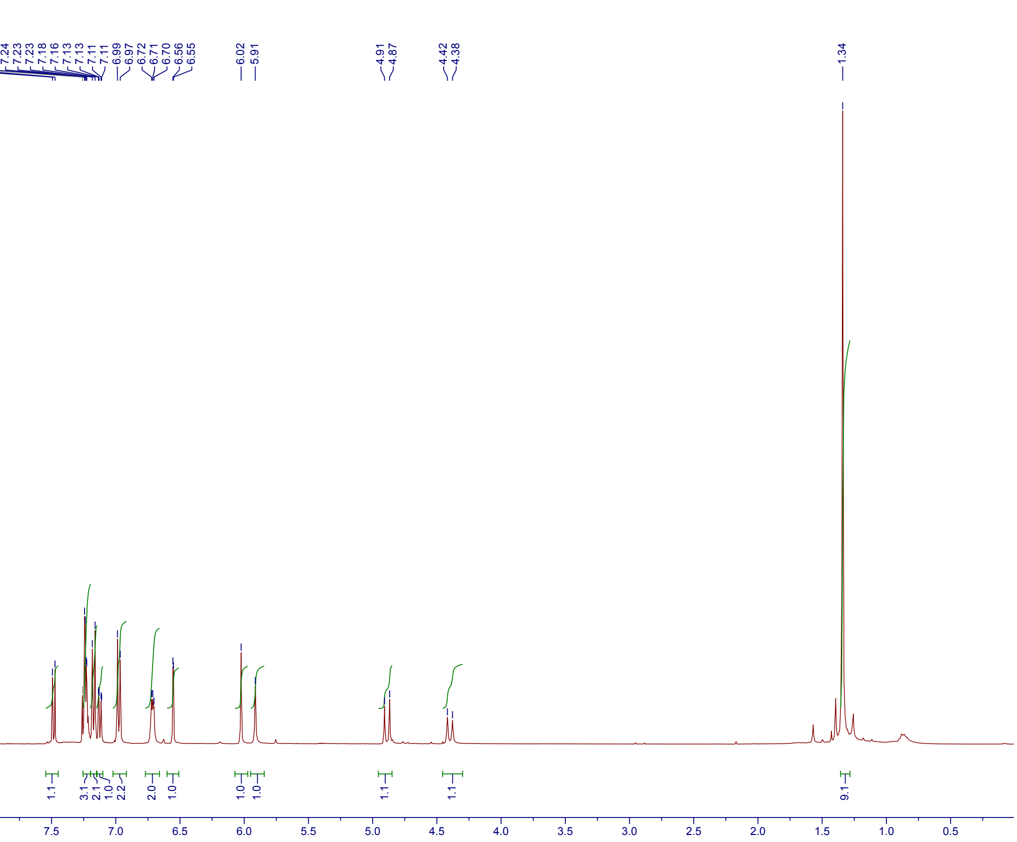

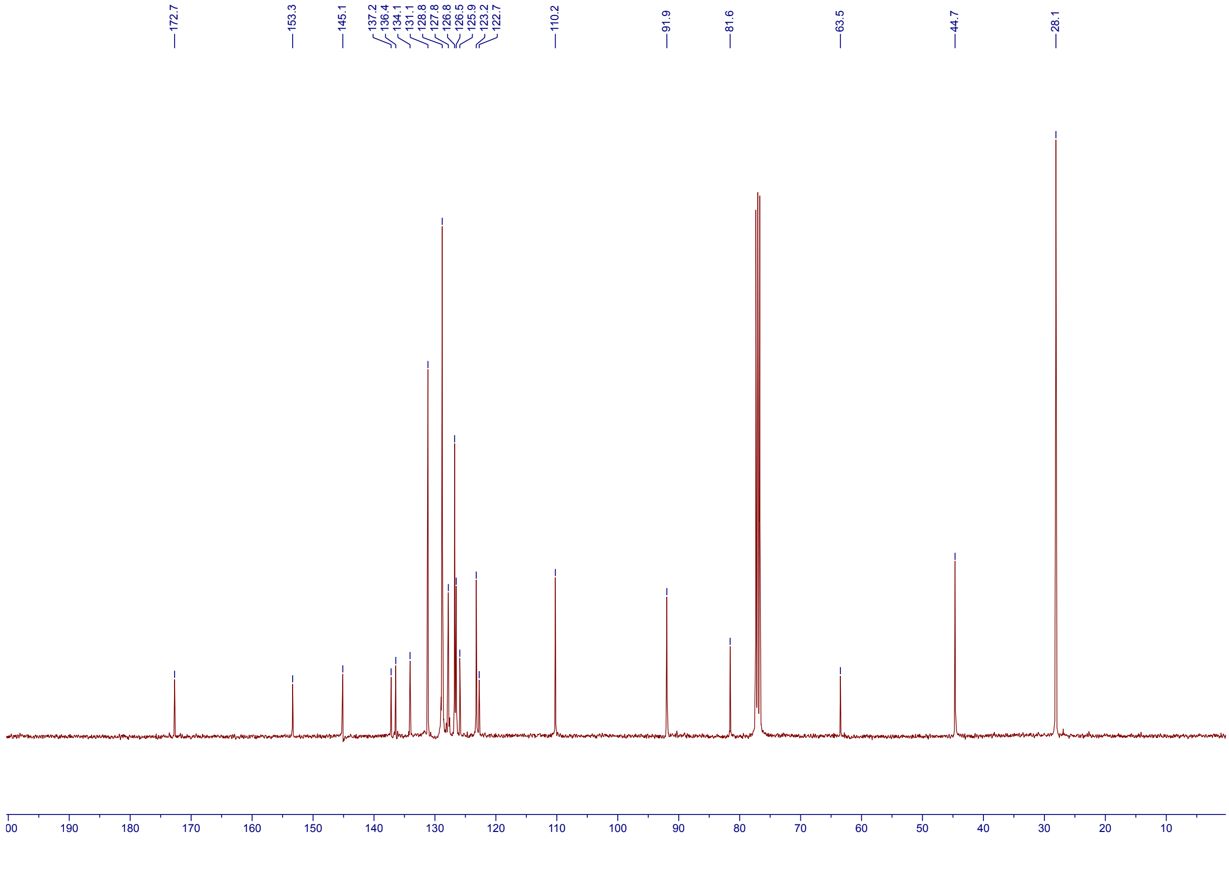

^{1}H-NMR (400 MHz), ^{13}C{^{1}H}-NMR (101 MHz) of *tert*-butyl ((*S*)-1-benzyl-3-((*R*)-(4-chlorophenyl)(nitro)methyl)-6-methoxy-2-oxoindolin-3-yl)carbamate **3ga**

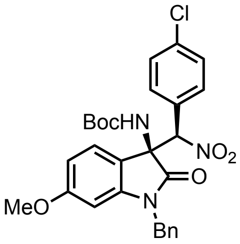

3ga

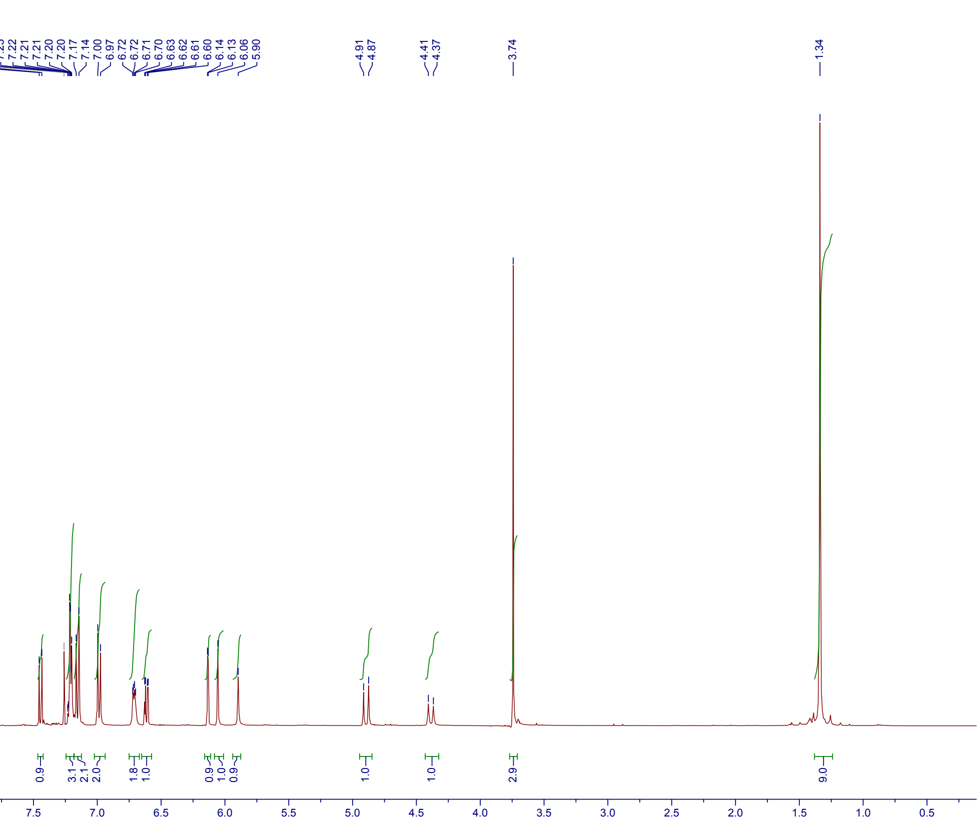

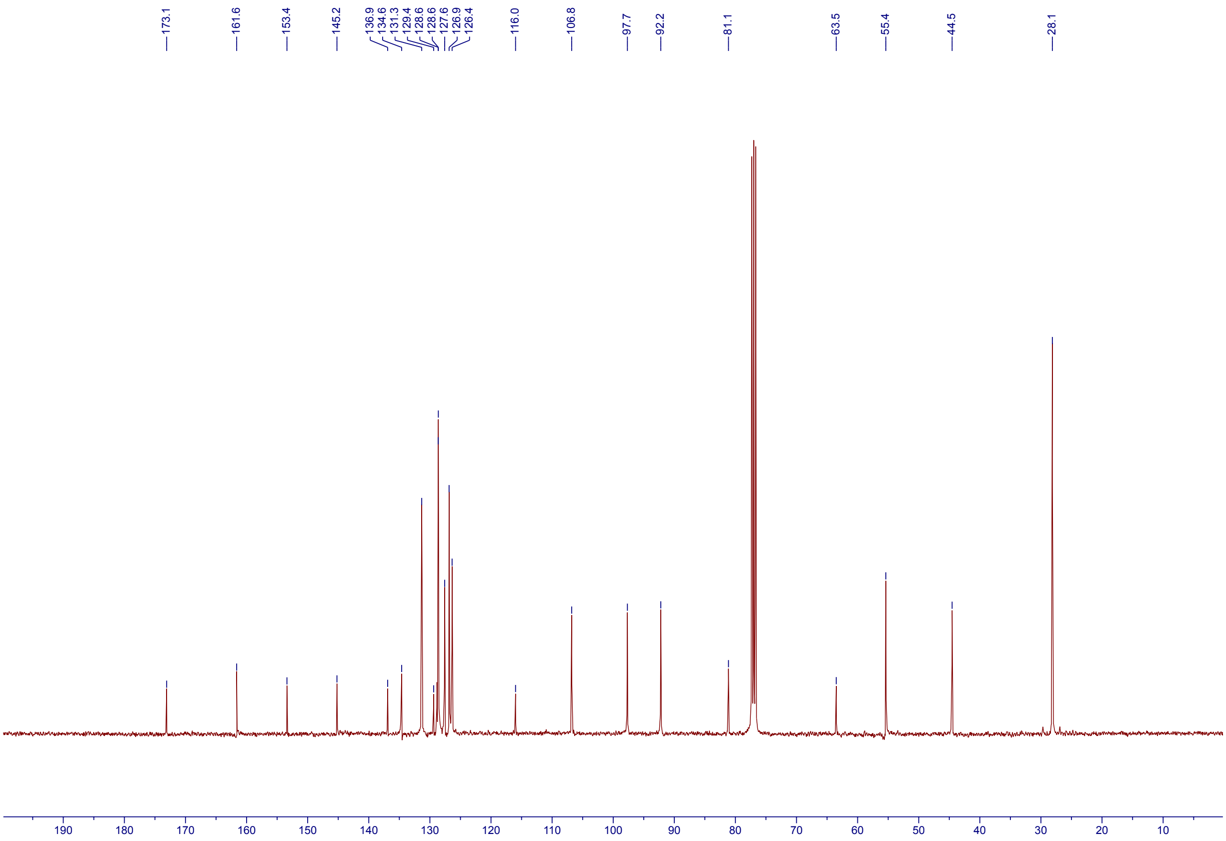

^{1}H-NMR (400 MHz), ^{13}C{^{1}H}-NMR (101 MHz) of *tert*-butyl ((*S*)-3-((*R*)-nitro(phenyl)methyl)-2-oxoindolin-3-yl)carbamate **3bg**

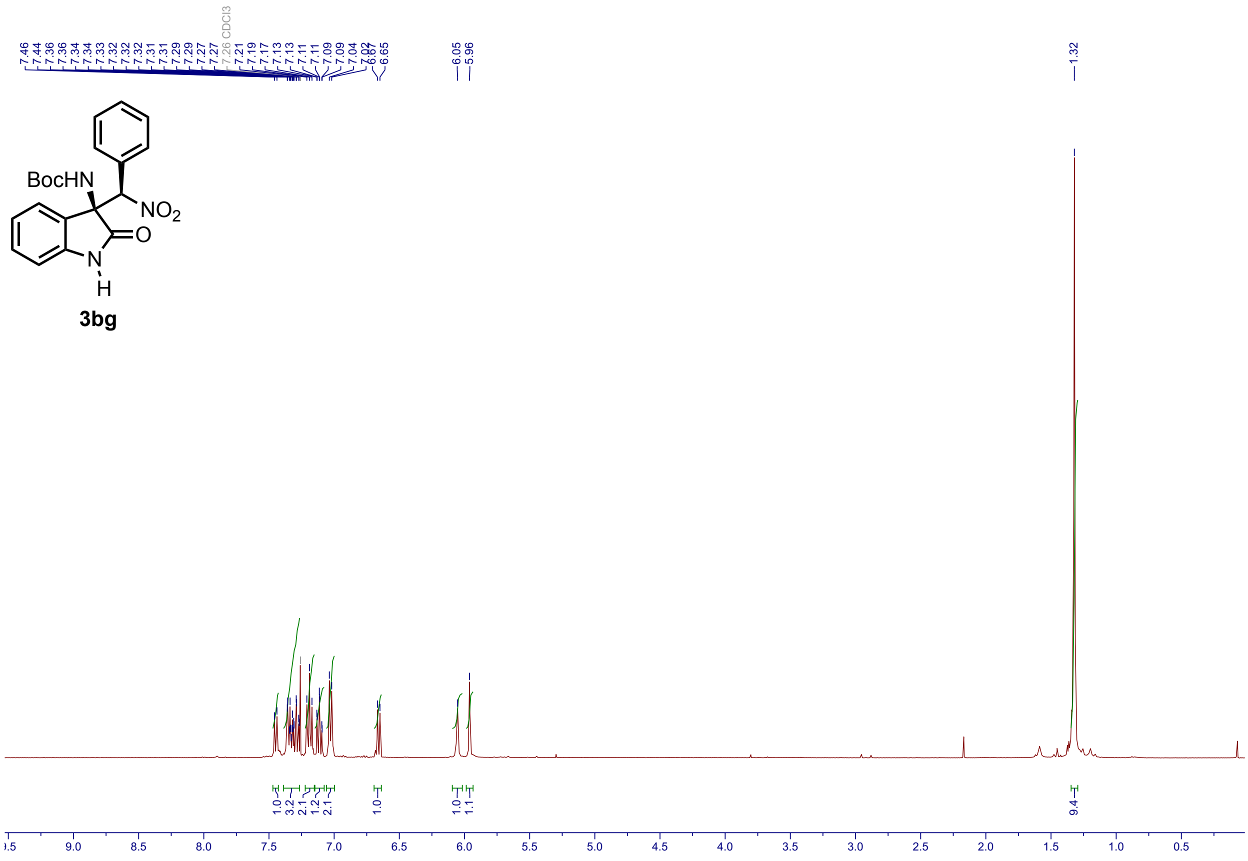

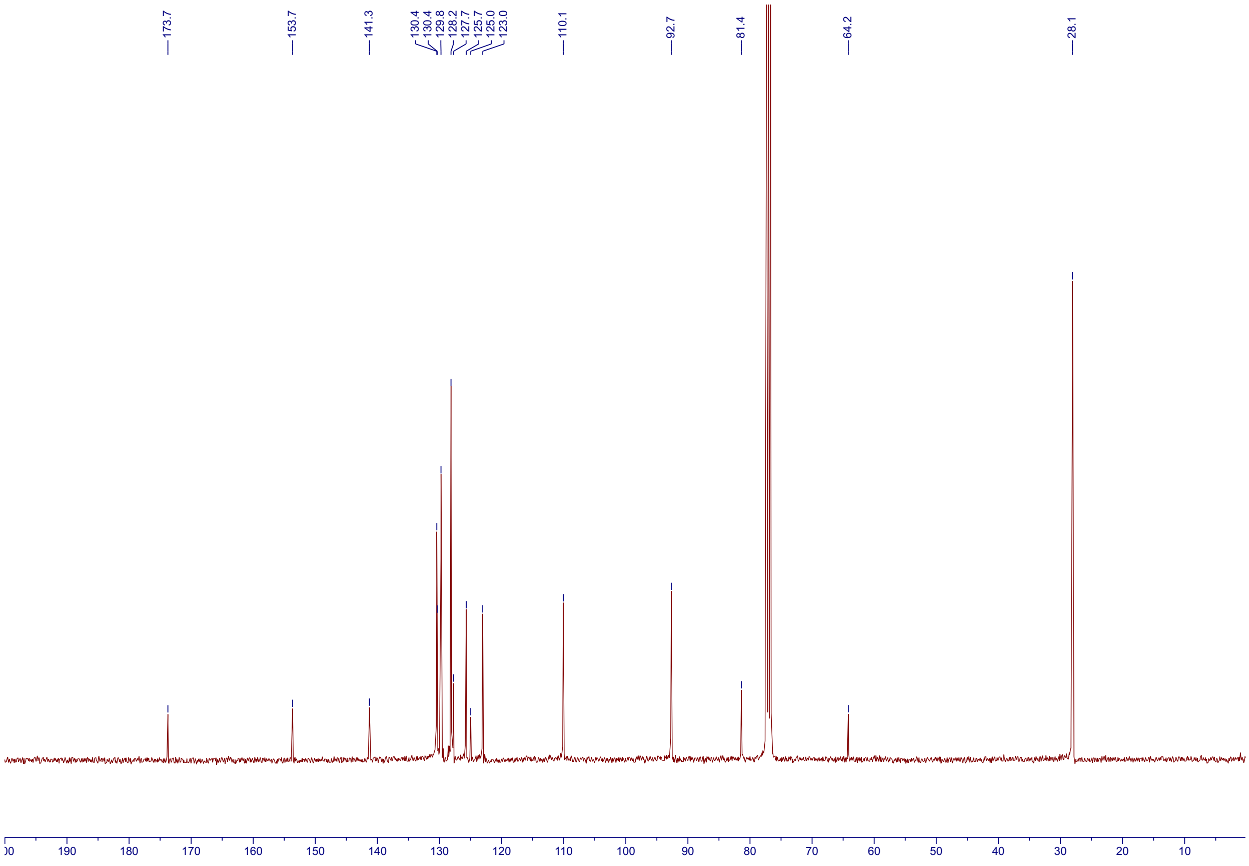

^{1}H-NMR (500 MHz), ^{13}C{^{1}H}-NMR (126 MHz) of *tert*-butyl (*R*)-(1-benzyl-3-(nitromethyl)-2-oxoindolin-3-yl)carbamate **3ah**

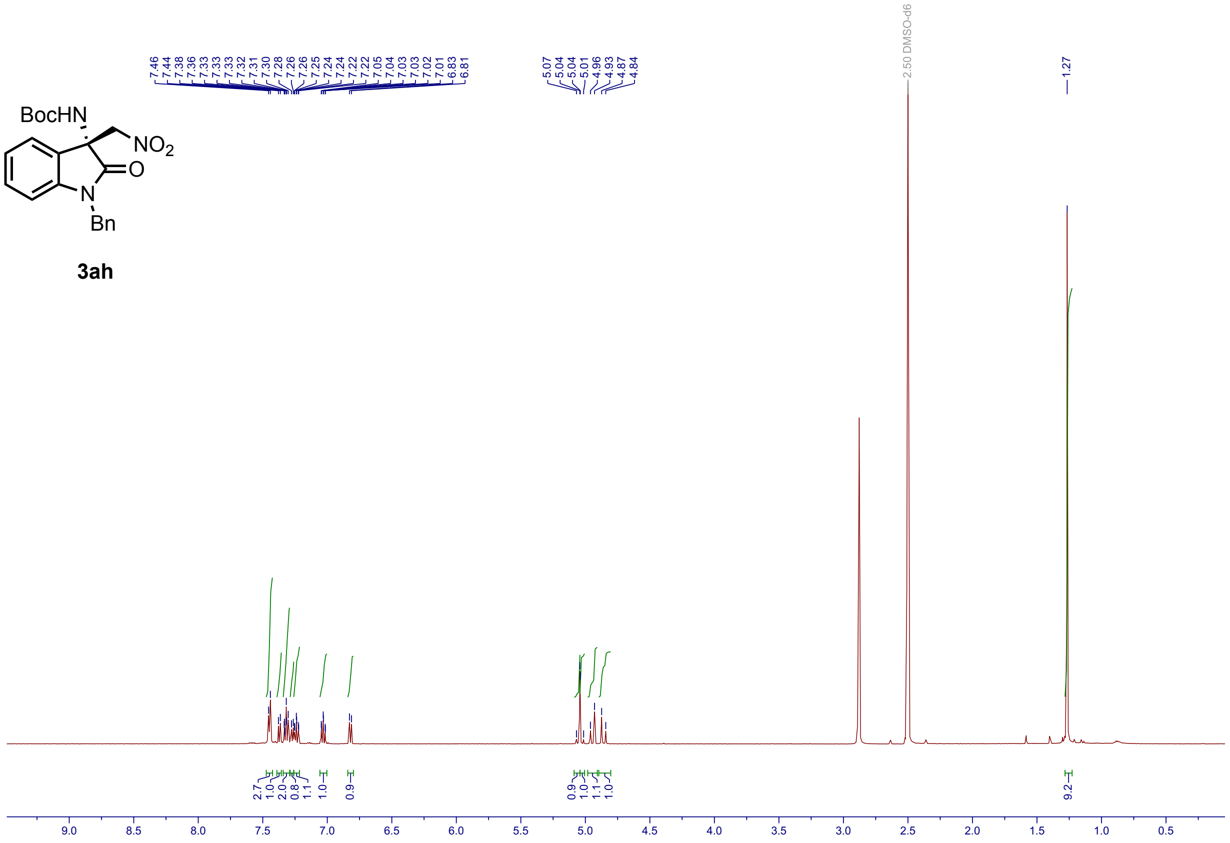

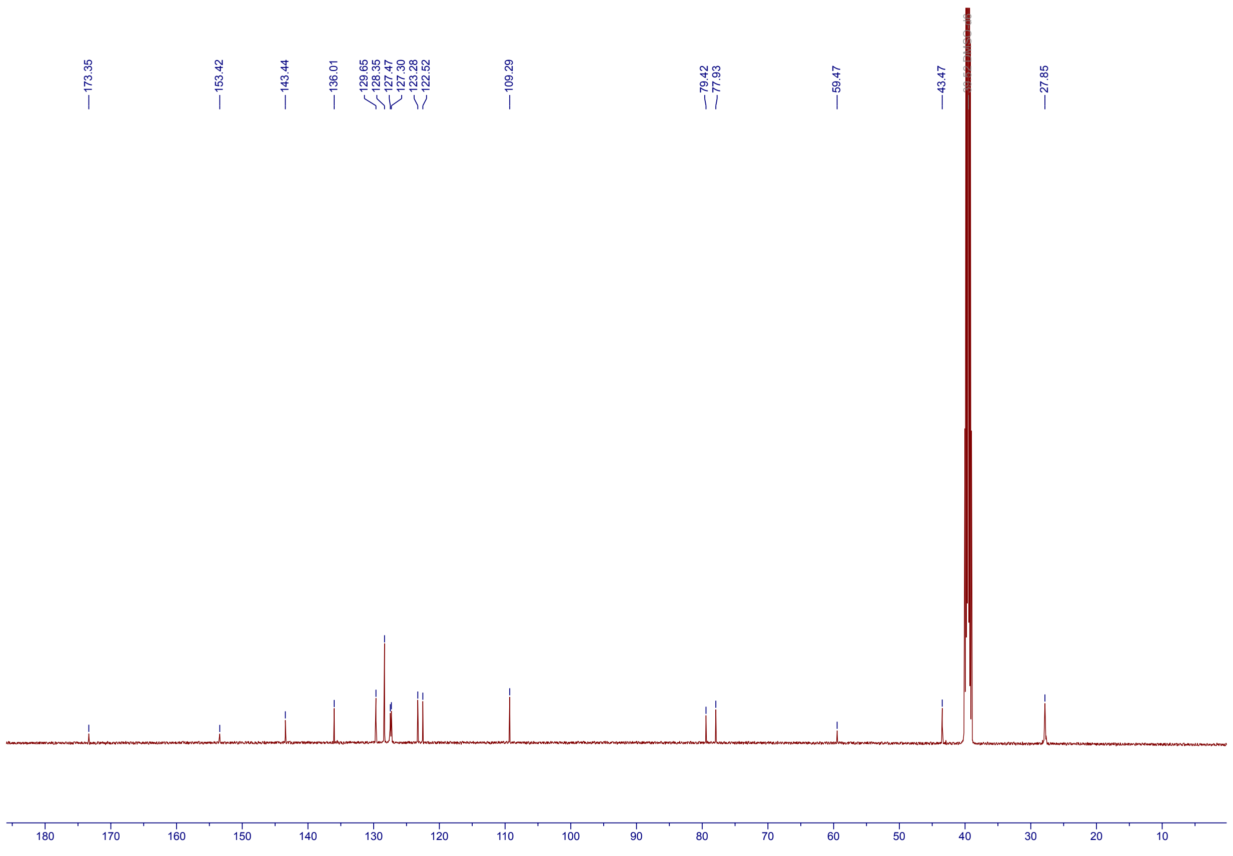

HPLC Chromatograms of Oxindoles 3

HPLC Chromatograms of ***rac*-3aa** and **3aa** under Homogeneous Conditions and Heterogeneous Conditions

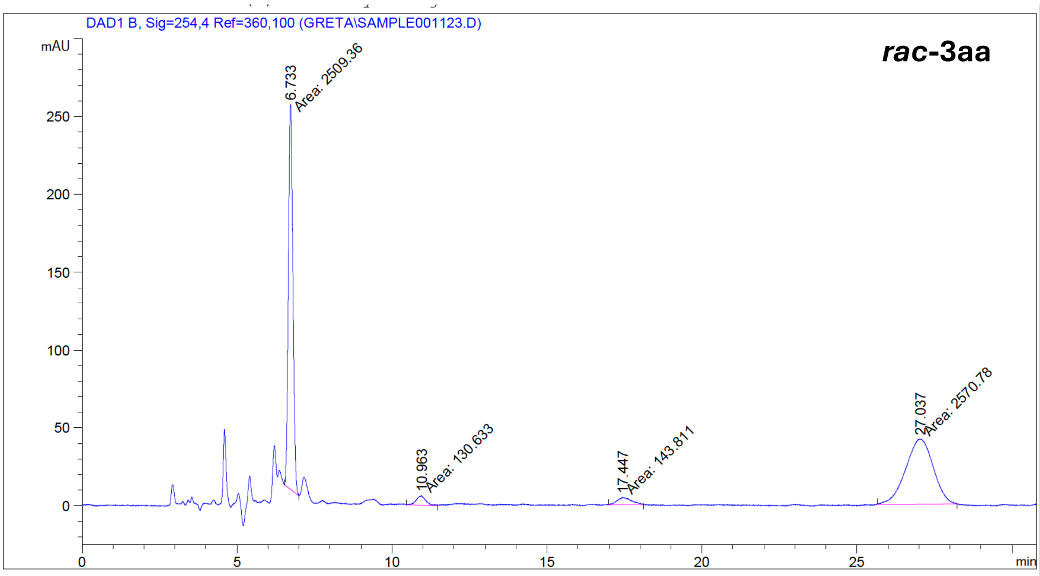

Signal 1: DAD1 B, Sig=254,4 Ref=360,100

Peak #	RetTime [min]	Type	Width [min]	Area [mAU*s]	Height [mAU]	Area %
1	6.733	MM	0.1690	2509.35913	247.40598	46.8637
2	10.963	MM	0.3584	130.63274	6.07554	2.4396
3	17.447	MM	0.5232	143.81065	4.58092	2.6857
4	27.037	MM	1.0246	2570.78223	41.81716	48.0109

Totals : 5354.58475 299.87960

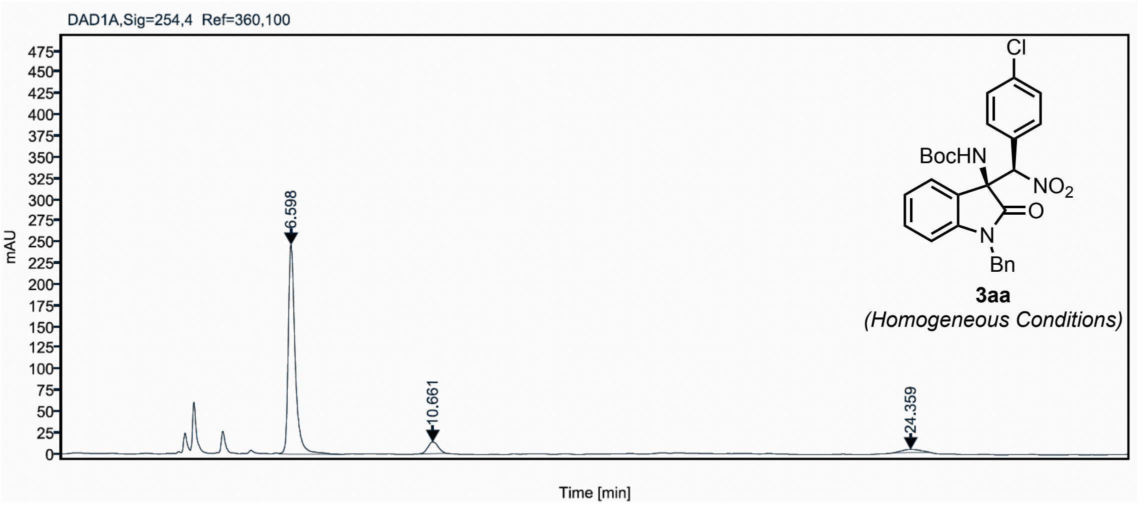

Signal: DAD1A,Sig=254,4 Ref=360,100

RT [min]	Type	Width [min]	Area	Height	Area%	Name
6.598	VB	1.7681	3393.2424	247.0829	88.9181	
10.661	MM m	0.8507	286.6408	13.9311	7.5113	
24.359	MM m	1.0290	136.2612	3.7543	3.5707	
		Sum	3816.1445			

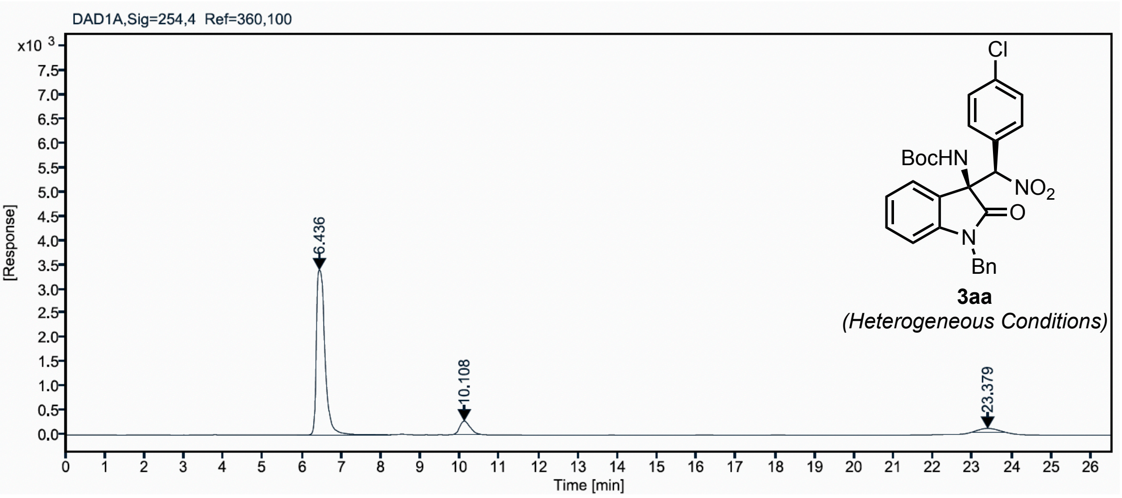

Signal: DAD1A,Sig=254,4 Ref=360,100

RT [min]	Type	Width [min]	Area	Height	Area%	Name
6.436	MM m	2.3649	51816.2279	3393.8554	86.3789	
10.108	MM m	0.8337	5223.6465	280.5485	8.7079	
23.379	MM m	0.9417	2947.2847	87.0622	4.9132	
		**Sum**	**59987.1591**			

HPLC Chromatograms of ***rac*-3ab** and **3ab** under Homogeneous Conditions

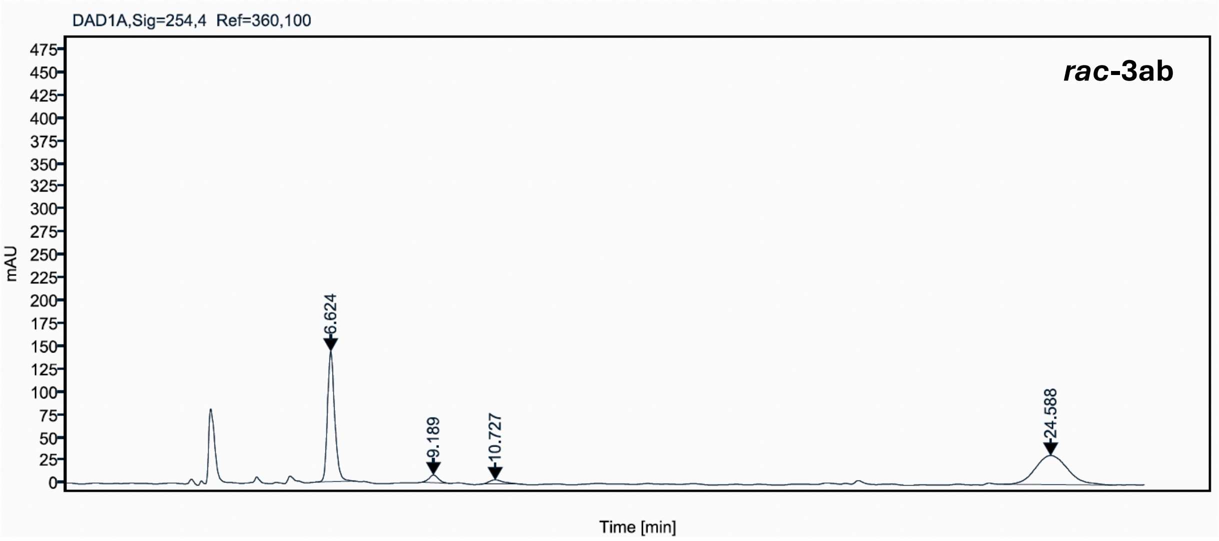

Signal: DAD1A,Sig=254,4 Ref=360,100

RT [min]	Type	Width [min]	Area	Height	Area%	Name
6.624	MM m	0.9123	1780.7041	142.5490	46.3368	
9.189	MM m	0.6895	138.0714	8.4347	3.5928	
10.727	MM m	1.0184	129.0270	4.7765	3.3575	
24.588	BB	2.6933	1795.1602	31.8971	46.7129	
		**Sum**	**3842.9627**			

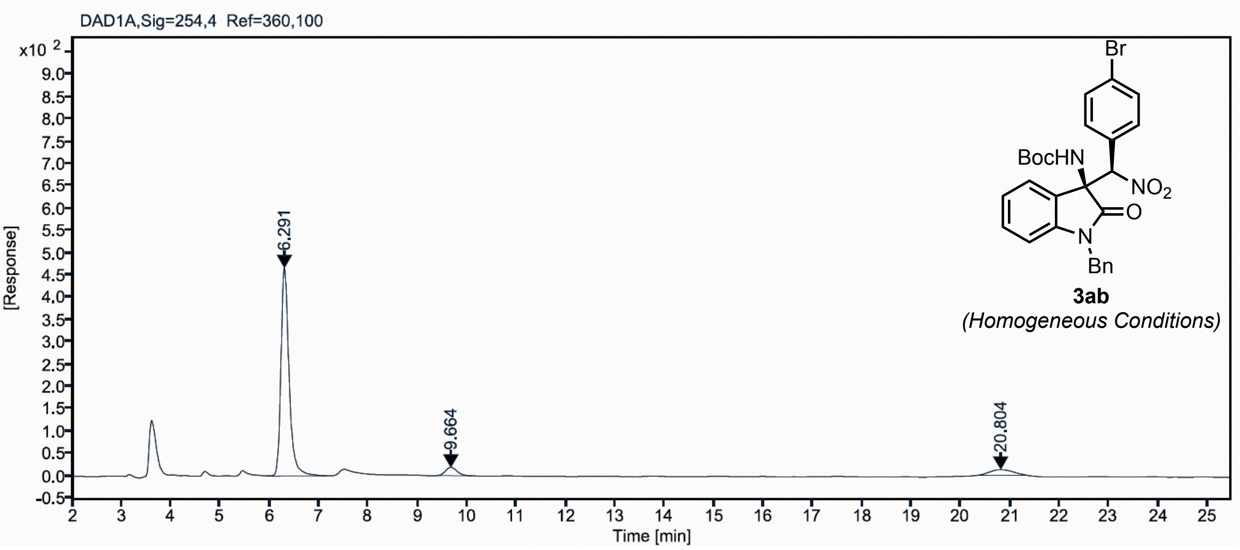

Signal: DAD1A,Sig=254,4 Ref=360,100

RT [min]	Type	Width [min]	Area	Height	Area%	Name
6.291	VB	1.1963	5360.1732	466.4323	88.0559	
9.664	MM m	0.6806	303.8992	18.6263	4.9924	
20.804	MM m	1.0126	423.1696	12.8770	6.9517	
		**Sum**	**6087.2420**			

HPLC Chromatograms of ***rac*-3ac** and **3ac** under Homogeneous Conditions, Heterogeneous Conditions and after the Sixth Reuse of Heterogeneous Catalyst

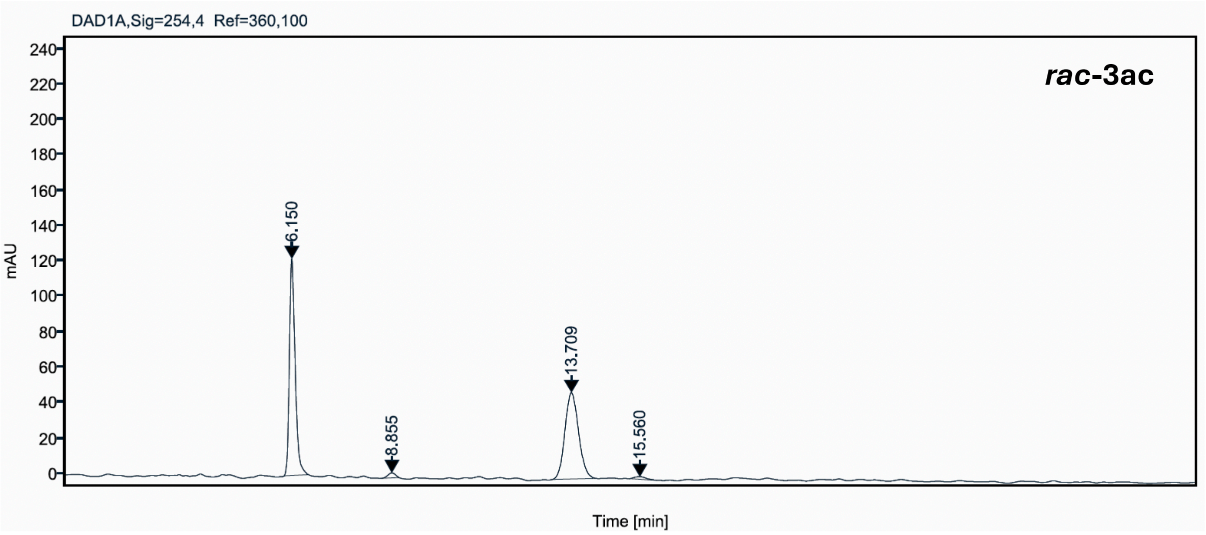

Signal: DAD1A,Sig=254,4 Ref=360,100

RT [min]	Type	Width [min]	Area	Height	Area%	Name
6.150	MM m	0.6592	1334.9992	122.5101	50.2098	
8.855	BM m	0.4962	39.2566	2.8130	1.4765	
13.709	MM m	1.1877	1252.3824	48.8544	47.1025	
15.560	MM m	0.7422	32.2040	1.5213	1.2112	
		**Sum**	**2658.8422**			

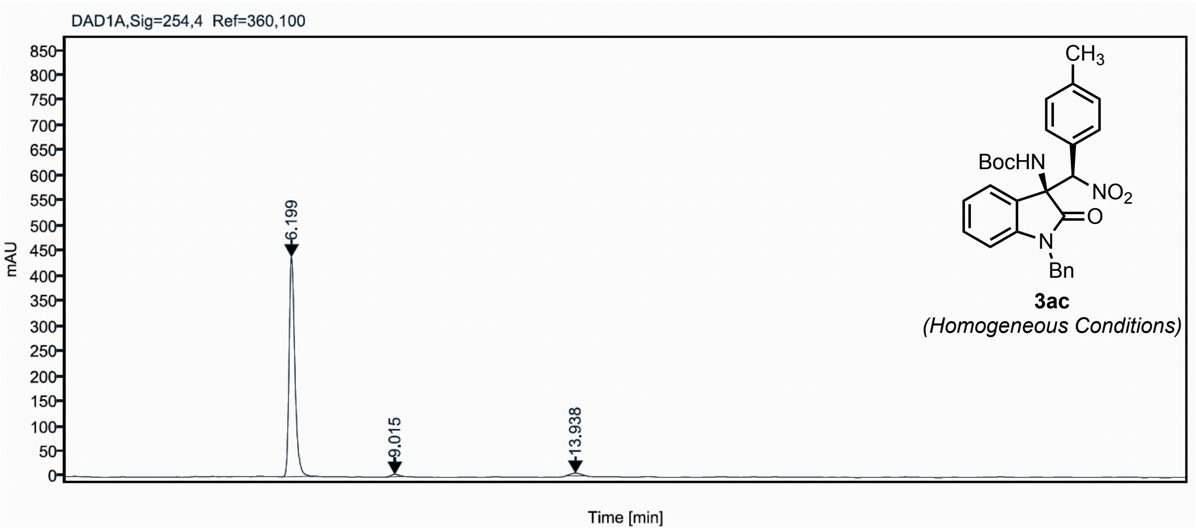

Signal: DAD1A,Sig=254,4 Ref=360,100

RT [min]	Type	Width [min]	Area	Height	Area%	Name
6.199	BB	0.9003	4766.0786	438.7859	96.3760	
9.015	MM m	0.4823	70.3589	4.8931	1.4227	
13.938	MM m	0.5549	108.8563	5.5494	2.2012	
		**Sum**	**4945.2938**			

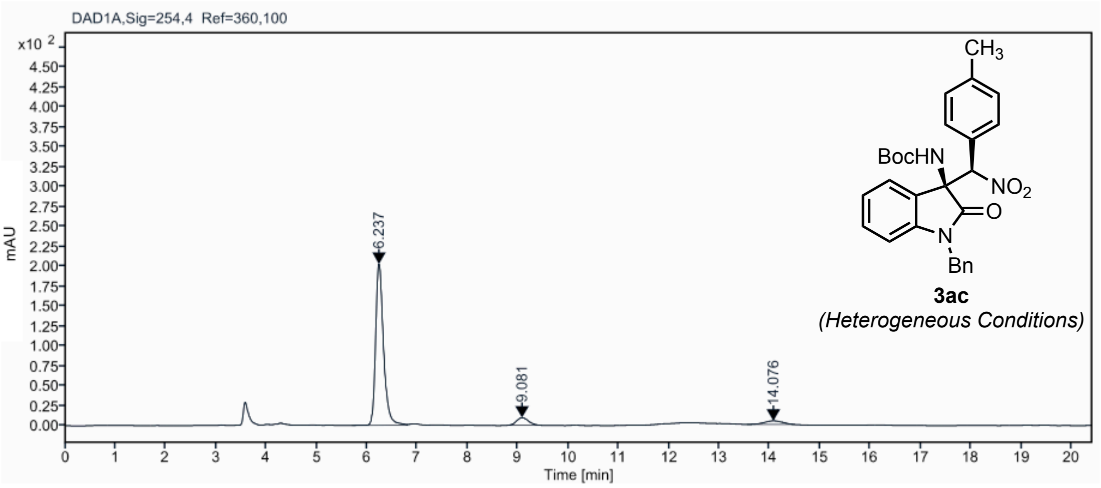

Signal: DAD1A,Sig=254,4 Ref=360,100

RT [min]	Type	Width [min]	Area	Height	Area%	Name
6.237	BV	1.1050	2207.6949	202.4033	89.5468	
9.081	MM m	0.5491	143.3963	9.2327	5.8163	
14.076	BM m	0.9520	114.3182	4.5017	4.6369	
		**Sum**	**2465.4095**			

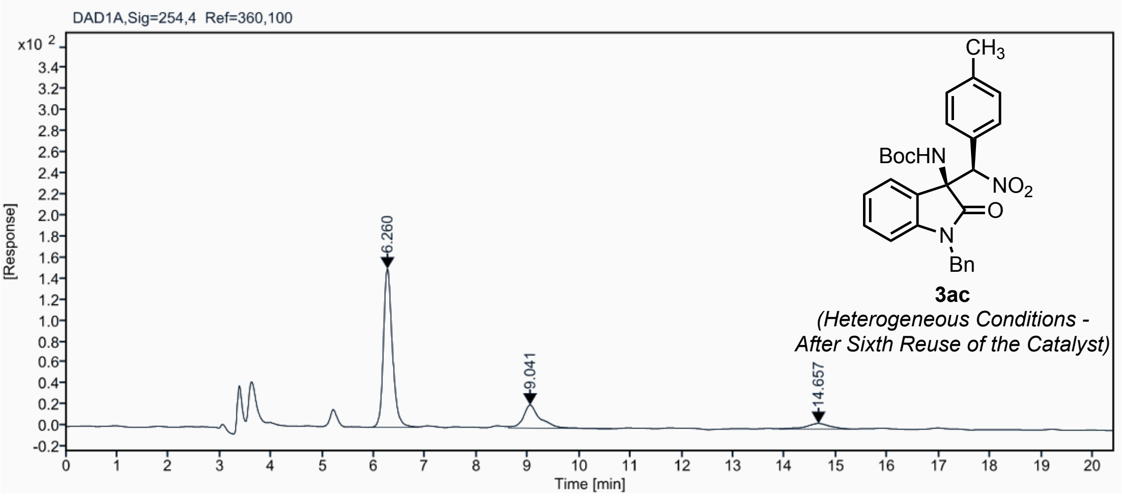

Signal: DAD1A,Sig=254,4 Ref=360,100

RT [min]	Type	Width [min]	Area	Height	Area%	Name
6.260	BV	0.9790	1827.9938	150.8640	73.3678	
9.041	VB	1.9930	500.3798	21.9249	20.0831	
14.657	VB	1.3764	163.1747	5.1537	6.5491	
		**Sum**	**2491.5482**			

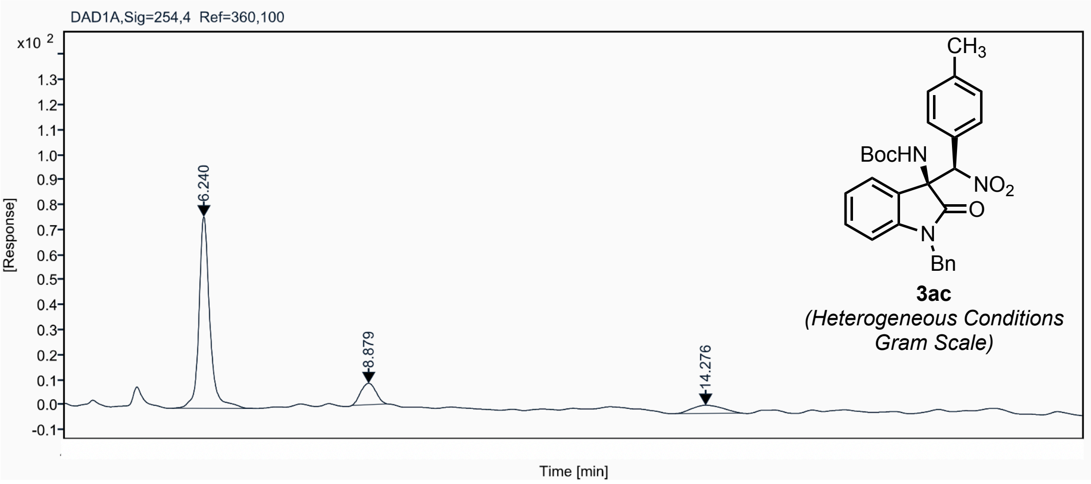

Signal: DAD1A,Sig=254,4 Ref=360,100

RT [min]	Type	Width [min]	Area	Height	Area%	Name
6.240	MB m	1.3444	927.3880	76.5899	81.7521	
8.879	MM m	0.5708	145.2707	8.5438	12.8061	
14.276	MM m	0.7354	61.7319	2.3087	5.4419	
		**Sum**	**1134.3906**			

HPLC Chromatograms of ***rac*-3ad** and **3ad** under Homogeneous Conditions

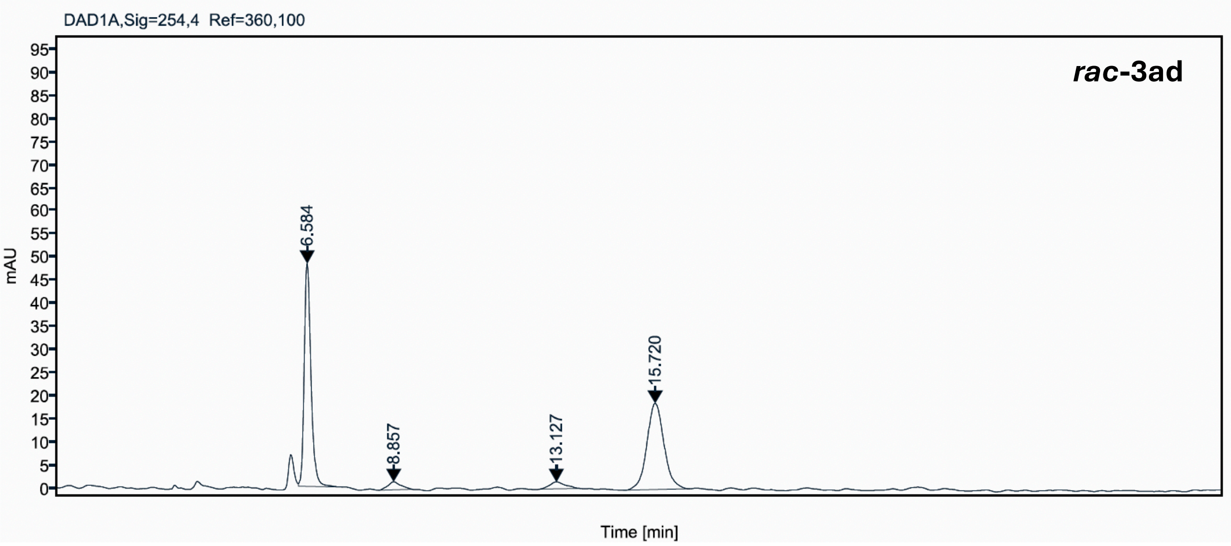

Signal:	DAD1A,Sig=254,4 Ref=360,100					
RT [min]	Type	Width [min]	Area	Height	Area%	Name
6.584	MM m	1.1538	590.7213	48.1262	46.9453	
8.857	BM m	0.8957	41.1209	1.7442	3.2679	
13.127	MM m	1.0575	43.4045	1.5170	3.4494	
15.720	BB	1.7462	583.0706	18.6102	46.3373	
		Sum	1258.3172			

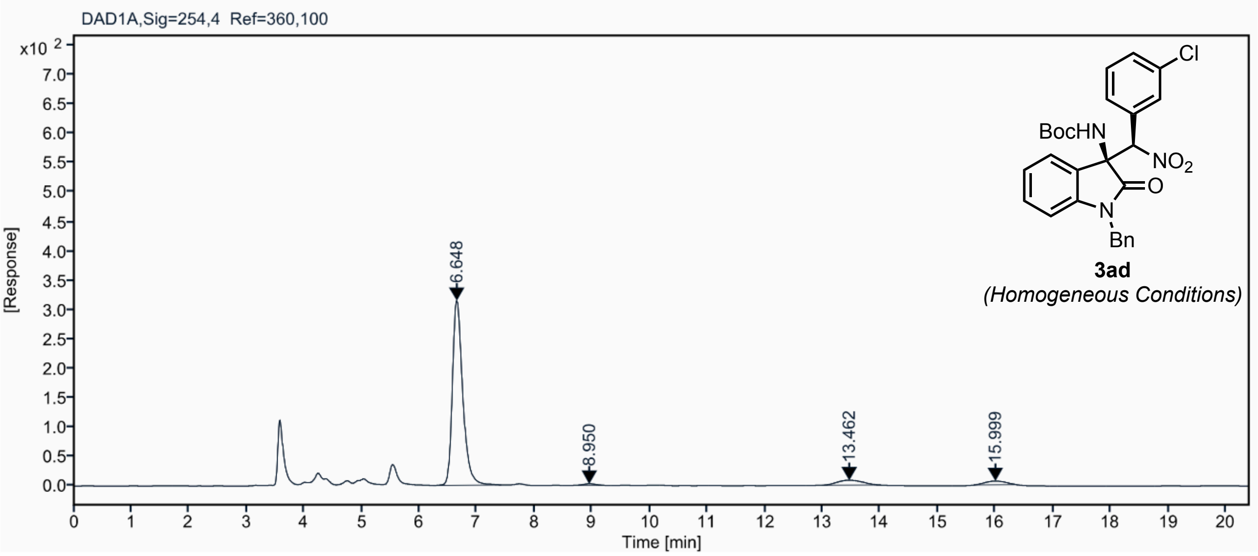

Signal:	DAD1A,Sig=254,4 Ref=360,100					
RT [min]	Type	Width [min]	Area	Height	Area%	Name
6.648	MM m	1.1734	3946.6479	314.4412	89.5065	
8.950	MM m	0.4383	34.0559	2.4766	0.7724	
13.462	MM m	1.0079	261.7405	8.4872	5.9360	
15.999	MM m	0.7405	166.8966	6.6432	3.7851	
		Sum	4409.3409			

HPLC Chromatograms of ***rac*-3ae** and **3ae** under Homogeneous Conditions

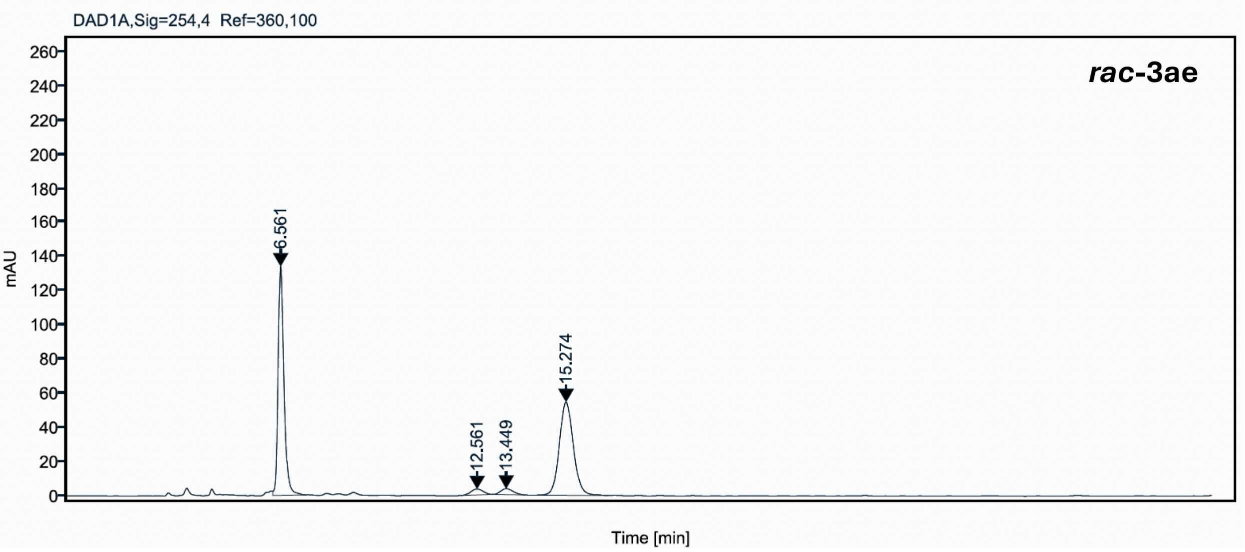

Signal: DAD1A,Sig=254,4 Ref=360,100

RT [min]	Type	Width [min]	Area	Height	Area%	Name
6.561	VB	1.0106	1663.0571	134.0922	47.7020	
12.561	MM m	0.9834	92.1593	3.5161	2.6434	
13.449	MM m	0.9867	93.7568	3.5480	2.6893	
15.274	BB	2.2933	1637.3699	54.6868	46.9653	
		**Sum**	**3486.3432**			

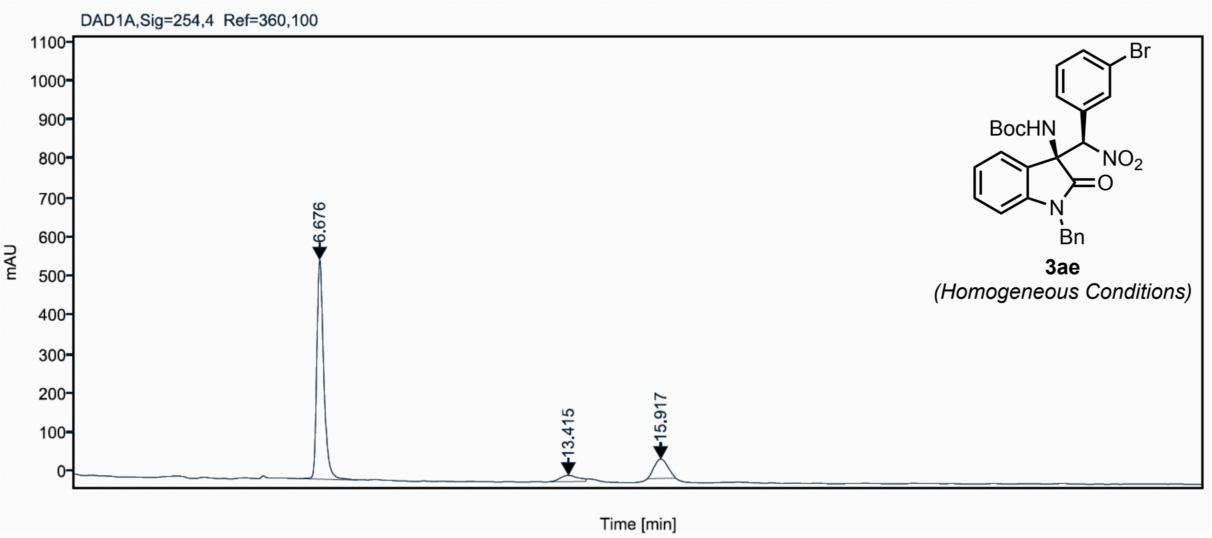

Signal: DAD1A,Sig=254,4 Ref=360,100

RT [min]	Type	Width [min]	Area	Height	Area%	Name
6.676	BV	1.5654	7310.0759	561.9714	80.1304	
13.415	MM m	0.9862	561.4415	16.3022	6.1543	
15.917	MM m	0.7709	1251.2068	49.4861	13.7153	
		**Sum**	**9122.7242**			

HPLC Chromatograms of ***rac*-3af** and **3af** under Homogeneous Conditions

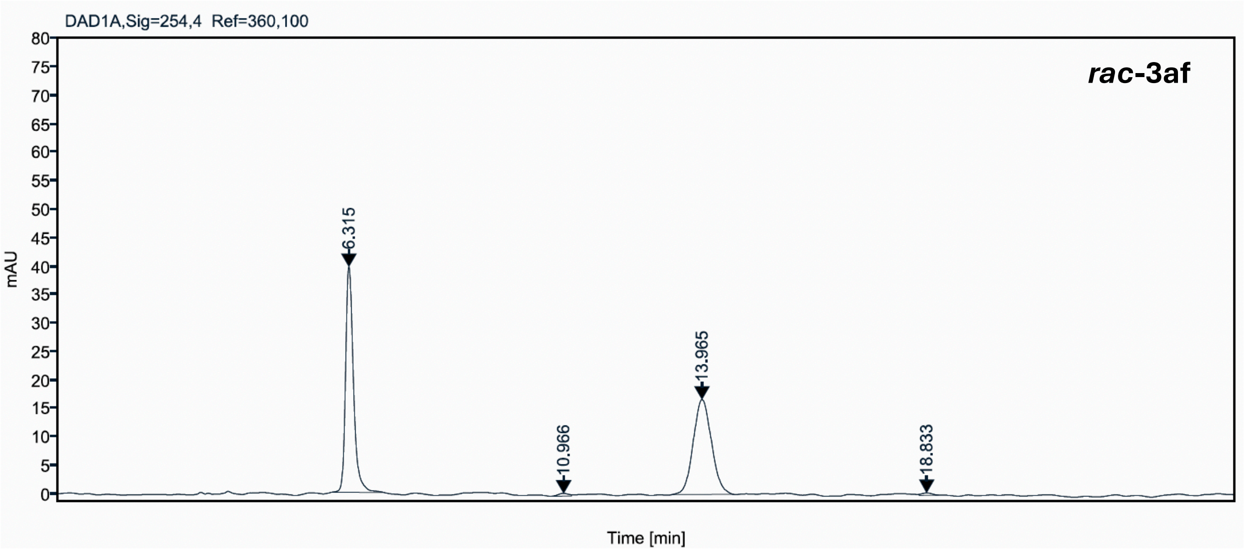

Signal: DAD1A,Sig=254,4 Ref=360,100

RT [min]	Type	Width [min]	Area	Height	Area%	Name
6.315	MM m	1.1331	465.1868	39.7027	49.4208	
10.966	MM m	0.4259	7.7130	0.4898	0.8194	
13.965	MM m	1.3376	461.7136	16.7080	49.0518	
18.833	MM m	0.4154	6.6645	0.4428	0.7080	
		**Sum**	**941.2779**			

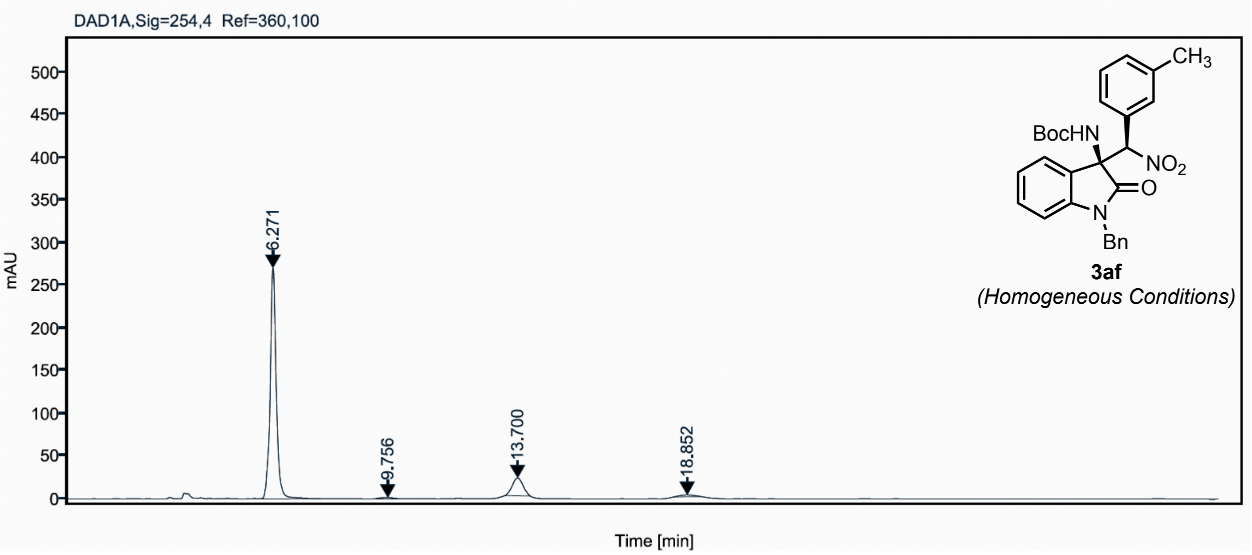

Signal: DAD1A,Sig=254,4 Ref=360,100

RT [min]	Type	Width [min]	Area	Height	Area%	Name
6.271	VB	1.8383	3477.5216	270.8633	85.9548	
9.756	MM m	0.7185	33.2768	1.3646	0.8225	
13.700	MM m	0.7614	467.8256	20.6148	11.5634	
18.852	MM m	0.8416	67.1341	2.0995	1.6594	
		**Sum**	**4045.7581**			

HPLC Chromatograms of ***rac*-3ag** and **3ag** under Homogeneous Conditions

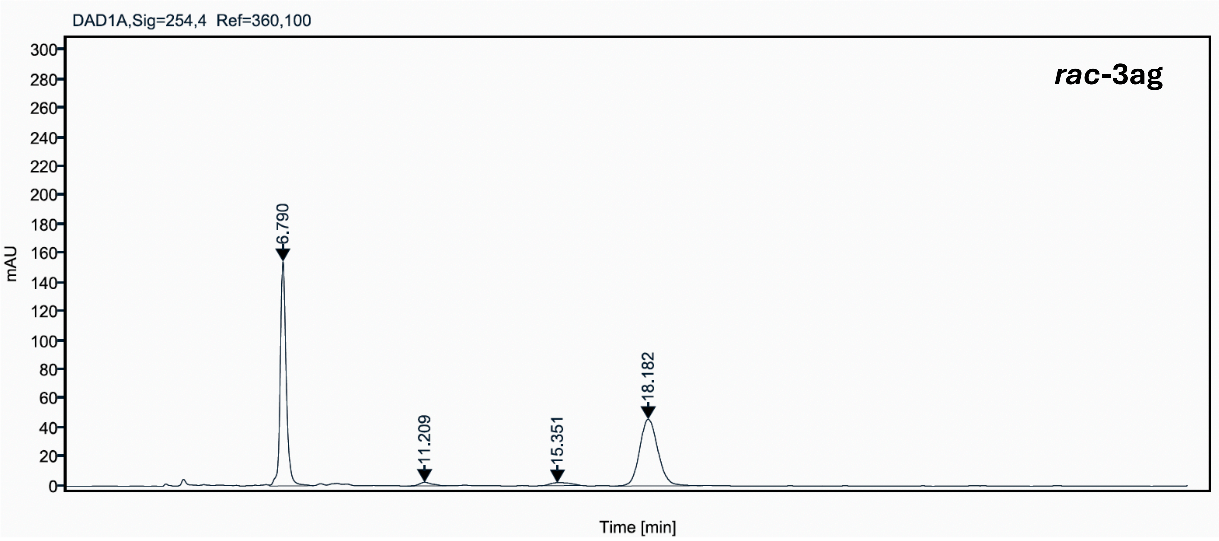

Signal:	DAD1A,Sig=254,4 Ref=360,100					
**RT [min]**	**Type**	**Width [min]**	**Area**	**Height**	**Area%**	**Name**
6.790	VB	1.3563	1980.9038	154.5424	50.0400	
11.209	BB	1.1962	71.8243	2.6218	1.8144	
15.351	MM m	1.2552	92.1021	2.0104	2.3266	
18.182	BB	2.6534	1813.8084	45.8960	45.8190	
		**Sum**	**3958.6386**			

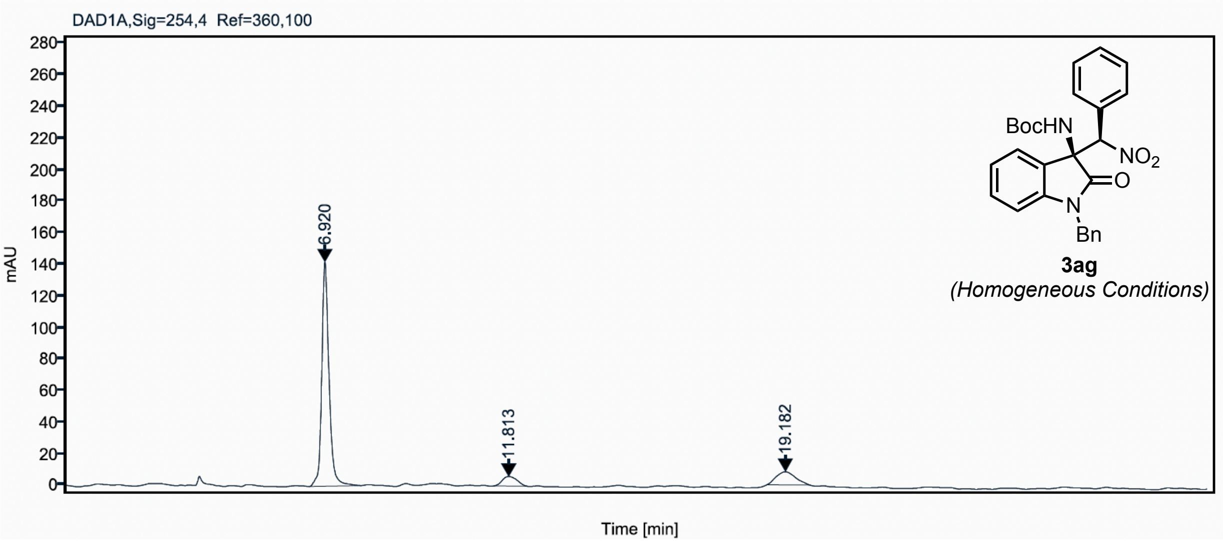

Signal:	DAD1A,Sig=254,4 Ref=360,100					
**RT [min]**	**Type**	**Width [min]**	**Area**	**Height**	**Area%**	**Name**
6.790	VB	1.3563	1980.9038	154.5424	50.0400	
11.209	BB	1.1962	71.8243	2.6218	1.8144	
15.351	MM m	1.2552	92.1021	2.0104	2.3266	
18.182	BB	2.6534	1813.8084	45.8960	45.8190	
		**Sum**	**3958.6386**			

HPLC Chromatograms of ***rac*-3ba** and **3ba** under Homogeneous Conditions

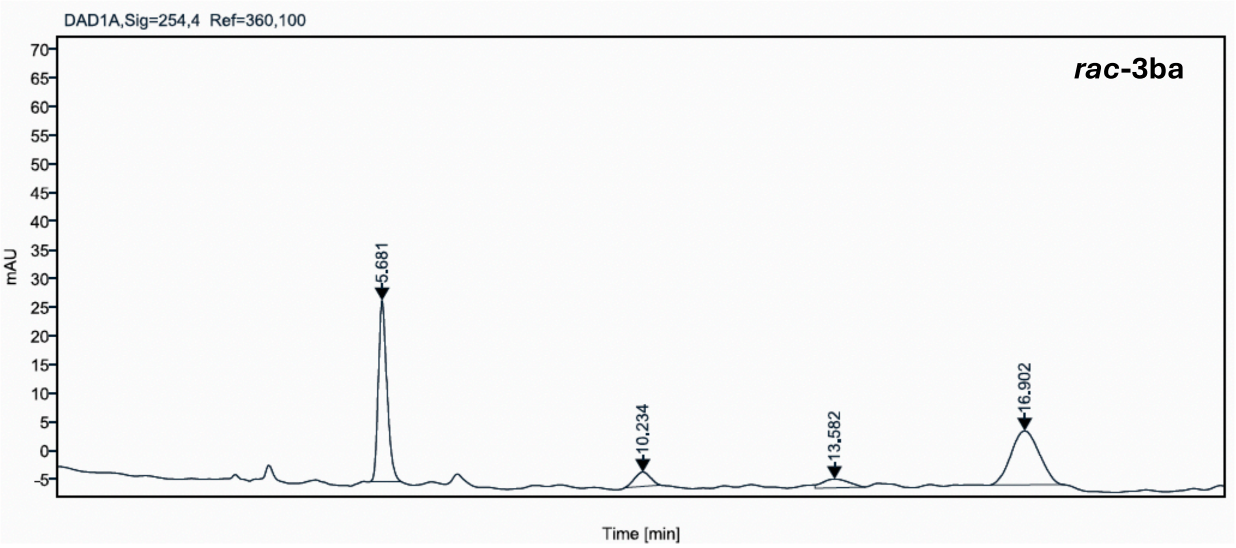

Signal: DAD1A,Sig=254,4 Ref=360,100

RT [min]	Type	Width [min]	Area	Height	Area%	Name
5.681	MM m	0.5340	332.9740	31.6201	44.1554	
10.234	MM m	0.6189	47.6675	2.4815	6.3211	
13.582	MM m	0.8696	47.8292	1.4884	6.3426	
16.902	BM m	1.3295	325.6244	9.4414	43.1808	
		Sum	754.0951			

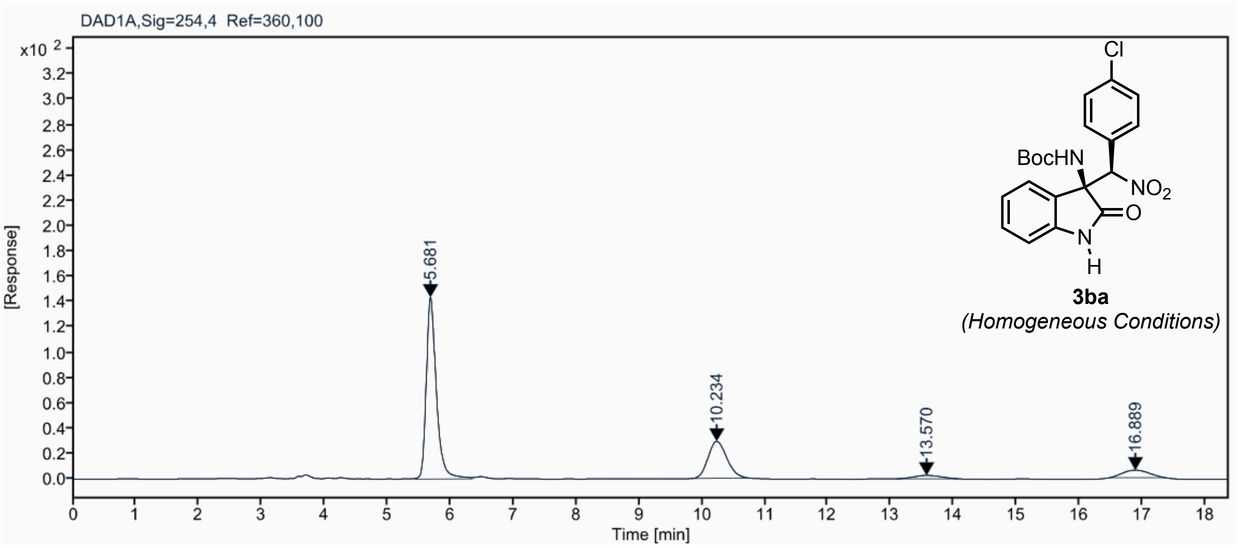

Signal: DAD1A,Sig=254,4 Ref=360,100

RT [min]	Type	Width [min]	Area	Height	Area%	Name
5.681	VV	0.9054	1607.6926	143.6397	65.1339	
10.234	MM m	0.8917	604.3380	29.1230	24.4841	
13.570	MM m	0.9809	80.2464	2.6974	3.2511	
16.889	MM m	0.8892	176.0127	5.9672	7.1310	
		Sum	2468.2897			

HPLC Chromatograms of ***rac*-3ca** and **3ca** under Homogeneous Conditions

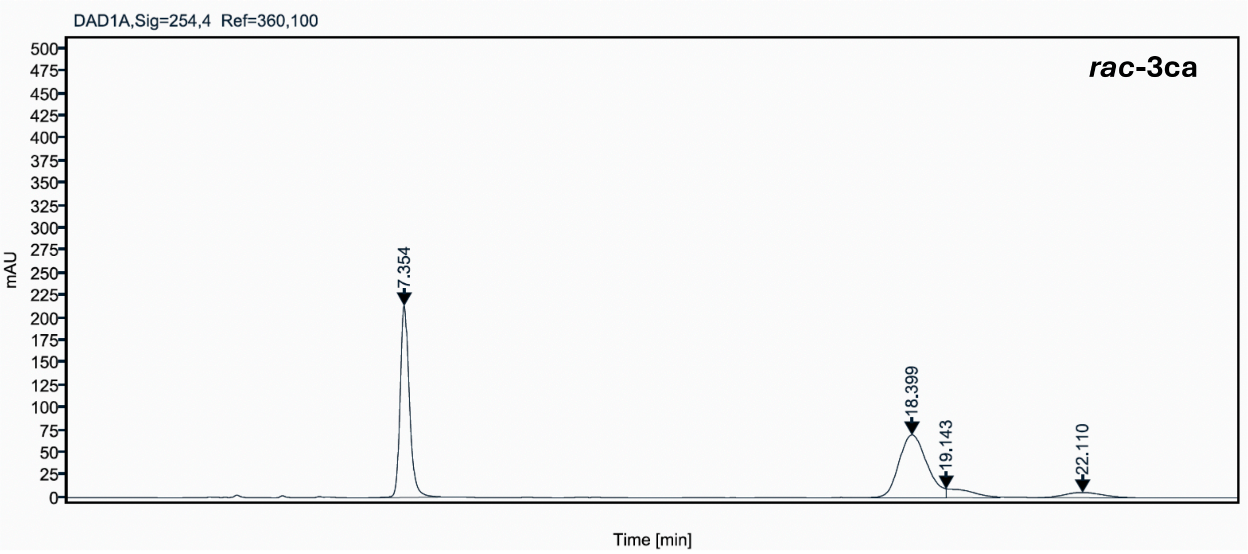

Signal: DAD1A,Sig=254,4 Ref=360,100

RT [min]	Type	Width [min]	Area	Height	Area%	Name
7.354	BB	1.8333	2954.3728	213.3747	44.6705	
18.399	MM m	1.6878	2935.9375	69.6934	44.3918	
19.143	MM m	1.1737	388.1201	9.6619	5.8684	
22.110	BB	2.2800	335.2642	5.7833	5.0692	
		**Sum**	**6613.6946**			

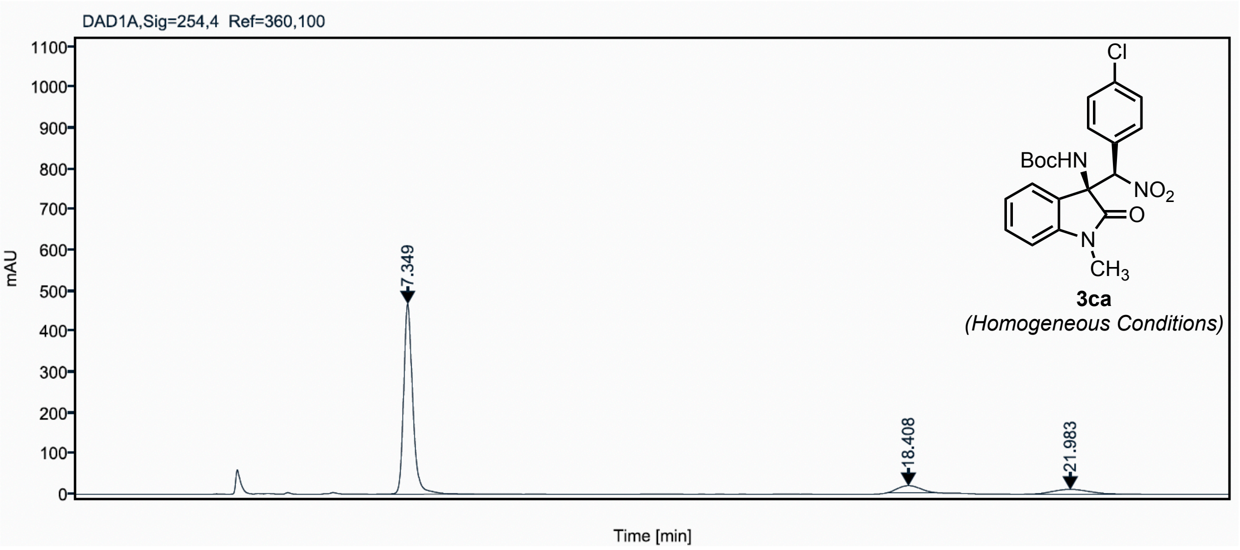

Signal: DAD1A,Sig=254,4 Ref=360,100

RT [min]	Type	Width [min]	Area	Height	Area%	Name
7.349	BB	2.2517	6588.5420	467.9217	84.2089	
18.408	MM m	1.0806	587.1998	17.1862	7.5051	
21.983	MM m	2.0751	648.3055	11.7778	8.2861	
		**Sum**	**7824.0473**			

HPLC Chromatograms of ***rac*-3da** and **3da** under Homogeneous Conditions

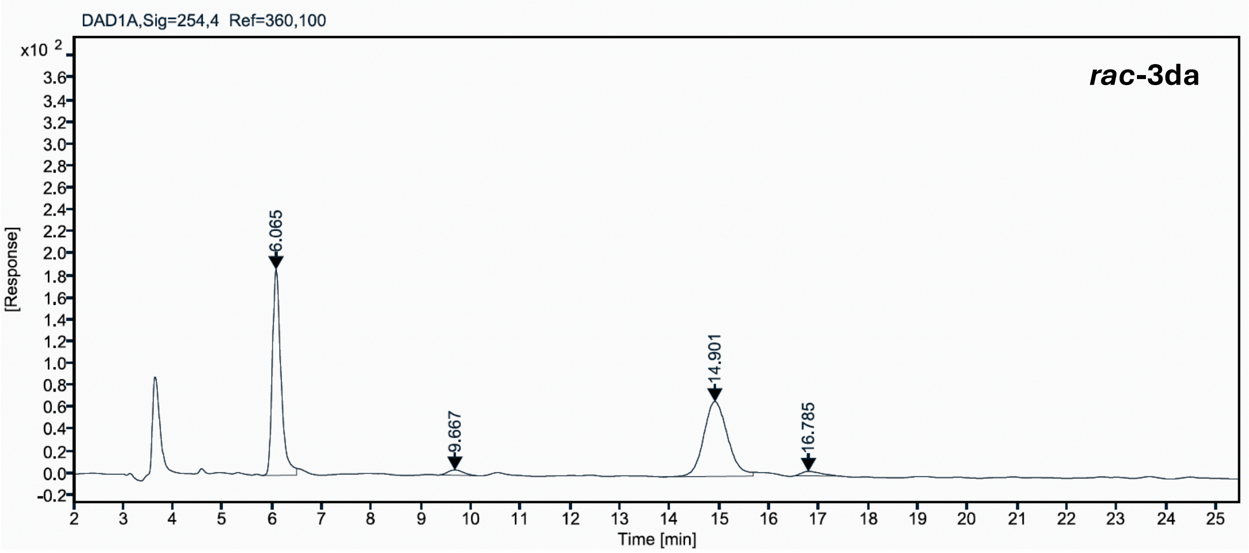

Signal: DAD1A,Sig=254,4 Ref=360,100

RT [min]	Type	Width [min]	Area	Height	Area%	Name
6.065	BM m	0.6807	2265.8425	187.6734	47.4841	
9.667	MM m	0.8011	106.8094	4.8415	2.2383	
14.901	BM m	1.8261	2285.6089	68.1960	47.8983	
16.785	MM m	0.9148	113.5323	4.1620	2.3792	
		**Sum**	**4771.7932**			

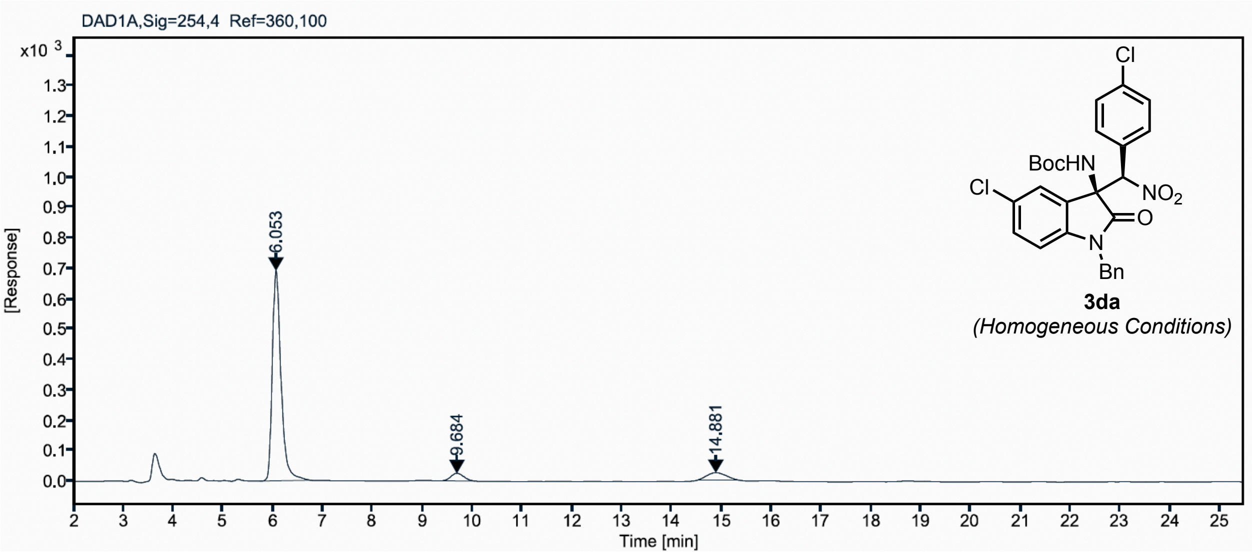

Signal: DAD1A,Sig=254,4 Ref=360,100

RT [min]	Type	Width [min]	Area	Height	Area%	Name
6.053	MM m	1.0398	8454.6251	692.0063	88.0484	
9.684	MM m	0.7267	454.8750	25.2400	4.7372	
14.881	MM m	0.8696	692.7442	24.9809	7.2144	
		**Sum**	**9602.2443**			

HPLC Chromatograms of ***rac*-3ea** and **3ea** under Homogeneous Conditions and Heterogeneous Conditions

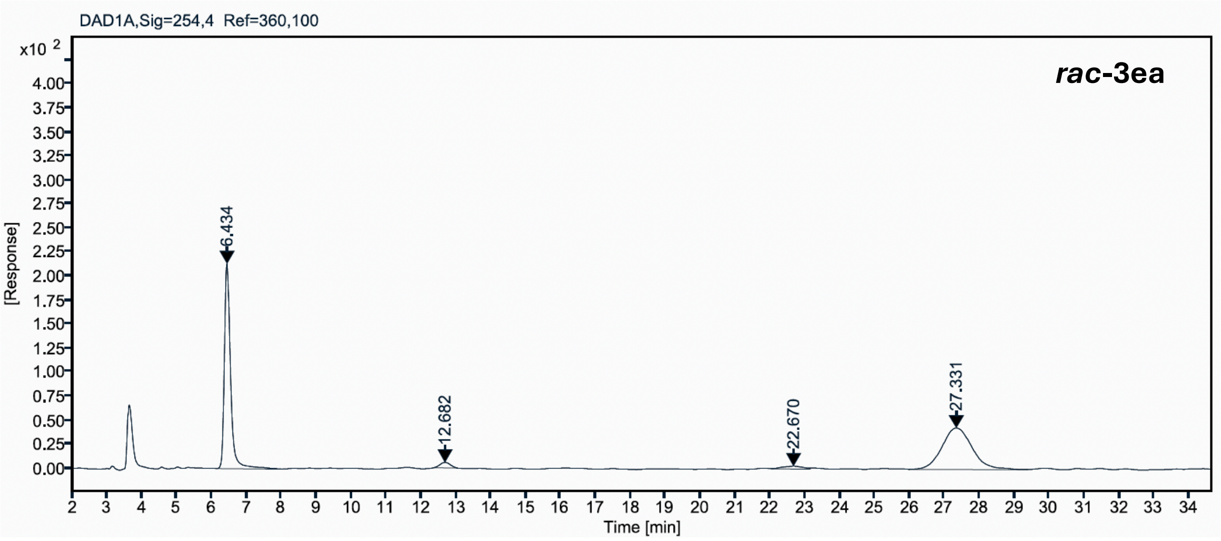

Signal: DAD1A,Sig=254,4 Ref=360,100

RT [min]	Type	Width [min]	Area	Height	Area%	Name
6.434	BB	1.8744	2632.1531	213.2906	47.9273	
12.682	MM m	0.6784	118.8344	5.7440	2.1638	
22.670	VM m	1.1249	119.9774	2.9563	2.1846	
27.331	VV	3.4039	2621.0051	43.2980	47.7243	
		**Sum**	**5491.9700**			

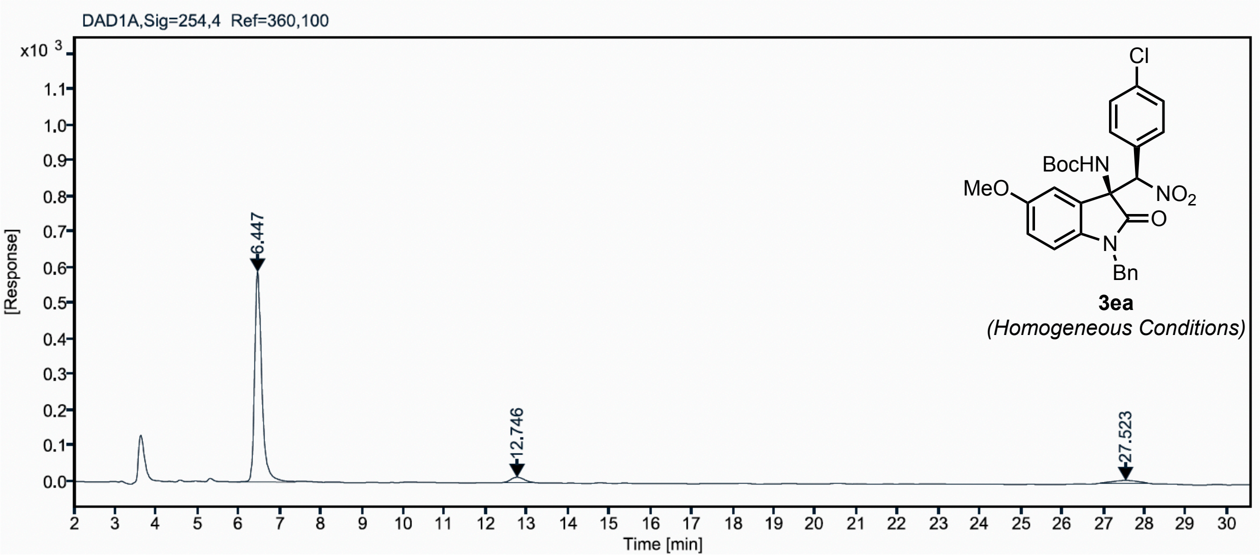

Signal: DAD1A,Sig=254,4 Ref=360,100

RT [min]	Type	Width [min]	Area	Height	Area%	Name
6.447	BV	1.4187	7209.5185	590.2951	90.5568	
12.746	MM m	0.8536	354.7475	15.4820	4.4559	
27.523	MM m	1.2765	397.0522	8.9413	4.9873	
		**Sum**	**7961.3183**			

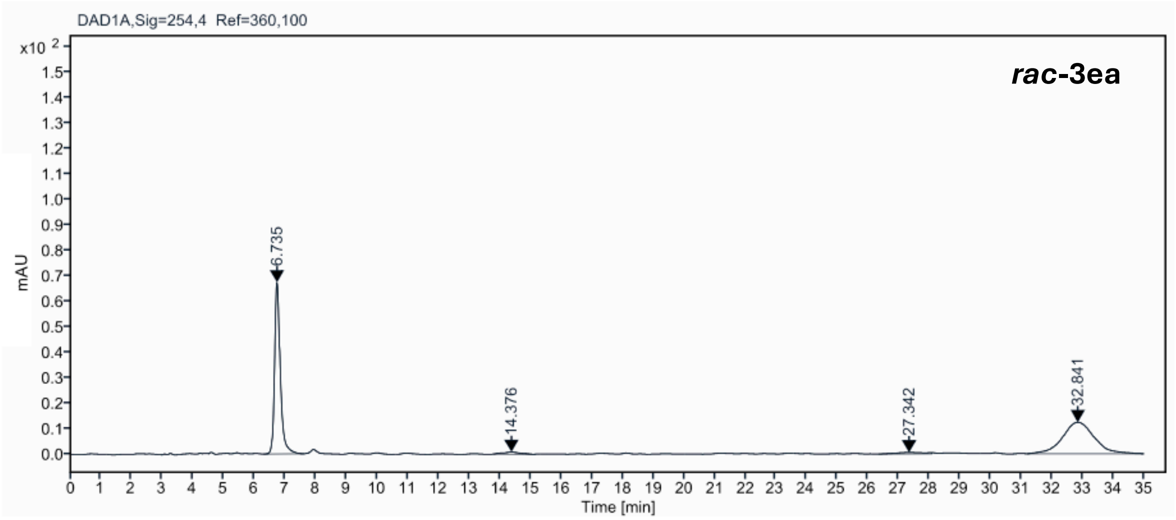

Signal: DAD1A,Sig=254,4 Ref=360,100

RT [min]	Type	Width [min]	Area	Height	Area%	Name
6.735	BB	1.2860	877.1031	67.2090	47.7306	
14.376	MM m	1.3328	30.1551	0.8055	1.6410	
27.342	MM m	1.7356	39.9361	0.6305	2.1733	
32.841	BBA	3.8067	890.4182	12.3240	48.4552	
		**Sum**	**1837.6125**			

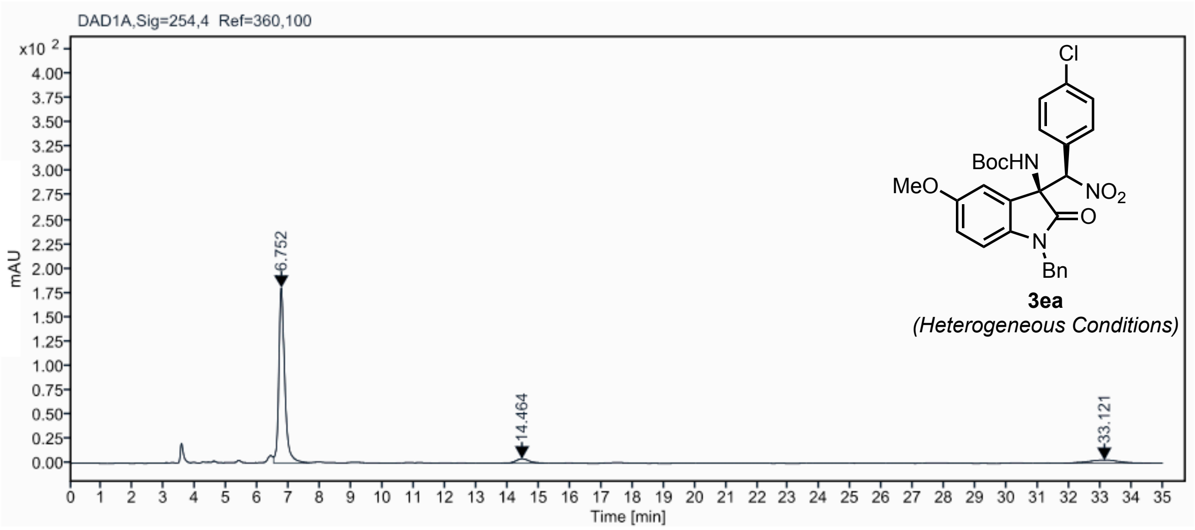

Signal: DAD1A,Sig=254,4 Ref=360,100

RT [min]	Type	Width [min]	Area	Height	Area%	Name
6.752	VV	1.1423	2367.2896	179.7514	87.0434	
14.464	MM m	1.2477	119.7617	4.3554	4.4035	
33.121	BB	2.7867	232.6147	3.3162	8.5531	
		**Sum**	**2719.6659**			

HPLC Chromatograms of ***rac*-3fa** and **3fa** under Homogeneous Conditions and Heterogeneous Conditions

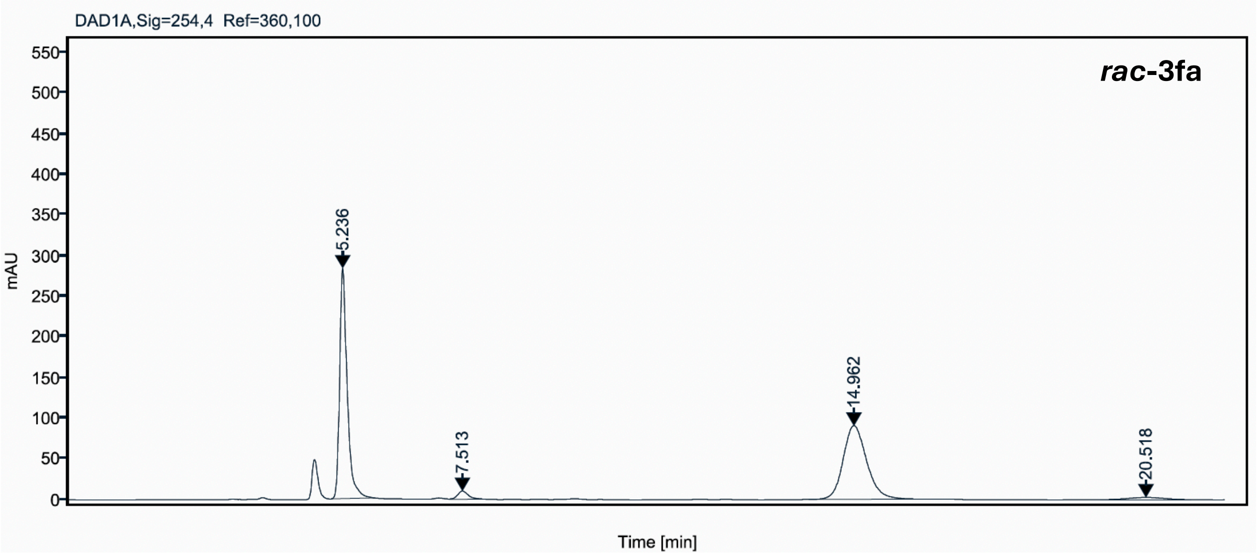

Signal: DAD1A,Sig=254,4 Ref=360,100

RT [min]	Type	Width [min]	Area	Height	Area%	Name
5.236	MM m	0.8093	2834.8384	283.1536	47.8027	
7.513	VM m	0.6384	141.1260	10.1038	2.3797	
14.962	MM m	2.1436	2818.1475	90.5779	47.5213	
20.518	BB	1.7177	136.1759	2.8953	2.2963	
		Sum	5930.2878			

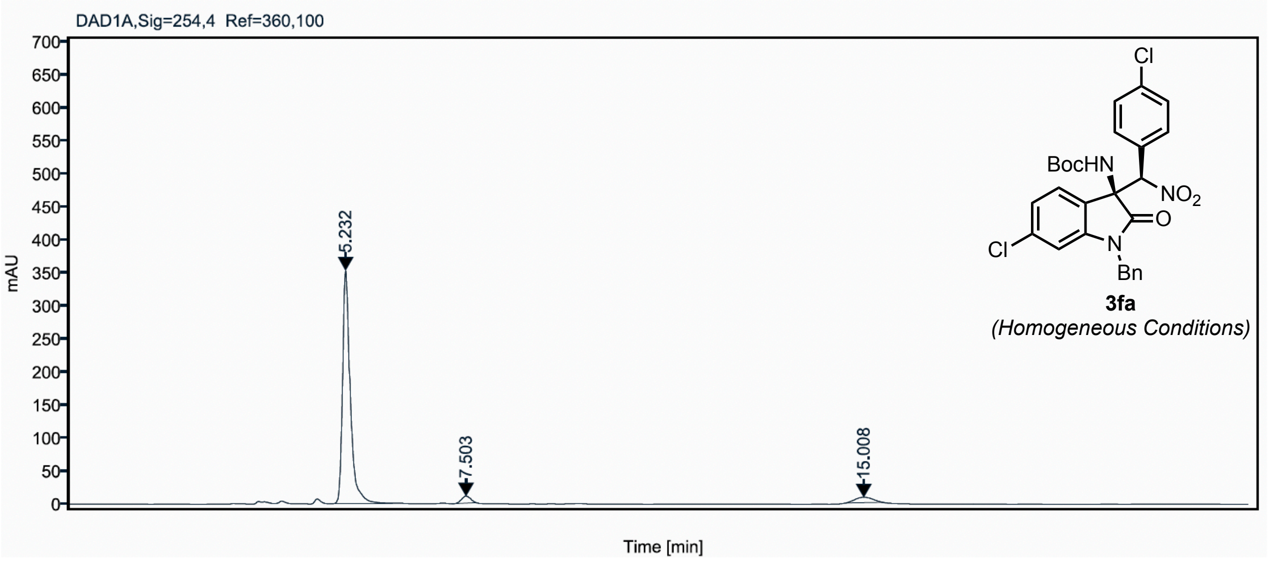

Signal: DAD1A,Sig=254,4 Ref=360,100

RT [min]	Type	Width [min]	Area	Height	Area%	Name
5.232	MM m	1.1656	3607.9725	352.8165	91.9124	
7.503	MM m	0.4705	126.2092	10.7663	3.2152	
15.008	MM m	0.6969	191.2655	8.2194	4.8725	
		Sum	3925.4472			

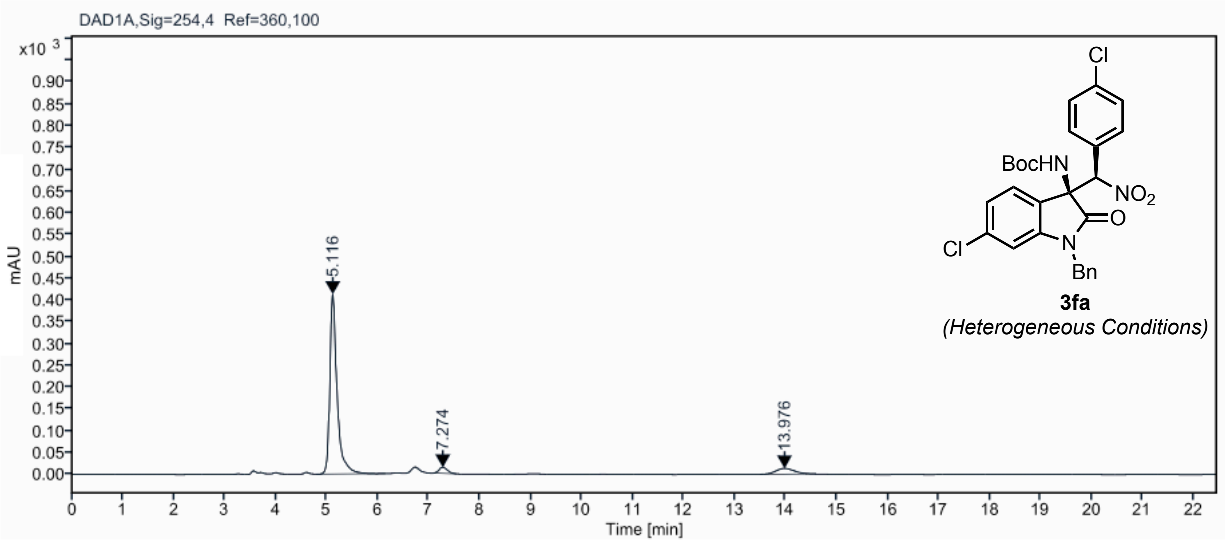

Signal: DAD1A,Sig=254,4 Ref=360,100

RT [min]	Type	Width [min]	Area	Height	Area%	Name
5.116	MM m	1.3309	4097.0379	412.7898	89.2589	
7.274	MM m	0.4167	154.9861	13.9547	3.3766	
13.976	MM m	1.0776	338.0356	13.2716	7.3645	
		**Sum**	**4590.0596**			

HPLC Chromatograms of ***rac*-3ga** and **3ga** under Homogeneous Conditions and Heterogeneous Conditions

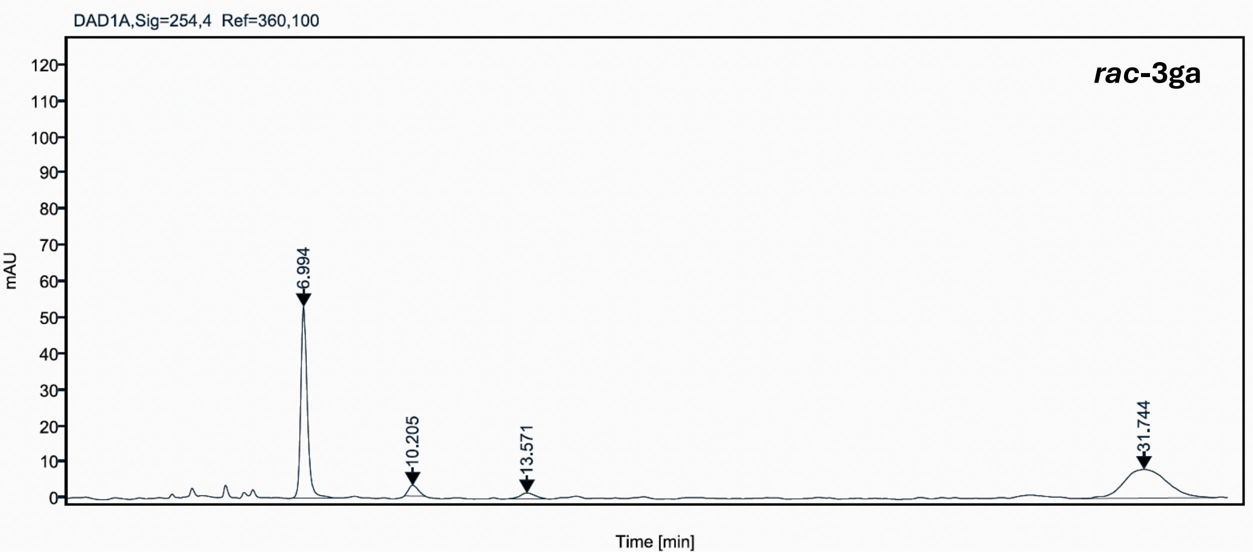

Signal:	DAD1A,Sig=254,4 Ref=360,100					
RT [min]	Type	Width [min]	Area	Height	Area%	Name
6.994	MB m	1.4047	704.3386	52.9397	46.3976	
10.205	MM m	0.6152	58.0522	2.9678	3.8241	
13.571	MM m	1.4715	53.7097	1.6859	3.5381	
31.744	BB	3.9163	701.9496	7.9429	46.2402	
		Sum	1518.0502			

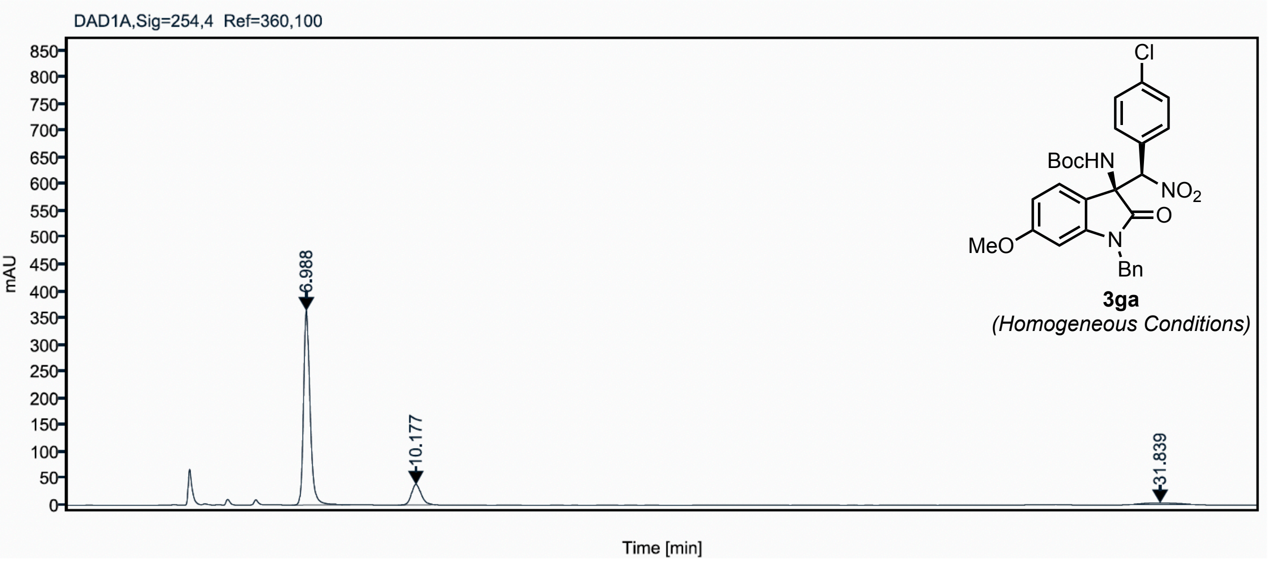

Signal:	DAD1A,Sig=254,4 Ref=360,100					
RT [min]	Type	Width [min]	Area	Height	Area%	Name
6.988	BB	1.6949	4847.8400	363.8451	82.9068	
10.177	MM m	1.0864	751.8555	38.2763	12.8581	
31.839	MM m	2.0473	247.6425	3.4019	4.2351	
		Sum	5847.3380			

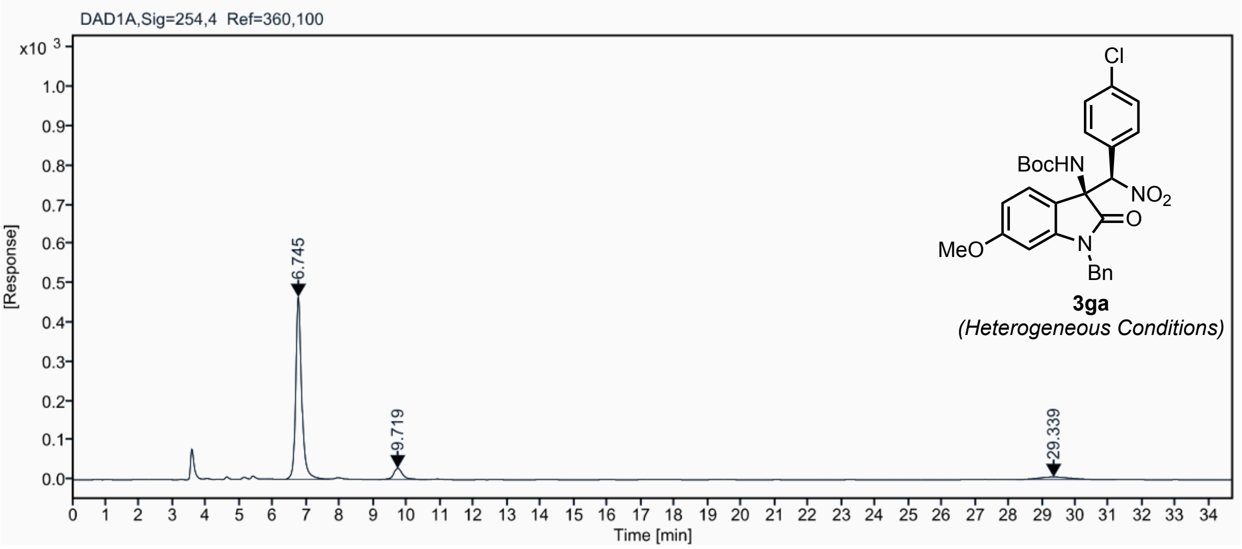

Signal: DAD1A,Sig=254,4 Ref=360,100

RT [min]	Type	Width [min]	Area	Height	Area%	Name
6.745	BV	1.3291	5868.7283	464.6369	85.5029	
9.719	BV	1.4648	547.8628	29.0045	7.9819	
29.339	BB	2.9800	447.1879	6.8063	6.5152	
		**Sum**	**6863.7790**			

HPLC Chromatograms of ***rac*-3bg** and **3bg** under Homogeneous Conditions

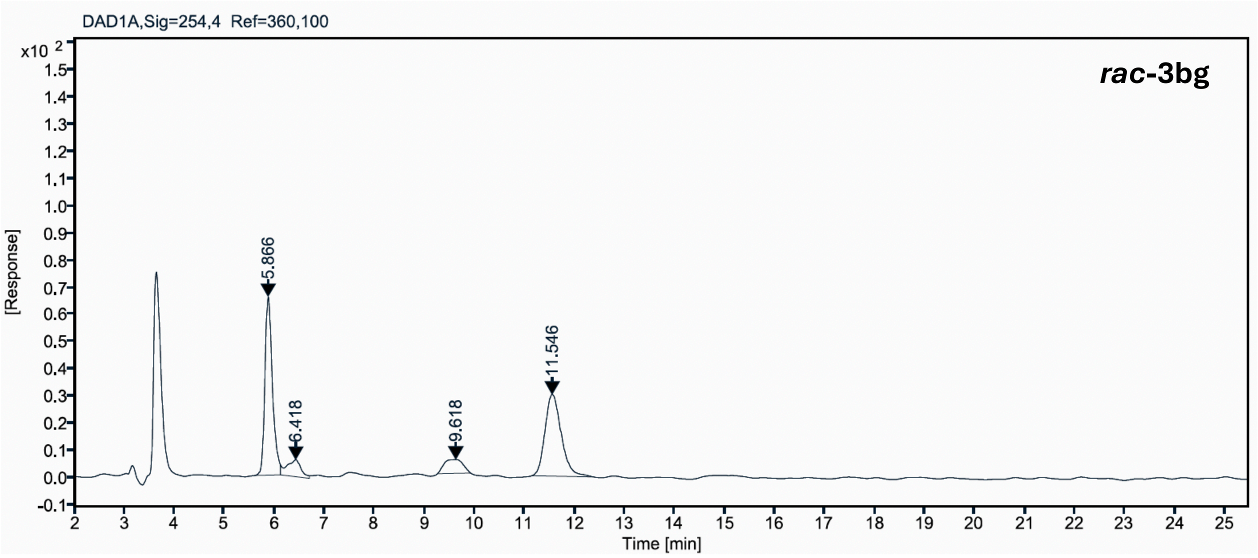

Signal: DAD1A,Sig=254,4 Ref=360,100

RT [min]	Type	Width [min]	Area	Height	Area%	Name
5.866	MM m	0.5082	683.9560	65.5071	42.2913	
6.418	MM m	0.5795	123.4639	6.1867	7.6342	
9.618	MM m	0.6605	131.9253	5.0583	8.1574	
11.546	MM m	1.2194	677.9051	29.9507	41.9171	
		**Sum**	**1617.2502**			

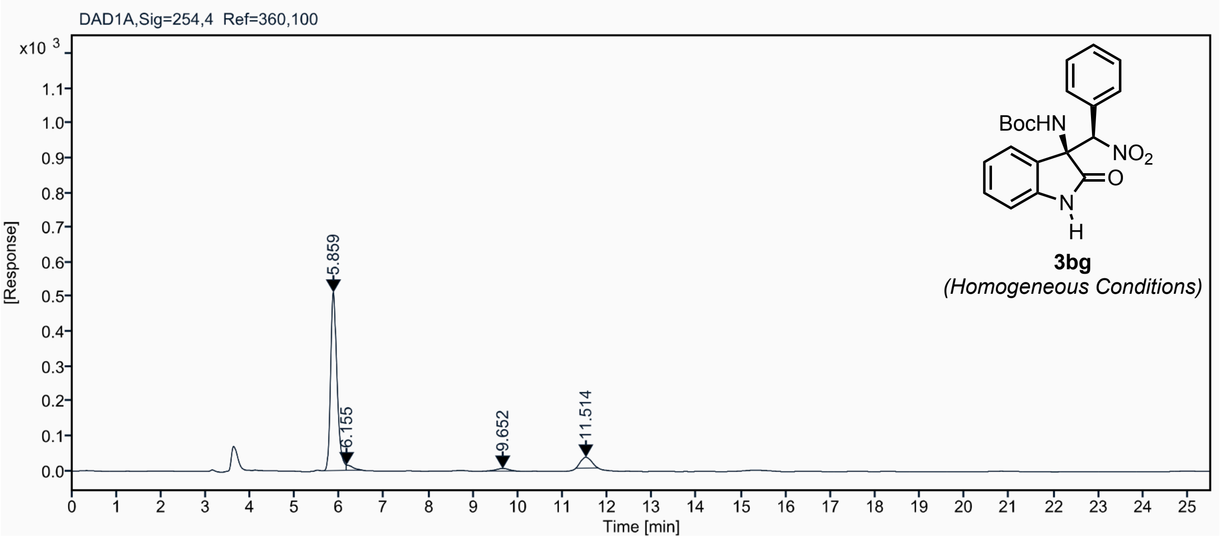

Signal: DAD1A,Sig=254,4 Ref=360,100

RT [min]	Type	Width [min]	Area	Height	Area%	Name
5.859	MM m	0.5386	5201.9205	512.5424	86.3436	
6.155	MM m	0.3603	148.1826	14.8030	2.4596	
9.652	MM m	0.7483	156.4590	7.6290	2.5970	
11.514	MM m	0.4989	518.1089	30.9856	8.5998	
		**Sum**	**6024.6709**			

HPLC Chromatograms of ***rac*-3ah** and **3ah** under Homogeneous Conditions

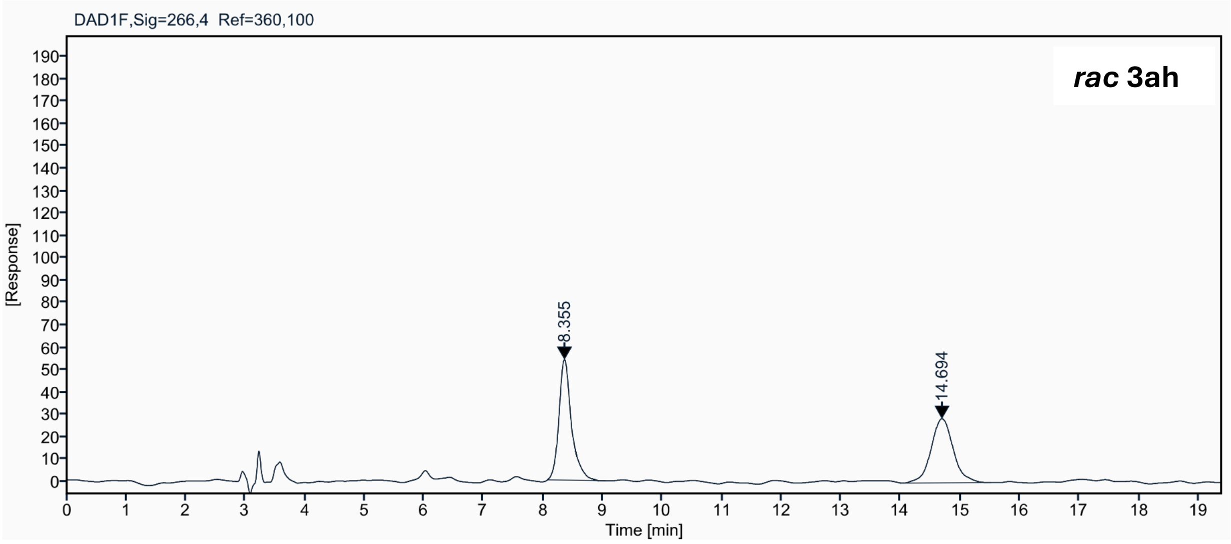

Signal: DAD1F,Sig=266,4 Ref=360,100

RT [min]	Type	Width [min]	Area	Height	Area%	Name
8.355	MM m	0.7219	838.0805	53.7710	50.5696	
14.694	MB m	1.7398	819.1999	29.2865	49.4304	
		**Sum**	**1657.2805**			

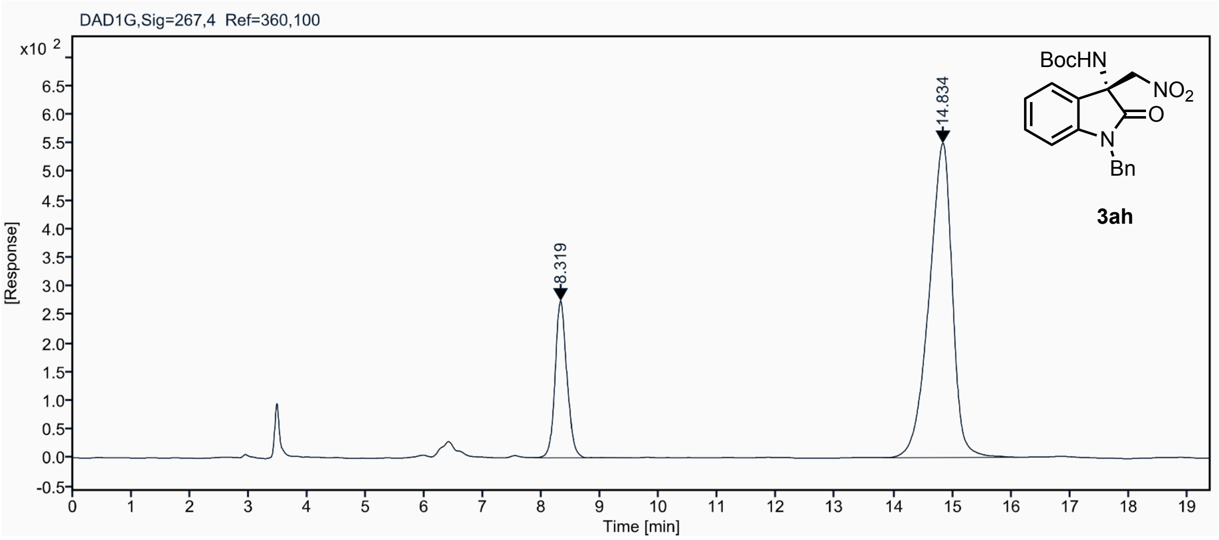

Signal: DAD1G,Sig=267,4 Ref=360,100

RT [min]	Type	Width [min]	Area	Height	Area%	Name
8.319	VB	1.6607	4390.2230	274.3291	22.5221	
14.834	VB	2.2884	15102.7751	549.0863	77.4779	
		**Sum**	**19492.9980**			
